# Product Selectivity Control in the Brønsted Acid-Mediated Reactions with 2-Alkynylanilines

**DOI:** 10.3390/molecules29153693

**Published:** 2024-08-04

**Authors:** Valerio Morlacci, Massimiliano Aschi, Marco Chiarini, Caterina Momoli, Laura Palombi, Antonio Arcadi

**Affiliations:** 1 Dipartimento di Scienze Fisiche e Chimiche, Università Degli Studi Dell’Aquila, Via Vetoio, 67100 Coppito, Italy; valerio.morlacci@graduate.univaq.it (V.M.); massimiliano.aschi@univaq.it (M.A.); caterina.momoli@graduate.univaq.it (C.M.); laura.palombi@univaq.it (L.P.); 2 Dipartimento di Bioscienze e Tecnologie Agroalimentari e Ambientali, Università Degli Studi di Teramo, Via R. Balzarini, 64100 Teramo, Italy; mchiarini@unite.it

**Keywords:** 2-alkynylanilines, annulations, sequential reactions, DFT calculations

## Abstract

Brønsted acid-catalysed/mediated reactions of the 2-alkynylanilines are reported. While metal-catalysed reactions of these valuable building blocks have led to the establishment of robust protocols for the selective, diverse-oriented syntheses of significant heterocyclic derivatives, we here demonstrate the practical advantages of an alternative methodology under metal-free conditions. Our investigation into the key factors influencing the product selectivity in Brønsted acid-catalysed/mediated reactions of 2-alkynylanilines reveals that different reaction pathways can be directed towards the formation of diverse valuable products by simply choosing appropriate reaction conditions. The origins of chemo- and regioselectivity switching have been explored through Density Functional Theory (DFT) calculations.

## 1. Introduction

**Scheme 1:**
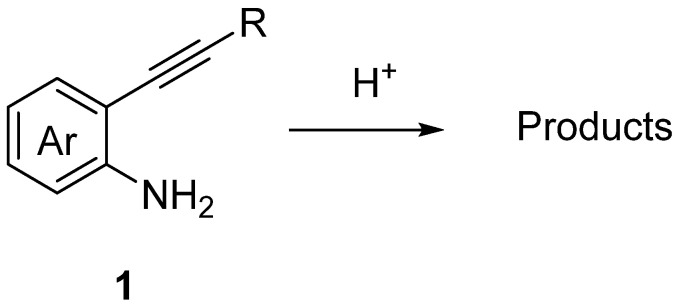
Brønsted acid-catalysed/mediated reaction of 2-alkynylanilines **1**.

The 2-alkynylanilines **1**, readily available via Sonogashira cross-coupling of 2-haloanilines with terminal alkynes [1,2], are growing in importance as building blocks for constructing a wide variety of heterocyclic scaffolds [3,4,5]. Significantly, these substrates enable access to challenging target skeletal diversity with good atom economy through product selectivity control. Indeed, controlling chemoselectivity also avoids the tedious separation of the desired reaction product from unwanted compounds, thereby further reducing waste and the cost of the process. Therefore, there is a growing demand for developing alternative strategies with high selectivity for producing pharmaceuticals and fine chemicals [6]. In particular, the overcoming of the drawbacks of conventional methods [7] in the synthesis of pharmaceutical agents [8] from 2-alkynylanilines is relevant in academia and pharmaceutical industries due to their various biological activities including anticancer [9] and anti-neurodegenerative agents [10]. Moreover, cascade synthetic approaches continue to be a focus of intensive research due to their unique features of reducing the time and labour of the synthetic process and their potential to rapidly increase molecular complexity in a single operation [11]. Our ongoing interest in the synthesis of target heterocyclic scaffolds from aminoalkynes [12], encouraged us, supported by computational insights, to explore Brønsted acid-catalysed/mediated reactions of 2-alkynylaniline derivatives [13,14] as viable alternatives to the transition-metal catalysed methods (Scheme 1) [15,16,17,18,19].

The experimental results of this investigation, along with computational calculations aimed at rationalising the product selectivity control of these reactions, are set out below.

## 2. Discussion

**Scheme 2:**
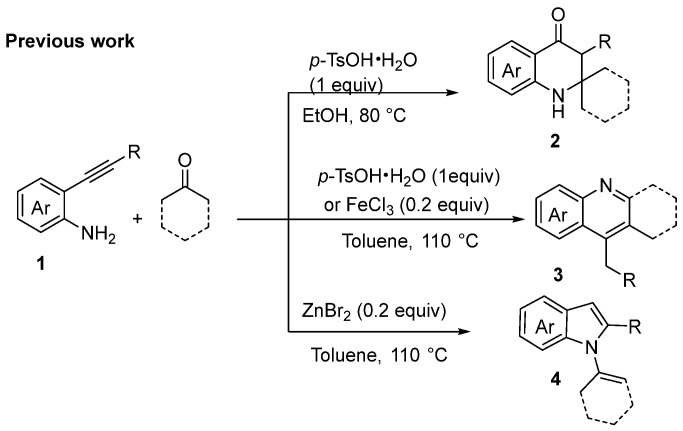
Product selectivity control in the sequential reaction of 2-alkynylanilines **1** with ketones.

Based on our investigations on divergent sequential reactions of the starting building blocks **1**, we have previously identified the key factors able to direct their reaction with ketones towards the selective synthesis of 2,2,3-functionalised-2,3-dihydroquinolin-4 (1*H*)-ones **2**, functionalised quinolines **3**, or *N*-alkenyl indoles **4** (Scheme 2) [20].

**Scheme 3:**
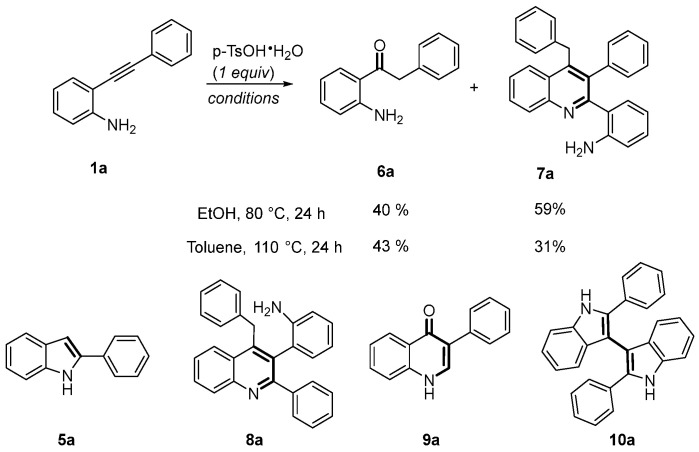
Hydration vs dimerization reaction of **1a**. Other products are given in Table 1.

During this study, side-reactions leading to the 2-aminoarylketone **6a**, derived by the hydration of the C-C triple bond of **1a**, and to the highly dense quinoline **7a**, derived by its regioselective dimerization, were also observed. Yet, two control experiments highlighted that *p*-TsOH·H_2_O promotes the formation of **6a** and **7a** in both EtOH at 80 °C and Toluene at 110 °C, but a different ratio between the two derivatives is observed. (Scheme 3). These findings suggested the possibility of directing the selectivity of the process through careful tuning of the reaction conditions. Consequently, we decided to explore the reactivity of the 2-phenylethynyl aniline **1a** using Brønsted acids as promoters under a variety of reaction conditions.

The results of our investigation are summarised in Table 1. As shown, reaction parameters such as the temperature, reaction medium, nature of the Brønsted acid, and its loading have a significant effect on directing the regio- and chemoselectivity of the reaction. Performing the reaction in EtOH at 110 °C with a stoichiometric amount of *p*-TsOH·H_2_O favoured the prevalent regioselective dimerization of **1a** over its hydration, leading to 2-(2-aminophenyl)quinoline **7a** with a good yield of 70% (**7a**/**8a** = 80/20) (entry 1). Surprisingly, reducing the loading of *p*-TsOH·H_2_O resulted in a significant decrease in chemoselectivity, and 1-(2-aminophenyl)-2-phenylethan-1-one **6a** was isolated with a 46% yield nearly comparable to **7a** (entry 2). The use of methanesulfonic acid (MsOH) as a promoter afforded a mixture of **7a** and the regioisomeric 3-(2-aminophenyl)quinoline **8a** in an overall yield of 53% (Table 1, entry 3). Instead, the reaction mediated by trifluoromethanesulfonic acid (TfOH) yielded 2-phenylindole **5a** along with the dimerization products **7a**/**8a** (entry 4). Additionally, in this case, a partial inversion of regioselectivity was observed, with **8a** predominating over **7a** (entry 4 vs. entries 1 and 3). Similar regioselectivity, with a slight increase in the overall yield of **7a**/**8a**, was observed when EtOH was replaced with Toluene as the reaction medium (entry 5). By contrast, the change of solvent was critical in the case of the *p*-TsOH·H_2_O acid-mediated reactions, where the use of i-PrOH instead of EtOH led to a drastic decrease in chemo- and regioselectivity (entry 6). Intriguingly, *p*-TsOH·H_2_O acid caused a puzzling change in reactivity when switching from the polar protic solvent to the halogenated solvent DCE. In fact, albeit in a moderate 54% yield, the 2-phenylindole **5a** (along with 34% of recovered starting material **1a**) was isolated as the sole product when performing the reaction at 40 °C in the presence of 0.2 equiv. of the *p*-TsOH·H_2_O (entry 7). Conversely, under identical temperature and solvent conditions, a stoichiometric amount of *p*-TsOH·H_2_O selectively led to hydration product **6a** (entry 8). In the presence of 0.2 equiv. of *p*-TsOH·H_2_O, the formation of dimer **8a** increased steadily as the temperature increased (entries 9–10). Yet, in DCE at 110 °C, a stoichiometric amount of *p*-TsOH·H_2_O promoted the formation of **8a** in an appreciable 48% yield, along with a smaller amount of the regioisomeric quinoline **7a,** which was obtained to a lower extent (19%) (entry 11).

While in THF, poor reactivity was observed as it resulted in the recovery of 56% of the starting material and a 15% yield of **6a** (entry 12). Interesting results were achieved by performing the reaction in DMF. In this latter solvent, the dimerization of **1a** was completely suppressed; however, in addition to **5a** (45%) and **6a** (22%), the formation of 3-phenylquinolin-4 (1*H*)-one **9a** was observed to a significant extent (30%). This result suggested that, under these conditions, DMF can act as a C1 synthon [21]. The 2-amino phenyl ketones **6** were reported to afford the phenylquinolin-4 (1*H*)-one **9** by means of sequential iron (III)-catalysed oxidation of alcohol/methyl arene, followed by condensation with amine/Mannich-type cyclisation/oxidation to complete the 4-quinolone ring [22].

Finally, in an attempt to improve the chemoselectivity of the reaction and rationalise the formation of **9a,** we also attempted the reaction in DCE/Dimetoxymethane (DMM) [23,24]. However, under the condition reported in entry 14, instead of **9a**, the selective formation of the bis-indole **10a** occurred with a satisfactory 76% yield (entry 14) [25].

To shed light on the diverse reaction pathways, quantum chemical calculations were performed in the framework of Density Functional Theory using the wB97XD functional, making use of a version of Grimme’s D2 dispersion model [26] in conjunction with the 6-31G* basis set. [27] Notably, test calculations performed on some of the critical points (see below) using a larger basis set (6-311 + G*) showed similar results. All the critical points were located and characterised as true minima or first-order saddle points (hereafter Transition Structures, TS) through the calculations of the molecular vibrations in harmonic approximation. The eigenvectors with negative eigenvalues, obtained from the diagonalisation of the mass-weighted Hessian matrix, were also used to prove the actual role of each TS along the reaction route. The bulk effect of the solvent, either ethanol (EtOH) or 1,2-dichloroethane (DCE), was included in calculations using the mean-field approximation through the Polarisable Continuum Model [28] as implemented in the Gaussian16 software C.01. [29]. Only in the case of EtOH (see below), the effect of one explicit solvent molecule was taken into account. The standard molar free energy was calculated for each species using standard statistical mechanical relations based on harmonic frequencies, moments of inertia, and excess free energies as obtained from DFT calculations. The reference state was established at 1.0 mol per litre for all species, except for the solvent, where its experimental density under specific experimental conditions was utilized. For rationalising the energy differences that emerged in the different reaction routes, additional calculations were performed at the same level of theory on some of the key transition structures and reaction intermediates using the Non-Covalent Interaction Index (NCI) analysis based on promolecular density [30] and RESP atomic charges [31]. Both of these analyses were performed with the Multwfn program [32].

The optimised geometries and harmonic frequencies of all species are provided in the Supplementary Information.

**Figure 1:**
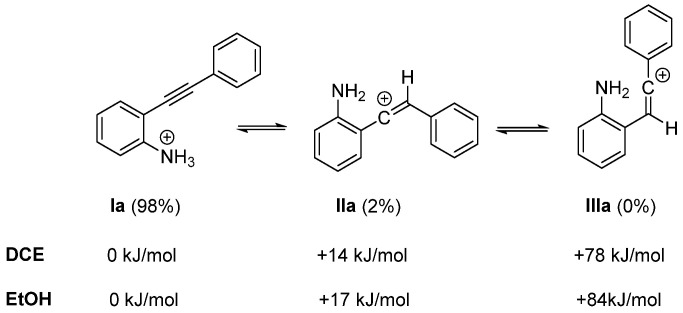
Protonation site distribution and relative free-energies of **Ia**, **IIa**, and **IIIa** in DCE and EtOH.

We began the study by identifying the preferred protonation site in **1a** and assessing the relative stability of the N-protonated species (**Ia**) and the two C-protonated species (**IIa** and **IIIa**) depicted in Figure 1 [33].

As expected, calculations indicated that the *N*-protonated species **Ia** is, by far, the most abundant protomer, with a relative population exceeding 98% at 110 °C in both solvents at equilibrium. In fact, compared to **IIa**, the free energy of the anilinium ion **Ia** is lower by −14 kJ/mol (in DCE) and −17 kJ/mol (in EtOH) and lower by −78 kJ/mol (in DCE) and −84 kJ/mol (in EtOH) when compared to **IIIa**. Notably, these findings effectively explain the observed formation of the (insoluble) anilinium salt **Ia** when a stoichiometric amount of Brønsted acid is used.

**Figure 2:**
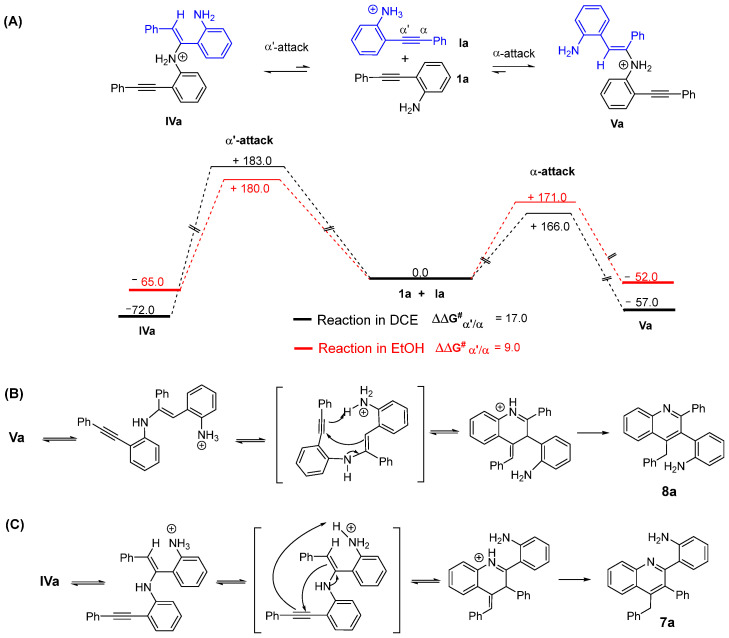
(**A**) Reaction scheme for **1a** dimerization under acid conditions and relative free energies in kJ/mol at 110 °C in DCE (black lines) and EtOH (red lines). (**B**) Possible reaction pathways for the formation of **8a** and (**C**) **7a**.

To rationalise the distribution of the products under the various conditions of Table 1, we proceeded by calculating the energy profiles for the **1a** dimerization reaction in DCE and EtOH (Figure 2). When the reaction is performed in DCE, the addition of **1a** to **Ia** can originate the regioisomeric intermediates **IVa** (Figure 2, α’-attack, black lines) and **Va** (Figure 2, α-attack, black lines), with the latter species being largely preferred from a kinetic point of view with a ∆∆G^#^ of 17 kJmol. **Va** should be likely responsible for the formation of the regioisomer **8a** isolated as the major product under the conditions of Table 1, entry 11.

By computing the reaction in EtOH as a solvent, the formation of **Va** remains kinetically favourable, albeit with a lower ∆∆G^#^ of 9 kJmol (Figure 2, red lines). Therefore, the selective formation of regioisomer **7a** under the conditions described in Table 1, entry 1, appears to be justified only if these conditions favour thermodynamic control of the reaction.

**Scheme 4:**
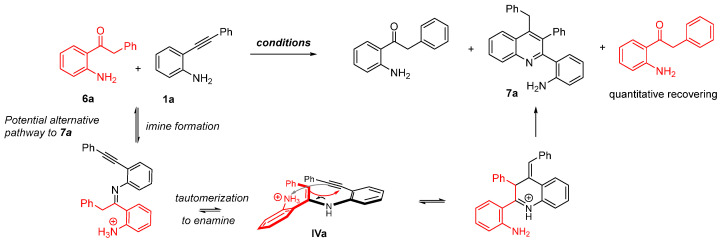
Control experiment ruling out the formation of **7a** from **6a** (Reaction conditions: Table 1, entry 1).

It has to be noted that a potential alternative reaction pathway to **7a** through the condensation of **1a** with **6a** to give **IVa** was ruled out by the control experiment illustrated in Scheme 4 [34]. In fact, the ketone **6a**, intentionally added to the reaction mixture at the beginning, was quantitatively recovered at the end of the reaction.

**Scheme 5:**
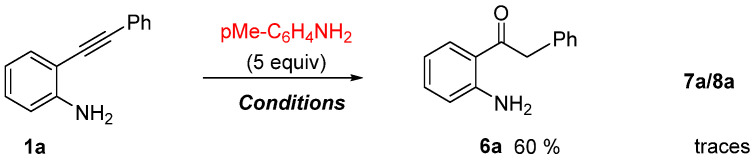
Control experiment to suppress the dimerization of **1a** (Reaction conditions: Table 1, entry 1).

Moreover, a further control experiment conducted by using *p*-toluidine as a proton scavenger confirmed the need for the presence of both **1a** and anilinium salt **Ia** to eventually achieve the dimer **7a** under the Brønsted acid-promoted reaction in EtOH at 110 °C (Scheme 5).

**Figure 3:**
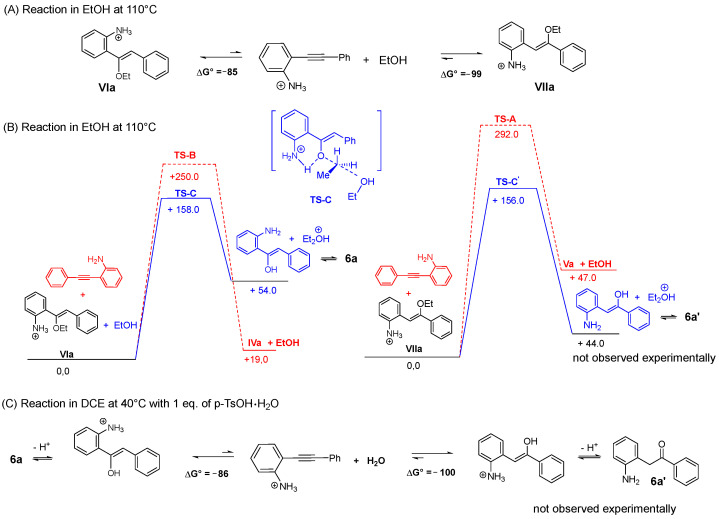
Possible reaction pathways and relative free energies in kJ/mol in EtOH (110 °C) and DCE (40 °C).

On the other hand, from a theoretical point of view, other scenarios become possible in EtOH. In fact, as shown in Figure 3, in this solvent, another quite irreversible addition of an alcohol molecule could take place through a reaction with a very negative ΔG°, yielding the two enol ethers **VIa** and **VIIa** (with the H and OEt groups in nearly degenerate syn or anti positions; see Supplementary Material for details) (Figure 3A).

Although the formation of **IVa** and **Va** from these intermediates is still conceivable, albeit with very high barriers (respectively 250 kJ/mol for **IVa** and 292 kJ/mol for **Va** (Figure 3B red lines), reactive channels with significantly lower energy profiles through the **TS-C** and **TS-C’** have been identified starting from **VIa** and **VIIa** (Figure 3B blue lines). The proximity of the ammonium and OEt groups in these intermediates allows the proton to transfer, facilitating the subsequent formation of the enol by the nucleophilic attack of a second EtOH molecule. Analogous pathways towards the hydration products could be found for the reaction in DCE at 40 °C (Figure 3C). While these findings help to rationalise the acid-catalysed hydration of the triple bond (Table 1, entries 1–2 and 8), conversely to the Au-catalysed one [35], the origin of the high regioselectivity of the hydration reaction (only the ketone **6a** is observed experimentally in both EtOH and DCE) remains to be clarified.

**Figure 4:**
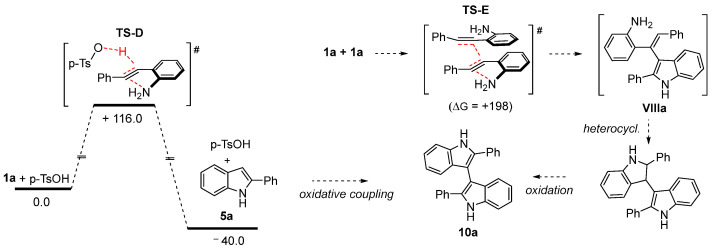
Reaction pathways to **5a** (Reaction conditions: Table 1, entry 7) and **VIIIa** (Reaction conditions: Table 1, entry 14) with relative free energies (kJ/mol) at 40 °C in DCE.

As indicated below (Figure 4, left side), DFT calculations also provided the possible mechanism producing the indole **5a,** which was obtained with the maximum yields of 54% under the conditions reported in Table 1, entry 7. Based on calculations, the formation of **5a** is expected by a concerted pathway involving the proton transfer from *p*-TsOH to **1a** with sinchronous cyclisation, through **TS-D**. The formation of the indole **5a** could also account for the attainment of the bis-indole **10a** by oxidative coupling when performing the reaction under the condition reported in Table 1, entry 14, where the in situ-generated formaldehyde could act as an oxidant [36]. On the other hand, DFT calculations suggest the intermediate **VIIIa** as an alternative possible precursor of the bis-indole **10a**. The relative energy profile leading to **VIIIa** is indicated in Figure 4, right side.

This Brønsted acid-catalysed domino cycloisomerization/coupling/oxidation to furnish pharmaceutically relevant 3,3′-biindolyl products may represent an alternative to the heterogeneous cycloisomerization of 2-alkynylanilines to indoles catalysed by carbon-supported gold nanoparticles and subsequent homocoupling to 3,3′-biindoles [25]. Substrate-controlled regioselective synthesis of 3,3′-bisindole derivatives has been also established by the *p*-TsOH·H_2_O catalysed reaction of 2-indolylmethanols with indoles of CHCl_3_ [37].

**Scheme 6:**
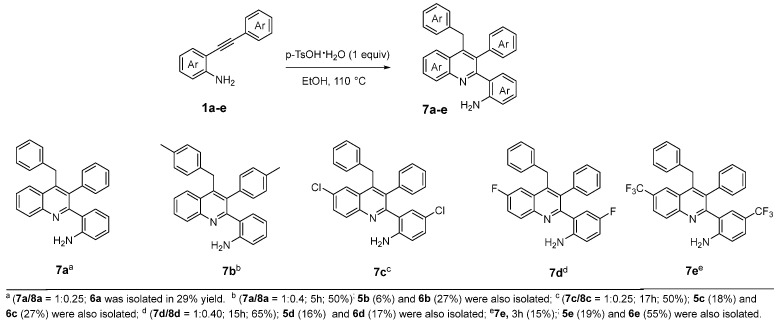
Synthesis of 2-(2-aminophenyl)quinoline derivatives **7**.

To test the generality of the procedure leading to the quinolines **7**, selected 2-arylalkynylanilines **1b–e** were reacted under the optimized conditions reported in Table 1, entry 1. As indicated in Scheme 6, expected 2-(2-aminophenyl)quinolines **7b–d** were predominantly obtained in satisfactory yields under aerobic conditions. The analogous regioselective outcome was reported in the Brønsted acid-promoted sequential hydration/condensation/double cyclization of pyridine-substituted 2-alkynylanilines [38]. Only the 2-arylalkynylaniline **1e** bearing the strong withdrawing *p*-CF_3_ substituent in the aniline moiety gave in our cases as a main product the carbonyl derivative **5e** [39]. It is worth noting that the dimerization of 2-alkynylanilines into the corresponding 2-(2-aminophenyl)quinoline derivatives **7** was previously reported to occur only under anaerobic conditions via a Bi (OTf)_3_/MesCOOH-catalysed process, with the outcome being highly dependent on the solvent used. In fact, in such a procedure, hexafluoroisopropanol was found to be a crucial solvent and promoter for the success of the reaction [40].

**Scheme 7:**
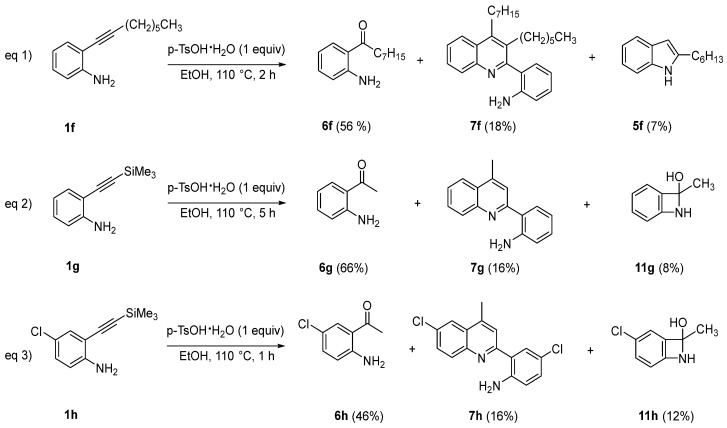
Hydration vs. dimerization of 2-alkynyl and 2-ethynyltrimethylsilyl anilines in EtOH at 110 °C.

However, in our procedure, the divergent hydration reaction also prevailed by performing the reaction on the alkyl and the trimethylsilyl-substituted alkynylanilines (Scheme 7). Conversely, the 2-phenyl-4-methyl-2-(2-aminophenyl)quinoline **7g,h** was isolated in high yield through the Bi(OTf)_3_/MesCOOH-catalysed or InBr_3_ promoted processes of 2-ethynylanilines **1g,h** [41]. Moreover, gold(III)/silver (I)-based catalysts [42], heterobimetallic catalysts of the type CpRu(PPh_3_)Cl(μ-dppm)AuI [43], and dinuclear gold catalytic systems [44] with no silver co-catalysts allowed the dimerization of a broad range of 2-ethynylaniline substrates under mild conditions. Interestingly, as shown in Scheme 7, Equations (2) and (3), by carrying out the reaction on the substrates **1g,h**, the side-products 7-azabicyclo[4.2.0]octa-1(6),2,4-trien-8-ol derivatives **11g,h** were isolated as a consequence of the intramolecular aminocyclization. Their formation, albeit in a low yield, seems to be promising for further research towards the synthesis of challenging 4-membered cyclic hemiaminals **11**.

**Scheme 8:**
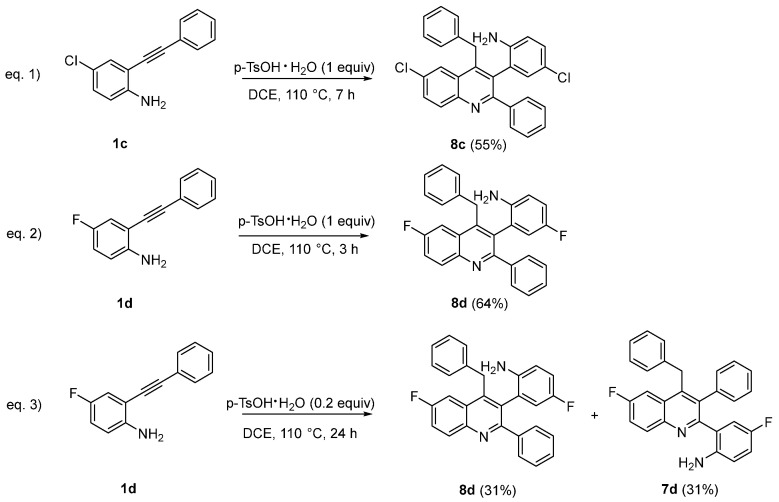
Synthesis of 2-(2-aminophenyl)quinoline derivatives **8** in DCE at 110 °C and influence of the amount of *p*-TsOH on the regioselectivity.

Finally, according to the data reported in Table 1, we proceeded with the synthesis of representative regioisomeric structures **8** by switching to DCE as the solvent (Scheme 8). Nicely, in DCE at 110 °C, the use of 1 equivalent of *p*-TsOH acid resulted in reverse regioselectivity, yielding quinoline derivatives **8c** and **8d** in satisfactory 55% and 64% yields, respectively (Scheme 8, Equations (1) and (2)). These results are in line with those observed with the Rh (III)-catalysed dimerization under anaerobic conditions in hexafluoroisopropanol [45]. Notably, as demonstrated in Scheme 8, Equation (3), in our procedure, the amount of *p*-TsOH·H_2_O resulted in being essential to controlling the regioisomeric outcome.

## 3. Materials and Methods

### Chemistry—General Part

Flash chromatography was carried out using silica gel 60 (70–230 mesh, Merck, Darmstadt, Germany). Yields are given for isolated products showing one spot on a TLC plate, and no impurities were detectable in the NMR spectrum. ^1^H NMR spectra were recorded at 400.13 MHz on a Bruker Avance III spectrometer using the standard Bruker “zg30” sequence. Chemical shifts (in ppm) were referenced to TMS (δ = 0.00 ppm), CDCl_3_ (δ = 7.26 ppm), or DMSO (δ = 2.49 ppm) as an internal standard. ^13^C NMR spectra were taken on the same machine at 100.613 MHz, using the standard Bruker “zgpg30” proton-decupled sequence. Carbon spectra were calibrated with TMS (δ = 0.0 ppm) or CDCl_3_ (δ = 77.0 ppm) as an internal standard. ^19^F-NMR spectra were taken on the same machine at 376.498 MHz using the standard Bruker “zgcigqn” proton-decupled sequence and calibrated with trifluoroacetic acid (δ = −76.55 ppm) with the substitution method. Coupling constants (J) are quoted in Hertz. Mass measurements were performed using a MALDI-TOF spectrometer AB SCIEX TOF/TOF 5800 (SCIEX, Framingham, MA, USA) using a matrix in combination with KI for the ionisation.

## 4. Experimental Procedures and Compounds Characterization

### Procedure for the Synthesis of 2-Alkynylanilines ***1a***, ***1c-h***

*Representative procedure: preparation of the 2-alkynylanilines* **1a** [46]. A 50 mL round-bottomed flask containing a magnetic stir bar was charged with 2-iodoaniline (1.900 g, 8.674 mmol, 1.0 equiv) and 5 mL of piperidine. The resulting solution was added with Pd (PPh_3_)_4_ (100.00 mg, 0.0870 mmol, 0.01 equiv), followed by CuI (16.0 mg, 0.87 mmol, 0.01 equiv). After degassing, phenylacetylene (1.14 mL, 1.060 g, 10.440 mmol, 1.2 equiv) was added to the reaction mixture. The resulting mixture was allowed to stir at room temperature under N_2_ for 5 h. Upon completion, the mixture was diluted by the addition of EtOAc and aqueous HCl (0.1 M). The separated aqueous phase was extracted with EtOAc, dried over anhydrous Na_2_SO_4_, filtered, and concentrated under reduced pressure to give the crude material, which was purified by silica gel column chromatography eluting with n-hexane/EtOAc 99/1 to provide *2-(phenylethynyl)aniline* (**1a**) (1.404 g, 85% yield). White solid. ^1^H NMR (400 MHz, CDCl_3_) *δ:* 7.53–7.52 (m, 2H), 7.37–7.33 (m, 4H), 7.16–7.12 (m, 1H), 6.73–6.71 (m, 2H), 4.27 (br, 2H) ppm.

*Preparation of the 2-(p-Tolylethynyl)aniline* (**1b**) [46]. A 50-mL round-bottomed flask containing a magnetic stir bar was charged with 2-iodoaniline (2.240 g, 10.2 mmol, 1.0 equiv), 7.1 mL of Et_3_N, and 3 mL of DMF. The resulting reaction mixture was then added with Pd (PPh_3_)_4_ (294.5 mg, 0.255 mmol, 0.025 equiv) and CuI (96.9 mg, 0.510 mmol, 0.050 equiv). The heterogeneous mixture was degassed by passing through a steady stream of nitrogen before the addition of ethynyltrimethylsilane (2.20 mL, 1.500 g, 15.300 mmol, 1.5 equiv) via a syringe. The reaction mixture was allowed to stir at room temperature under nitrogen overnight (18–20 h). Upon completion, the mixture was diluted by the addition of aqueous HCl (0.1 M). The mixture was extracted with EtOAc, and the combined organic phases were dried over anhydrous Na_2_SO_4_, filtered, and concentrated under reduced pressure to give the crude material. The crude product was taken up in 10 mL of MeOH, followed by the addition of K_2_CO_3_ (282.0 mg, 2.040 mmol, 0.2 equiv). The resulting mixture was stirred at room temperature for 2 h. Then, the reaction mixture was diluted with aqueous HCl (0.1 M) and extracted with CHCl_3_. The combined organic phases were dried over anhydrous Na_2_SO_4_, filtered, and concentrated in vacuo to afford the crude product, which was purified by silica gel column chromatography eluting hexane/EtOAc 99/1 to provide 2-ethynylaniline (734.0 mg, 62% over 2 steps).

A 25-mL round-bottomed flask containing a magnetic stir bar was charged with 4-iodotoluene (0.125 g, 1.06 mmol, 1.2 equiv) and 2 mL of piperidine. The resulting solution was added with Pd (PPh_3_)_4_ (20.0 mg, 0.088 mmol, 0.01 equiv), followed by CuI (4.0 mg, 0.088 mmol, 0.01 equiv). The mixture was degassed by passing through steady nitrogen. After degassing, 2-ethynylaniline (191.8 mg, 0.88 mmol, 1.0 equiv) was added to the reaction mixture. The resulting mixture was allowed to stir at room temperature under nitrogen (5 h). Upon completion, the mixture was diluted by the addition of aqueous HCl (0.1 M). Then the mixture was extracted with CHCl_3_, and the combined organic phases were dried over anhydrous Na_2_SO_4_, filtered, and concentrated under reduced pressure to give crude material, which was purified by silica gel column chromatography eluting hexane/EtOAc to provide *2-(p-Tolylethynyl)aniline* (**1b**) (163.8 mg, 90%). Yellow solid. ^1^H NMR (400 MHz, CDCl_3_) *δ:* 7.41–7.39 (m, 2H), 7.35–7.33 (m, 1H), 7.13–7.08 (m, 3H), 6.71–6.66 (m, 2H), 4.22 (br, 2H), 2.33 (s, 3H) ppm.

*4-Chloro-2-(phenylethynyl)aniline* (**1c**) [46]. (415.1 mg, 95%). White solid. ^1^H-NMR (400 MHz, CDCl3) *δ*: 7.50–7.49 (m, 2H), 7.35–7.32 (m, 4H), 7.06 (dd, *J* = 8.6, 1.7 Hz, 1H), 6.60 (d, *J* = 8.6 Hz, 1H), 4.24 (br, 2H) ppm.

*4-Fluoro-2-(phenylethynyl)aniline* (**1d**) [46]. (1.565 g 85%). Brown solid. ^1^H-NMR (400 MHz, CDCl_3_) *δ*: 7.52–7.51 (m, 2H), 7.36–7.34 (m, 3H), 7.06 (d, *J* = 8.9 Hz, 1H), 6.85 (t, *J* = 8.9 Hz, 1H), 6.61 (dd, *J* = 8.7, 4.7 Hz, 1H), 4.14 (br, 2H) ppm.

*2-(phenylethynyl)-4-(trifluoromethyl)aniline* (**1e**) [46]. (743 mg, 72%). Brown solid. ^1^H NMR (400 MHz, CDCl_3_) δ: 7.63–7.62 (m, 1H), 7.53–7.51 (m, 2H), 7.36–7.33 (m, 4H), 6.73 (d, *J* = 8.5 Hz, 1H), 4.56 (br, 2H) ppm.

*2-(oct-1-yn-1-yl)aniline* (**1f**) [47]. (1.858 g, ≥ 99%). Oil. ^1^H-NMR (400 MHz, CDCl_3_) δ: 7.22 (d, *J* = 7.4 Hz, 1H), 7.03 (t, *J* = 7.6 Hz, 1H), 6.64–6.60 (m, 2H), 4.11 (br, 2H), 2.43 (t, *J* = 7.1 Hz, 2H) 1.63–1.55 (m, 2H), 1.50–1.40 (m, 2H), 1.37–1.26 (m, 4H), 0.89 (t, *J* = 6.9 Hz, 3H) ppm.

*2-((Trimethylsilyl)ethynyl)aniline* (**1g**) [46]. (3.50 g, 94%). Oil. ^1^H-NMR (400 MHz, CDCl_3_) *δ:* 7.26 (d, *J* = 7.7 Hz, 1H), 7.06 (t, *J* = 7.7 Hz, 1H), 6.63–6.59 (m, 2H), 4.18 (br, 2H), 0.25 (s, 9H) ppm.

*4-Chloro-2-((trimethylsilyl)ethynyl)aniline* (**1h**) [48]. (1.271 g, 70%). Oil. ^1^H-NMR (400 MHz, CDCl_3_) δ: 7.24 (d, *J* = 2.3 Hz, 1H), 7.02 (dd, *J* = 8.7, 2.3 Hz, 1H), 6.55 (d, *J* = 8.7 Hz, 1H), 4.21 (s, 2H), 0.25 (s, 9H) ppm.

*The typical procedure for the cycloisomerization reaction of 2-alkynylanilines* **1** *to the corresponding 2-substituted indoles* **5**: *p-TsOH·H_2_O catalysed the cycloisomerization of* **1a** *to the 2-phenyl indole* **5a**. To a small vial was added 2-phenylaniline **1a** (100 mg, 0.52 mmol), *p*-TsOH·H_2_O (19 mg, 0.10 mmol), and 1,2-DCE (2 mL). The reaction mixture was stirred at 40 °C for 24 h. Then, the mixture was diluted with a saturated solution of NaHCO_3_ and extracted with CHCl_3_. The combined organic phases were dried over anhydrous Na_2_SO_4_, filtered, and concentrated under reduced pressure to give crude material, which was purified by silica gel column chromatography eluting with hexane/EtOAc 99/1 to provide the 2-phenyl-1*H*-indole (**5a**) [49]. (54 mg, 54%). White solid. ^1^H-NMR (400 MHz, CDCl_3_) δ: 8.27 (br, 1H, NH), 7.64–7.61 (m, 3H), 7.42 (t, *J* = 7.6 Hz, 2H), 7.37 (d, *J* = 8.1 Hz, 1H), 7.31 (t, *J* = 7.3 Hz, 1H), 7.19 (t, *J* = 7.5 Hz, 1H), 7.12 (t, *J* = 7.4 Hz, 1H), 6.81 (d, *J* = 1.9 Hz, 1H) ppm.

*2-(p-tolyl)-1H-indole* (**5b**) [49]. (37 mg, 90% yield). White solid. ^1^H-NMR (400 MHz, CDCl_3_) δ: 8.28 (br, 1H), 7.60 (dt, *J* = 7.8, 1.0 Hz, 1H), 7.56–7.54 (m, 2H), 7.38 (d, *J* = 8.1 Hz, 1H), 7.25–7.23 (m, 2H), 7.17 (td, *J* = 7.6, 1.3 Hz, 1H), 7.10 (td, *J* = 7.5, 1.2 Hz, 1H), 6.77 (s, 1H), 2.38 (s, 3H) ppm.

*5-chloro-2-phenyl-1H-indole* (**5c**) [49]. (30 mg, 65% yield). White solid. ^1^H-NMR (400 MHz, CDCl_3_) δ: 8.36 (br, 1H, NH), 7.66–7.63 (m, 2H), 7.58 (d, *J* = 1.9 Hz, 1H), 7.47–7.43 (m, 2H), 7.34 (tt, *J* = 7.4, 1.2 Hz, 1H), 7.13 (dt, *J* = 8.6, 0.7 Hz, 1H), 7.25 (s, 1H), 7.14 (dd, *J* = 8.6, 2.0 Hz, 1H), 6.76 (dd, *J* = 2.2, 0.9 Hz, 1H) ppm.

*5-fluoro-2-phenyl-1H-indole* (**5d**) [49]. (22 mg, 51% yield). White solid. ^1^H-NMR (400 MHz, CDCl_3_) δ: 8.29 (br, 1H, NH), 7.64 (d, *J* = 8.1 Hz, 2H), 7.44 (t, *J* = 7.6 Hz, 2H), 7.35–7.24 (m, 3H), 6.93 (td, *J* = 9.1, 2.3 Hz, 1H), 6.77 (d, *J* = 0.8 Hz, 1H) ppm.

*2-phenyl-5-(trifluoromethyl)-1H-indole* (**5e**) [50]. (26 mg, 16% yield). White solid. ^1^H-NMR (CDCl_3_, 400 MHz) δ: 8.50 (br, 1H), 7.91 (s, 1H), 7.66–7.64 (m, 2H), 7.47–7.42 (m, 4H), 7.40–7.34 (m, 1H), 6.87 (d, *J* = 1.3 Hz, 1H) ppm.

*2-hexyl-1H-indole* (**5f**) [50]. (13 mg, 7% yield). White solid. ^1^H-NMR (400 MHz, CDCl_3_) δ: 7.86 (br, 1H), 7.51 (d, *J* = 7.2 Hz, 1H), 7.30 (d, *J* = 7.9 Hz, 1H), 7.14 (t, *J* = 7.0 Hz, 1H), 7.09 (t, *J* = 7.3 Hz, 1H), 6.20 (s, 1H), 2.76 (t, *J* = 7.7 Hz, 2H), 1.75–1.67 (m, 2H), 1.40–1.24 (m, 6H), 0.88 (t, *J* = 7.1 Hz, 3H) ppm.

*The typical procedure for the p-TsOH·H_2_O promoted hydration of 2-alkynylaniline 1: synthesis of 1-(2-aminophenyl)-2-phenylethan-1-one* **6a**. To a small vial was added 2-phenylaniline **1a** (96 mg, 0.49 mmol), *p*-TsOH·H_2_O (93 mg, 0.49 mmol), 4-toluidine (268 mg, 2.5 mmol) and ethanol (2 mL). The reaction mixture was stirred at 110 °C for 20 h. Then, the mixture was diluted with a saturated solution of NaHCO_3_ and extracted with CHCl_3_. The combined organic phases were dried over anhydrous Na_2_SO_4_, filtered and concentrated under reduced pressure to give crude material which was purified by silica gel column chromatography eluting with hexane/EtOAc 95/5 to provide the *1-(2-aminophenyl)-2-phenylethan-1-one* (**6a**) [51]. (62 mg, 60% yield). Oil. ^1^H-NMR (400 MHz, CDCl_3_) δ: 7.82 (d, *J* = 8.0 Hz, 1H), 7.34–7.30 (m, 2H), 7.25–7.24 (m, 4H), 6.64–6.60 (m, 2H), 6.28 (br, 2H), 4.24 (s, 2H) ppm.

*1-(2-aminophenyl)-2-(p-tolyl)ethan-1-one* (**6b**) [22] (38 mg, 26% yield). White solid. ^1^H-NMR (400 MHz, CDCl_3_) δ: 7.83 (d, *J* = 8.2 Hz, 1H), 7.24 (t, *J* = 7.6 Hz, 1H), 7.14–7.12 (m, 4H), 6.65–6.61 (m, 2H), 6.27 (br, 2H), 4.21 (s, 2H), 2.32 (s, 3H) ppm.

*1-(2-amino-5-chlorophenyl)-2-phenylethan-1-one* (**6c**) [52]. (25 mg, 27% yield). White solid. ^1^H-NMR (400 MHz, CDCl_3_) δ: 7.79 (d, *J* = 2.3 Hz, 1H), 7.36–7.32 (m, 2H), 7.28–7.26 (m, 1H), 7.24–7.22 (m, 2H), 7.19 (dd, *J* = 8.8, 2.4 Hz, 1H), 6.58 (d, *J* = 8.9 Hz, 1H), 6.28 (br, 2H), 4.21 (s, 2H) ppm.

*1-(2-amino-5-fluorophenyl)-2-phenylethan-1-one* (**6d**). (30 mg, 17% yield). Oil. ^1^H-NMR (400 MHz, CDCl_3_) δ: 7.49 (dd, *J* = 9.9, 2.9 Hz, 1H), 7.34–7.31 (m, 2H), 7.27–7.22 (m, 3H), 7.03 (ddd, *J* = 9.0, 7.7, 2.9 Hz, 1H), 6.64 (dd, *J* = 9.1, 4.6 Hz, 1H), 5.75 (br, 2H), 4.19 (s, 2 H) ppm; ^19^F{^1^H} NMR (376 MHz, CDCl_3_) δ: −127.64 (s, 1F) ppm; ^13^C {^1^H} NMR (150 MHz, CDCl_3_) δ: 199.1 (d, *J* = 2.8 Hz, Cq), 153.6 (d, *J* = 235.3 Hz, Cq), 146.8 (Cq), 134.8 (Cq), 129.4 (2CH), 128.7 (2CH), 126.9 (CH), 122.6 (d, *J* = 23.5 Hz, CH), 118.9 (d, *J* = 7.0 Hz, CH), 117.2 (d, *J* = 5.3 Hz, Cq), 116.1 (d, *J* = 22.2 Hz, CH), 46.2 (CH_2_) ppm; HRMS: *m*/*z* (MALDI-TOF) positive ion, calculated for C_14_H_12_FKNO: [M + K]^+^ 268.0540, Found: 268.0538.

*1-(2-amino-5-trifluoromethyl)phenyl)-2-phenylethan-1-one* (**6e**). (51 mg, 50% yield). Oil. ^1^H-NMR (400 MHz, CDCl_3_) δ: 8.10 (s, 1H), 7.43 (dd, *J* = 8.7, 1.9 Hz, 1 H), 7.36–7.32 (m, 2H), 7.28–7.23 (m, 3H), 6.68 (d, *J* = 8.7 Hz, 1H), 6.61 (br, 2H), 4.27 (s, 2H) ppm; ^19^F{^1^H} NMR (376 MHz, CDCl_3_) δ: −127.64 (s, 3F) ppm; ^13^C {^1^H} NMR (150 MHz, CDCl_3_) δ: 119.5 (Cq), 152.9 (Cq), 134.6 (Cq), 130.7 (q, *J* = 3.2 Hz, CH), 129.5 (2CH), 129.2 (q, *J* = 4.1 Hz, CH), 128.8 (2CH), 127.1 (CH), 124.3 (q, *J* = 270.4 Hz, CF_3_), 117.68 (CH), 117.67 (q, *J* = 33.3 Hz, Cq), 116.2 (Cq), 46.1 (CH_2_) ppm; HRMS: *m*/*z* (MALDI-TOF) positive ion, calculated for C_15_H_12_F_3_KNO: [M + K]^+^ 318.0508, Found: 318.0510.

*1-(2-aminophenyl)octan-1-one* (**6f**) [53]. (98 mg, 56% yield). Oil. ^1^H NMR (400 MHz, CDCl_3_) δ: 7.72 (d, *J* = 8.3 Hz, 1H), 7.22 (t, *J* = 7.6 Hz, 1H), 6.63–6.60 (m, 2H), 6.26 (br, 2H), 2.90 (t, *J* = 7.5 Hz, 2H), 1.72–1.67 (m, 2H), 1.35–1.29 (m, 8H), 0.88 (t, *J* = 6.6 Hz, 3H) ppm.

*1-(2-aminophenyl)ethan-1-one* (**6g**) [Commercial Product]. (96 mg, 66% yield); ^1^H NMR (400 MHz, CDCl_3_) δ: 7.70 (dt, *J* = 8.0, 1.5 Hz, 1H), 7.29–7.24 (m, 1H), 6.69–6.64 (m, 2H), 6.23 (br, 2H), 2.56 (s, 3H) ppm.

*1-(2-amino-5-chlorophenyl)ethan-1-one* (**6h**) [Commercial Product]. ^1^H NMR (400 MHz, CDCl_3_) δ: 7.65 (d, *J* = 2.7 Hz, 1H), 7.19 (m, 1H), 6.59 (d, *J* = 8.8 Hz, 1H), 6.27 (s, 2H), 2.54 (m, 3H) ppm.

*The typical procedure for the regioselective p-TsOH·H_2_O promoted dimerization reaction of 2-alhynylaniline* **1** *to quinolines* **7**. To a small vial was added 2-phenylaniline **1a** (150 mg, 0.78 mmol), *p*-TsOH·H_2_O (148 mg, 0.78 mmol),) and ethanol (2 mL). The reaction mixture was stirred at 110 °C for 20 h. Then, the mixture was diluted with a saturated solution of NaHCO_3_ and extracted with CHCl_3_. The combined organic phases were dried over anhydrous Na_2_SO_4_, filtered and concentrated under reduced pressure to give crude material which was purified by silica gel column chromatography eluting with hexane/EtOAc 90/10 to provide the *2-(4-benzyl-3-phenylquinolin-2-yl)aniline* (**7a**) [40] and *2-(4-Benzyl-2-phenylquinolin-3-yl)aniline* (**8a**) [41] ratio 1:0.25 calculated by ^1^H NMR (104 mg, 70% yield). Oil. ^1^H-NMR (400 MHz, CDCl_3_) δ: 8.23 (d, *J* = 8.4 Hz, 1H, 8a), 8.16 (d, *J* = 8.4 Hz, 1H, 7a), 8.01 (d, *J* = 8.4 Hz, 1H, 8a), 7.93 (d, *J* = 8.4 Hz, 1H, 7a), 7.72–7.66 (m, 1H, 7a + 1H, 8a), 7.51–7.45 (m, 1H, 7a + 3H, 8a), 7.22–7.08 (m, 8H, 7a + 6H, 8a), 7.03–6.92 (m, 3H, 7a + 3H, 8a), 6.80 (dt, *J* = 7.6, 1.3 Hz, 1H, 8a), 6.71 (dt, *J* = 7.7 Hz, 1.4 Hz, 1H, 7a), 6.67 (dt, *J* = 8.1 Hz, 1.1 Hz, 1H, 7a), 6.60–6.55 (m, 2H, 8a), 6.41 (tt, *J* = 7.5 Hz, 1.1 Hz, 1H, 7a), 4.45 (d, *J* = 15.6 Hz, 1H, AB system 8a), 4.39 (s, 2H, 7a), 4.34 (br, 2H, 7a), 4.29 (d, *J* = 15.6 Hz, 1H, AB system 8a), 3.29 (br, 2H, 8a) ppm.

*2-(4-(4-methylbenzyl)-3-(p-tolyl)quinolin-2-yl)aniline* (**7b**) and *2-(4-(4-methylbenzyl)-2-(p-tolyl)quinolin-3-yl)aniline* (**8b**) [45] ratio 1:0.40 calculated by ^1^H NMR (65 mg, 50% yield). Oil. ^1^H-NMR (400 MHz, CDCl_3_) δ 8.21 (d, *J* = 8.4 Hz, 1H, 8b), 8.14 (d, *J* = 8.4 Hz, 1H, 7b), 7.97 (d, *J* = 8.4 Hz, 1H, 8b), 7.90 (d, *J* = 8.5 Hz, 1H, 7b), 7.70 (t, *J* = 7.1 Hz, 1H, 8b), 7.65 (dd, *J* = 8.2, 7.1 Hz, 1H, 7b), 7.48 (t, *J* = 7.2 Hz, 1H, 8b), 7.43 (t, *J* = 7.1 Hz, 1H, 7b), 7.38 (d, *J* = 7.9 Hz, 2H 8b), 7.04–6.88 (m, 7H, 7b + 5H, 8b), 6.83–6.81 (m, 3H, 8b), 6.74 (d, *J* = 7.7 Hz, 1H, 7b), 6.66 (d, *J* = 8.0 Hz, 1H, 7b), 6.62–6.55 (m, 2H, 8b), 6.43 (t, *J* = 7.5 Hz, 1H, 7b), 4.39 (d, *J* = 15.57 Hz, 1H, 8b, AB system), 4.34 (s, 2H, 7b), 4.33 (br, 2H, 7b), 4.22 (d, *J* = 15.57 Hz, 1H 8b, AB system), 3.29 (br, 2H, 8b), 2.27 (s, 3H, 7b), 2.26 (s, 3H, 8b), 2.23 (s, 3H, 8b), 2.23 (s, 3H, 7b) ppm; ^13^C {^1^H} NMR (150 MHz, CDCl_3_) δ: 159.59 (8b), 158.78 (7b), 147.86 (8b), 146.94 (7b), 145.90 (8b), 144.85 (7b), 144.72 (7b), 144.10 (8b), 138.10 (8b), 137.60 (8b), 137.14 (7b), 136.63 (8b), 135.95 (7b), 135.44 (7b), 135.40 (8b), 135.17 (7b), 131.40 (8b), 131.18 (7b), 131.10 (8b), 130.27 (8b), 129.90 (7b), 129.73 (7b), 129.19 (8b), 129.16 (7b), 129.14 (8b), 129.01 (7b), 128.99 (8b), 128.75 (8b), 128.49 (7b), 128.45 (7b), 128.30 (8b), 128.19 (8b), 128.07 (7b), 127.97 (7b), 126.81 (8b), 126.80 (7b), 126.70 (7b), 126.48 (8b), 126.31 (8b), 125.30 (7b), 125.18 (7b), 124.02 (8b), 118.37 (8b), 117.60 (7b), 116.28 (7b), 115.35 (8b), 35.22 (7b), 34.71 (8b), 21.22 (8b), 21.16 (7b), 20.95 (7b), 20.93 (8b) ppm; HRMS: *m*/*z* (MALDI-TOF) positive ion, calculated for C_30_H_26_N_2_Na: [M + Na]^+^ 437.1994, Found: 437.1998.

*2-(4-Benzyl-6-chloro-3-phenylquinolin-2-yl)-4-chloroaniline* (**7c**) and *2-(4-Benzyl-6-chloro-2-phenylquinolin-3-yl)-4-chloroaniline* (**8c**) ratio 1:0.25 calculated by ^1^H NMR (43 mg, 50% yield). Oil. Eluent: hexane/ethyl acetate (90:10). ^1^H-NMR (400 MHz, CDCl_3_) δ: 8.16 (dd, *J* = 8.9, 0.6 Hz, 1H, 8c), 8.07 (dd, *J* = 9.0, 0.6 Hz, 1H, 7c), 8.04 (d, *J* = 2.2 Hz, 1H, 8c), 7.92 (d, *J* = 2.1 Hz, 1H, 7c), 7.68 (ddd, *J* = 9.0, 2.4, 1.1 Hz, 1H, 8c), 7.64 (ddd, *J* = 8.8, 2.2, 1.1 Hz, 1H, 7c), 7.44 (dd, *J* = 7.8, 2.0 Hz, 2H, 8c), 7.25–7.15 (m, 8H, 7c, 6H, 8c), 6.96 (d, *J* = 7.6 Hz, 2H, 7c), 7.01 (dd, *J* = 8.6, 2.2 Hz, 1H, 8c), 6.82 (d, J1 = 8.9 Hz, 1H, 7c), 6.89 (d, *J* = 6.2 Hz, 2H, 8c), 6.72 (d, *J* = 2.2 Hz, 1H, 8c), 6.70 (d, *J* = 2.3 Hz, 1H, 7c), 6.59 (d, *J* = 8.6 Hz, 1H, 7c), 6.49 (d, *J* = 8.4 Hz, 1H, 8c), 4.40 (d, *J* = 15.8 Hz, 1H, 8c, AB system), 4.34 (s, 2H, 7c), 4.20 (d, *J* = 15.8 Hz, 1H, 8c, AB system), 4.31 (br, 2H, 7c), 3.25 (br, 2H, 8c) ppm.

*2-(4-(4-Fluorobenzyl)-3-(4-fluorophenyl)quinolin-2-yl)aniline* (**7d**) and *2-(4-Benzyl-6-fluoro-2-phenylquinolin-3-yl)-4-fluoroaniline* (**8d**) ratio 1:0.40 calculated by ^1^H NMR. (105 mg, 65% yield). Oil. Eluent: hexane/ethyl acetate (90:10). ^1^H NMR (400 MHz, CDCl_3_) δ: 8.22 (dd, *J* = 9.2, 5.6 Hz, 1H, 8d), 8.14 (dd, *J* = 9.2, 5.6 Hz, 1H, 7d), 7.61 (dd, *J* = 10.2, 2.8 Hz, 1H, 8d), 7.53 (dd, *J* = 10.3, 2.7 Hz, 1H, 7d), 7.49 (ddd, *J* = 9.2, 8.0, 2.8 Hz, 1H, 8d), 7.48–7.43 (m, 3H 7d + 2H 8d), 7.24–7.08 (m, 6H 7d, 6H 8d), 6.99 (d, *J* = 1.7 Hz, 1H, 7d), 6.97 (d, *J* = 1.0 Hz, 1H, 7d), 6.90 (d, *J* = 1.8 Hz, 1H, 8d), 6.83 (d, *J* = 1.1 Hz, 1H, 8d), 6.77 (td, *J* = 8.5, 2.9 Hz, 1H, 8d), 6.68 (td, *J* = 8.5, 2.9 Hz, 1H, 7d), 6.59 (dd, *J* = 8.78, 4.82 Hz, 1H, 7d), 6.51 (dd, *J* = 8.8, 1.4 Hz, 1H, 8d), 6.50 (d, *J* = 8.8 Hz, 1H, 8d), 6.47 (dd, *J* = 9.53, 2.98 Hz, 1H, 7d), 4.36 (d, *J* = 15.72 Hz, 1H, AB system, 8d), 4.33 (s, 2H, 7d), 4.23 (d, *J* = 15.72 Hz, 1H, AB system, 8d), 4.14 (br, 2H, 7d), 3.16 (br, 2H, 8d) ppm; ^19^F{^1^H} NMR (376 MHz, CDCl_3_) δ: −111.32 (s, 1F, 7d), −111.63 (s, 1F, 8d), −126.39 (s, 1F, 8d) −127.68 (s, 1F, 7d) ppm; ^13^C {^1^H} NMR (150 MHz, CDCl_3_) δ: 160.9 (d, *J* = 248.4 Hz, Cq, 7d), 160.8 (d, *J* = 248.3 Hz, Cq, 8d), 158.8 (d, *J* = 2.8 Hz, Cq, 8d), 156.8 (dd, *J* = 2.7, 2.03 Hz, Cq, 7d), 155.8 (d, *J* = 237.4 Hz, Cq, 8d), 155.5 (d, *J* = 235.9 Hz, Cq, 7d), 145.5 (d, *J* = 5.6 Hz, Cq, 8d), 145.1 (Cq, 8d), 144.3 (d, *J* = 5.7 Hz, Cq, 7d), 144.2 (Cq, 7d), 141.0 (d, *J* = 2.1 Hz, 1 Cq, 7d), 140.4 (d, *J* = 2.0 Hz, Cq, 8d), 140.3 (Cq, 8d), 139.3 (Cq, 7d), 138.8 (Cq, 8d), 137.4 (Cq, 7d), 136.5 (d, *J* = 0.5 Hz, Cq, 7d), 132.9 (d, *J* = 9.3 Hz, CH, 8d), 132.3 (d, *J* = 9.3 Hz, CH, 7d), 130.9 (d, *J* = 1.0 Hz, Cq, 8d), 129.8 (2CH, 7d), 129.1 (2CH, 8d), 128.7 (2CH, 7d), 128.6 (2CH, 8d), 128.2 (CH, 8d), 128.1 (2CH, 8d), 128.04 (2CH, 7d), 128.01 (2CH, 7d), 127.9 (d, *J* = 9.5 Hz, Cq, 7d), 127.8 (2CH, 8d), 127.71 (d *J* = 8.9 Hz, Cq, 8d), 127.70 (d *J* = 12.7 Hz, CH, 7d), 126.8 (d, *J* = 7.13 Hz, Cq, 7d), 126.4 (CH, 8d), 126.3 (CH, 7d), 124.6 (d, *J* = 7.4 Hz, Cq, 8d), 119.9 (d, *J* = 25.8 Hz, CH, 8d), 119.6 (d, *J* = 25.9 Hz, CH, 7d), 117.4 (d, *J* = 22.5 Hz, CH, 8d), 117.3 (d, *J* = 12.1 Hz, CH, 7d), 117.1 (d, *J* = 3.7 Hz, CH, 7d), 116.5 (d, *J* = 7.8 Hz, CH, 8d), 115.8 (d, *J* = 22.4 Hz, CH, 8d), 115.5 (d, *J* = 22.4 Hz, CH, 7d), 108.9 (d, *J* = 22.8 Hz, CH, 7d), 108.7 (d, *J* = 22.9 Hz, CH, 8d), 35.71 (CH_2_, 7d), 35.2 (CH_2_, 8d) ppm; HRMS: *m*/*z* (MALDI-TOF) positive ion, calculated for C_28_H_20_F_2_KN_2_: [M + K]^+^ 461.1232, Found: 461.1231.

*2-(4-(4-(Trifluoromethyl)benzyl)-3-(4-(trifluoromethyl)phenyl)quinolin-2-yl)aniline* (**7e**) (21 mg, 20% yield). Oil. Eluent: hexane/ethyl acetate (90:10). ^1^H NMR (400 MHz, CDCl_3_) δ: 8.30 (s, 1H), 8.24 (d, *J* = 9.1 Hz, 1H), 7.89 (d, *J* = 8.2 Hz, 1H), 7.34–7.18 (m, 7H), 7.06–7.04 (m, 2 H), 6.97–6.95 (m, 3 H), 6.72 (d, *J* = 8.7 Hz, 1H), 4.79 (br, 2H), 4.44 (s, 2H) ppm; ^19^F {^1^H} NMR (376 MHz, CDCl_3_) δ: −61.58 (s, 3F), −62.46 (s, 3F) ppm; HRMS: *m*/*z* (MALDI-TOF) positive ion, calculated for C_30_H_20_F_6_KN_2_: [M + K]^+^ 561.1168, Found: 561.1170.

*2-(3-hexyl-4-octylquinolin-2-yl)aniline* (**7f**) (32 mg, 18% yield). Oil. Eluent: hexane/ethyl acetate (90:10). ^1^H NMR (400 MHz, CDCl_3_): δ 8.00 (d, *J* = 8.3 Hz, 1H), 7.67 (t, *J* = 7.6 Hz, 1H), 7.50 (t, *J* = 7.6 Hz, 1H), 7.23 (t, *J* = 8.6 Hz, 1H), 6.99 (d, *J* = 7.3 Hz, 1H), 6.85 (t, *J* = 7.5 Hz, 1H), 6.80 (d, *J* = 8.1 Hz, 1H), 3.38 (br, 2H), 2.93 (td, *J* = 12.1, 4.89 Hz, 1H), 2.75–2.60 (m, 3H), 1.69–1.43 (m, 3H), 1.29–1.16 (m, 15H), 0.84 (t, *J* = 6.8 Hz, 3H), 0.81 (t, *J* = 7.1 Hz, 3H) ppm; ^13^C {^1^H} NMR (150 MHz, CDCl_3_): δ 162.5 (Cq),148.0 (Cq), 147.7 (Cq), 143.8 (Cq), 130.8 (CH), 130.3 (Cq), 129.7 (CH), 128.9 (CH), 128.8 (CH), 126.1 (Cq), 125.6 (CH), 124.1 (CH), 123.7 (Cq), 118.4 (CH), 115.2 (CH), 37.1 (CH_2_), 31.6 (CH_2_), 31.5 (CH_2_), 30.5 (CH_2_), 30.0 (CH_2_), 29.5 (CH_2_), 29.4 (CH_2_), 29.3 (CH_2_), 28.7 (CH_2_), 22.6 (CH_2_), 22.5 (CH_2_), 14.1 (CH_3_), 14.0 (CH_3_) ppm; HRMS: *m*/*z* (MALDI-TOF) positive ion, calculated for C_28_H_38_N_2_Na: [M + Na]^+^ 425.2933, Found: 425.2930.

*2-(4-Methylquinolin-2-yl)aniline* (**7g**) [45]. Eluent: petroleum ether/ethyl acetate (20:1). (25.6 mg, 73%). Yellow solid. ^1^H NMR (400 MHz, CDCl_3_) δ: 8.02 (d, *J* = 8.5 Hz, 1H), 7.92 (d, *J* = 8.3 Hz, 1H), 7.64–7.62 (m, 2H), 7.47 (t, *J* = 7.6 Hz, 1H), 7.22–7.15 (m, 1H), 6.80–6.75 (m, 1H), 6.61–6.57 (m, 2H), 6.18 (br, 2H), 2.67 (s, 3H) ppm.

*4-chloro-2-(6-chloro-4-methylquinolin-2-yl)aniline* (**7h**) [44]. Eluent: petroleum ether/ethyl acetate (20:1). (28 mg, 16%). Yellow solid. ^1^H NMR (400 MHz, CDCl_3_) δ: 7.93 (d, *J* = 7.2 Hz, 1H), 7.92 (s, 1H), 7.62–7.59 (m, 3H), 7.13 (dd, *J* = 8.6, 2.4 Hz, 1H), 6.70 (d, *J* = 8.6 Hz, 1H), 6.15 (br, 2H), 2.68 (d, *J* = 0.9 Hz, 3H) ppm.

*The typical procedure for the regioselective p-TsOH·H_2_O promoted the dimerization reaction of 2-alhynylaniline* **1** *to quinolines* **8**. To a small vial was added 2-phenylaniline 1a (101 mg, 0.52 mmol), *p*-TsOH·H_2_O (99 mg, 0.52 mmol), and DCE (2 mL). The reaction mixture was stirred at 110 °C for 4 h. Then, the mixture was diluted with a saturated solution of NaHCO_3_ and extracted with CHCl_3_. The combined organic phases were dried over anhydrous Na_2_SO_4_, filtered and concentrated under reduced pressure to give crude material which was purified by silica gel column chromatography eluting with hexane/EtOAc 90/10 to provide *2-(4-Benzyl-2-phenylquinolin-3-yl)aniline* (**8a**) (48 mg, 48% yield). Unseparable mixture 8a:7a Ratio 9:2 calculated by ^1^H NMR.

*2-(4-Benzyl-6-chloro-2-phenylquinolin-3-yl)-4-chloroaniline* (**8c**) (45 mg, 55% yield). Oil. hexane/EtOAc 90/10. Unseparable mixture **8c**:**7c** Ratio 9:1 calculated by ^1^H NMR.

*2-(4-Benzyl-6-fluoro-2-phenylquinolin-3-yl)-4-fluoroaniline* (**8d**) (64 mg, 64% yield) hexane/EtOAc 90/10. **Oil.** ^1^H NMR (400 MHz, CDCl_3_) δ: 8.25 (dd, *J* = 9.2, 5.6 Hz, 1H), 7.60 (dd, *J* = 10.2, 2.8 Hz, 1H), 7.49 (ddd, *J* = 9.2, 8.0, 2.8 Hz, 1H), 7.44–7.43 (m, 2H), 7.24–7.20 (m, 3H), 7.18–7.12 (m, 3H), 6.90 (d, *J* = 1.8 Hz, 1H), 6.83 (d, *J* = 1.1 Hz, 1H), 6.77 (td, *J* = 8.5, 2.9 Hz, 1H), 6.51 (dd, *J* = 8.8, 1.4 Hz, 1H), 6.50 (d, *J* = 8.8 Hz, 1H), 4.36 (d, *J* = 15.72 Hz, 1H, AB system), 4.23 (d, *J* = 15.72 Hz, 1H, AB system), 3.16 (br, 2H) ppm; ^19^F{^1^H} NMR (376 MHz, CDCl_3_) δ: −111.41 (s, 1F), −126.38 (s, 1F) ppm; ^13^C{^1^H} NMR (150 MHz, CDCl_3_) δ: 160.8 (d, *J* = 248.3 Hz, Cq), 158.8 (d, *J* = 2.8 Hz, Cq), 155.8 (d, *J* = 237.4 Hz, Cq), 145.5 (d, *J* = 5.6 Hz, Cq), 145.1 (Cq), 140.4 (d, ^4^*J* = 2.0 Hz, Cq), 140.3 (Cq), 138.8 (Cq), 132.9 (d, *J* = 9.3 Hz, CH), 130.9 (d, *J* = 1.0 Hz, Cq), 129.1 (2CH), 128.6 (2CH), 128.2 (CH), 128.1 (2CH), 127.8 (2CH), 127.7 (d *J* = 8.9 Hz, Cq), 126.4 (CH), 124.6 (d, *J* = 7.4 Hz, Cq), 119.9 (d, *J* = 25.8 Hz, CH), 117.4 (d, *J* = 22.5 Hz, CH), 116.5 (d, *J* = 7.8 Hz, CH), 115.8 (d, *J* = 22.4 Hz, CH), 108.7 (d, *J* = 22.9 Hz, CH), 35.2 (CH_2_) ppm; HRMS: *m*/*z* (MALDI-TOF) positive ion, calculated for C_28_H_20_F_2_KN_2_: [M + K]^+^ 461.1232, Found: 461.1235.

*Synthesis of the 3-phenylquinolin-4(1*H*)-one* **9a** [54]. To a small vial was added 2-phenylaniline **1a** (72 mg, 0.37 mmol), *p*-TsOH·H_2_O (71 mg, 0.37 mmol), and DMF (2 mL). The reaction mixture was stirred at 110 °C for 18 h. Then, the mixture was diluted with a saturated solution of NaHCO_3_ and extracted with CHCl_3_. The combined organic phases were dried over anhydrous Na_2_SO_4_, filtered and concentrated under reduced pressure to give crude material which was purified by silica gel column chromatography eluting with hexane/EtOAc 70/30 to provide 3-phenylquinolin-4(1*H*)-one **9a** (41 mg, 33% yield). Yellow solid. ^1^H NMR (400 MHz, DMSO) δ: 12.04 (s, 1H), 8.20 (d, *J* = 8.3 Hz, 1H), 8.15 (s, 1H), 7.73–7.71 (m, 2H), 7.65 (t, *J* = 7.6 Hz, 1H), 7.58 (d, *J* = 8.3 Hz, 1H), 7.40–7.32 (m, 3H), 7.27 (t, *J* = 7.2 Hz, 1H) ppm.

*Synthesis of the 2,2′-Diphenyl-1H,1′H-3,3′-biindole* **10a** [25]. To a small vial was added 2-phenylaniline **1a** (98 mg, 0.5 mmol), *p*-TsOH·H_2_O (20 mg, 0.10 mmol), dimethoxymethane (190 mg, 2.50 mmol), and DCE (2 mL). The reaction mixture was stirred at 40 °C for 4 h. Then, the mixture was diluted with a saturated solution of NaHCO_3_ and extracted with CHCl_3_. The combined organic phases were dried over anhydrous Na_2_SO_4_, filtered and concentrated under reduced pressure to give crude material which was purified by silica gel column chromatography eluting with hexane/EtOAc 90/10 to provide the 2,2′-Diphenyl-1*H*,1′*H*-3,3′-biindole (**10a**) (73 mg, 76% yield). White solid. ^1^H NMR (400 MHz, CDCl_3_) δ: 8.02 (s, 2H), 7.58–7.56 (m, 4H), 7.40 (t, *J* = 6.9 Hz, 4H), 7.34–7.19 (m, 6H), 7.07 (t, *J* = 7.2 Hz, 2H) 6.85 (t, *J* = 7.2 Hz, 2H) ppm.

*8-methyl-7-azabicyclo [4.2.0]octa-1,3,5-trien-8-ol* (**11g**). (8 mg, 8% yield). Oil. ^1^H NMR (400 MHz, CDCl_3_) δ: 7.19 (d, *J* = 7.7 Hz, 1H), 7.03 (t, *J* = 7.6 Hz, 1H), 6.80 (t, *J* = 7.5 Hz, 1H), 6.62 (d, *J* = 8.0 Hz, 1H), 4.49 (br, 1H), 1.86 (s, 3H), 1.57 (br, 1H) ppm; HRMS: *m*/*z* (MALDI-TOF) positive ion, calculated for C_8_H_9_KNO: [M + K]^+^ 174.0321, Found: 174.0317.

*3-chloro-8-methyl-7-azabicyclo [4.2.0]octa-1,3,5-trien-8-ol* (**11h**). (11 mg, 12% yield). Oil. ^1^H NMR (400 MHz, CDCl_3_) δ: 7.16 (d, *J* = 2.3 Hz, 1H), 7.01 (dd, *J* = 8.6, 2.3 Hz, 1H), 6.58 (d, *J* = 8.6 Hz, 1H), 4.48 (br, 1H), 1.83 (s, 3H), 1.54 (br, 1H) ppm; ^13^C{^1^H} NMR (150 MHz, CDCl_3_) δ: 139.1 (Cq), 129.5 (Cq), 128.4 (CH), 125.6 (CH), 125.3 (Cq), 119.2 (CH), 81.4 (Cq), 27.0 (CH_3_) ppm; HRMS: *m*/*z* (MALDI-TOF) positive ion, calculated for C_8_H_8_ClNNaO: [M + Na]^+^ 192.0195, Found: 192.0195.

*Control experiment ruling out the formation of* **7a** *from* **6a**. To a small vial was added 1-(2-aminophenyl)-2-phenylethan-1-one **6a** (87 mg, 0.78 mmol), 2-(phenylethynyl)aniline **1a** (80 mg, 0.41 mmol), *p*-TsOH·H_2_O (148 mg, 0.41 mmol), and ethanol (2 mL). The reaction mixture was stirred at 110 °C for 20 h. Then, the mixture was diluted with a saturated solution of NaHCO_3_ and extracted with CHCl_3_. The combined organic phases were dried over anhydrous Na_2_SO_4_, filtered and concentrated under reduced pressure to give crude material which was purified by silica gel column chromatography eluting with hexane/EtOAc 90/10 to provide 1-(2-aminophenyl)-2-phenylethan-1-one **6a** (95 mg; 84 mg recovered + 11 mg, 13% from **1a**), 2-phenyl-1*H*-indole **5a** (21 mg from **1a**, 24% from **1a**) and 2-(4-benzyl-3-phenylquinolin-2-yl)aniline **7a** (36 mg, 45% from **1a**).

## 5. Conclusions

In summary, we have explored the key factors that influence product selectivity in Brønsted acid-catalysed/mediated reactions of 2-alkynylanilines. The methodologies presented here provide viable alternatives to metal-catalysis approaches, often offering complementary selectivity through simple adjustments such as changing the solvent, varying the amount of the Brønsted acid promoter, and/or modifying the reaction temperature. Using essentially the same catalytic system, we established optimal conditions for selectively producing dimerization products **7** or **8**, directing the reaction towards hydration product **6**, or achieving cycloisomerization to indoles **5** and bis-indoles **10**. Mechanistic insights into the origins of chemo- and regioselectivity were obtained through quantum chemical calculations using Density Functional Theory.

## Figures and Tables

**Table 1:** Screening of the reaction conditions.

Entry ^a^	Solvent	Brønsted Acid/ (Equiv.)	T (°C)/ Time (h)	Products/ Yield (%) ^b^
1	EtOH	*p*-TsOH·H_2_O/1.0	110/24	**6a** (29) + **7a**/**8a (80**/**20)** (70)
2	EtOH	*p*-TsOH·H_2_O/0.2	110/24	**6a** (46) + **7a** (53)
3	EtOH	MsOH/1.0	110/24	**6a** (16) + **7a**/**8a** (67/33) (53)
4	EtOH	TfOH/1.0	110/24	**5a** (13) + **6a** (22) + **7a**/**8a** (44/66) (34)
5	Toluene	TfOH/1.0	110/24	**5a** (7) **+ 6a** (3) + **7a**/**8a** (43/67) (50)
6	*^i^*PrOH	*p*-TsOH·H_2_O/1.0	110/24	**5a** (5) + **6a** (12) + **7a**/**8a** (55/45) (48)
7	DCE	*p*-TsOH·H_2_O/0.2	40/24	**5a** (54) + **1a (**34%)
8	DCE	*p*-TsOH·H_2_O/1.0	40/24	**6a** (62)
9	DCE	*p*-TsOH·H_2_O/0.2	60/3	**5a** (32) + **6a** (8) + **8a** (9)
10	DCE	*p*-TsOH·H_2_O/0.2	80/3	**5a** (42) + **6a** (13) + **8a** (30)
11	DCE	*p*-TsOH·H_2_O/1.0	110/4	**6a** (17) + **7a** (19)+ **8a** (49)
12	THF	*p*-TsOH·H_2_O/1.0	60/24	**6a** (15) + **1a** (56)
13	DMF	*p*-TsOH·H_2_O/1.0	110/17	**5a** (45) + **6a** (22) + **9a** (30)
14	DCE/DMM	*p*-TsOH·H_2_O/traces	40/24	**10a** (76)

**^a^** Reactions have been carried out on a 0.37–0.78 mmol scale. **^b^** Yields refer to isolated products.

## Data Availability

The original contributions presented in the study are included in the article/supplementary material, further inquiries can be directed to the corresponding author/s.

## Supplementary Materials

The following supporting information can be downloaded at: https://www.mdpi.com/article/10.3390/molecules29153693/s1, Computational Details; Copies of ^1^H, ^19^F, ^13^C NMR Spectra; Optimized Cartesian contributes of all investigated species; Details of NCI analysis and RESP analysis.
