# Supplementary material for: Product Selectivity Control in the Brønsted Acid-Mediated Reactions with 2-Alkynylanilines
Source: Molecules. 2024 Aug 4;29(15):3693. doi: 10.3390/molecules29153693 (PMC11314341; doi:10.3390/molecules29153693)
Supplement: Supplementary file 1 [file molecules-29-03693-s001.zip › Revised SI.pdf]

Product Selectivity Control in the Brønsted Acid Mediated Reactions of 2-Alkynylanilines

Valerio Morlacci [1], Massimiliano Aschi[1], Marco Chiarini [2], Caterina Momoli[1], Laura Palombi[1]*, and Antonio Arcadi[1]**

1 *Dipartimento di Scienze Fisiche e Chimiche, Università degli studi dell'Aquila. Via Vetoio-67100 Coppito (AQ)- Italy. E-mail: antonio.arcadi@univaq.it*

2 *Dipartimento di Bioscienze e Tecnologie Agroalimentari e Ambientali, Università degli Studi di Teramo, Via R. Balzarini, 64100 Teramo – Italy*

Table of Contents

I. **Computational Details**..........S02
II. **Copies of ^{1}H, ^{19}F and ^{13}C NMR spectra**..........S03
III. **Optimized cartesian coordinates of all the investigated species**..........S27
IV. **Details of NCI analysis and RESP analysis**..........S117

I. Computational details

Quantum chemical calculations were performed in the framework of Density Functional Theory using the wB97XD functional [1] making use of a version of Grimme's D2 dispersion model in conjunction with the 6-31G* basis set. Test calculations performed on some of the critical points (see below) using a larges basis set (6-311+G*) did not produce significant variations. All the critical points were located and characterized as true minima or first order saddle points through the calculations of the molecular vibrations in harmonic approximation. Subsequently, the effect of the solvent (either ethanol or 1,2 dichloroethane) was included in the mean field approximation, using the Polarizable Continuum Model [2] as implemented in the Gaussian16 software [3] used for all these calculations. The standard molar free energy was then calculated at 110°C, for each of the involved species, using the standard statistical mechanical relations (through the harmonic frequencies and moment of inertia from DFT calculations) and considering, as reference state, 1.0 mole/litre for all the species with the exception of the solvent, for which we utilized the experimental density in the experimental conditions.

Non-covalent Interaction Index (NCI) analysis based on promolecular density was carried using Multiwfn program. This method, which allows the visualization of the non-covalent interactions in the 3D space, is based on the determination of the strength of the non-covalent interaction through the product between electron density (ρ) and the sign of the second eigenvalue of the Hessian, sign(λ). The interactions are classified inti three types:

- $\rho \cdot sign(\lambda) < 0$ strongly attractive (pictorially represented in blue in this study)

---

1 D. Chai and M. Head-Gordon, "Long-range corrected hybrid density functionals with damped atom-atom dispersion corrections," *Phys. Chem. Chem. Phys.*, **10** (2008) 6615-20.

2 J. Tomasi, B. Mennucci, and R. Cammi, "Quantum mechanical continuum solvation models," *Chem. Rev.*, **105** (2005) 2999-3093.

3 Gaussian 16, Revision C.01, M. J. Frisch, G. W. Trucks, H. B. Schlegel, G. E. Scuseria, M. A. Robb, J. R. Cheeseman, G. Scalmani, V. Barone, G. A. Petersson, H. Nakatsuji, X. Li, M. Caricato, A. V. Marenich, J. Bloino, B. G. Janesko, R. Gomperts, B. Mennucci, H. P. Hratchian, J. V. Ortiz, A. F. Izmaylov, J. L. Sonnenberg, D. Williams-Young, F. Ding, F. Lipparini, F. Egidi, J. Goings, B. Peng, A. Petrone, T. Henderson, D. Ranasinghe, V. G. Zakrzewski, J. Gao, N. Rega, G. Zheng, W. Liang, M. Hada, M. Ehara, K. Toyota, R. Fukuda, J. Hasegawa, M. Ishida, T. Nakajima, Y. Honda, O. Kitao, H. Nakai, T. Vreven, K. Throssell, J. A. Montgomery, Jr., J. E. Peralta, F. Ogliaro, M. J. Bearpark, J. J. Heyd, E. N. Brothers, K. N. Kudin, V. N. Staroverov, T. A. Keith, R. Kobayashi, J. Normand, K. Raghavachari, A. P. Rendell, J. C. Burant, S. S. Iyengar, J. Tomasi, M. Cossi, J. M. Millam, M. Klene, C. Adamo, R. Cammi, J. W. Ochterski, R. L. Martin, K. Morokuma, O. Farkas, J. B. Foresman, and D. J. Fox, Gaussian, Inc., Wallingford CT, 2016.

- $\rho \cdot \mathrm{sign}(\lambda) \sim 0$ weak van der waals interactions (pictorially represented in green in this study)

- $\rho \cdot \mathrm{sign}(\lambda) > 0$ repulsive interactions (pictorially represented in red in this study)

The same program was also used for performing atomic-charge calculations within the RESP scheme.

II. Copies of ^{1}H, ^{19}F and ^{13}C NMR spectra

1a

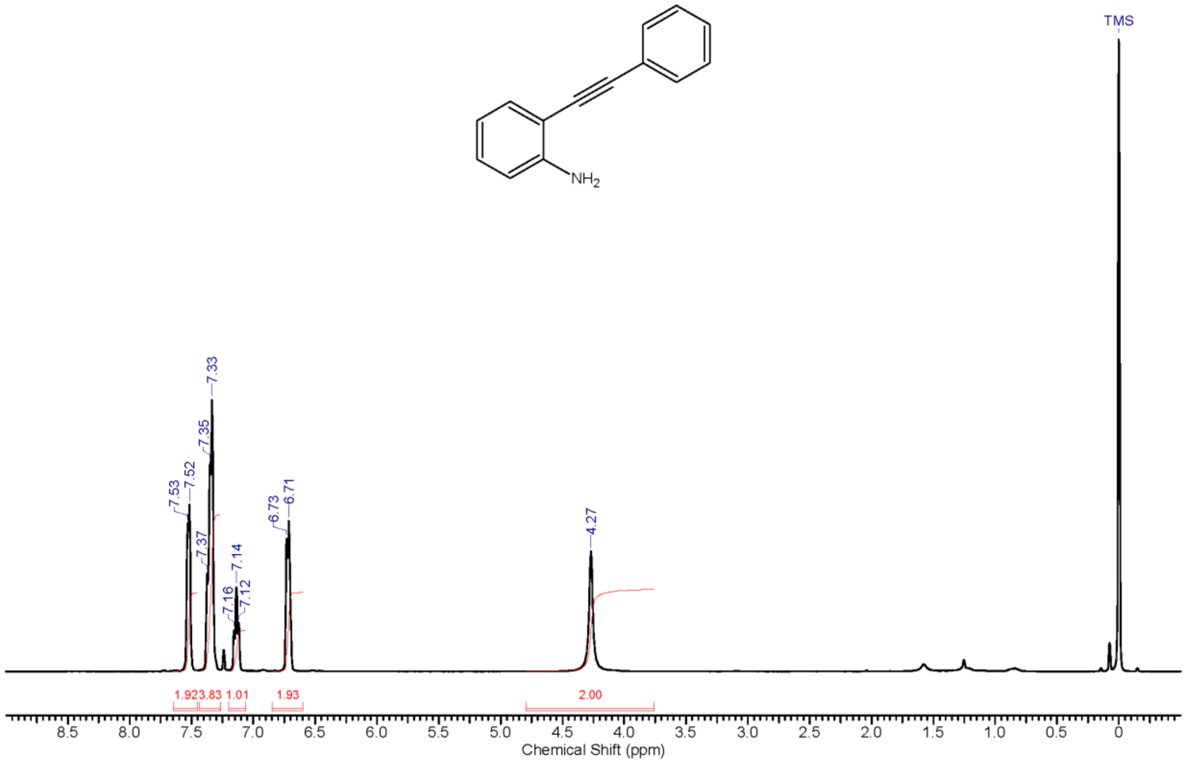

1b

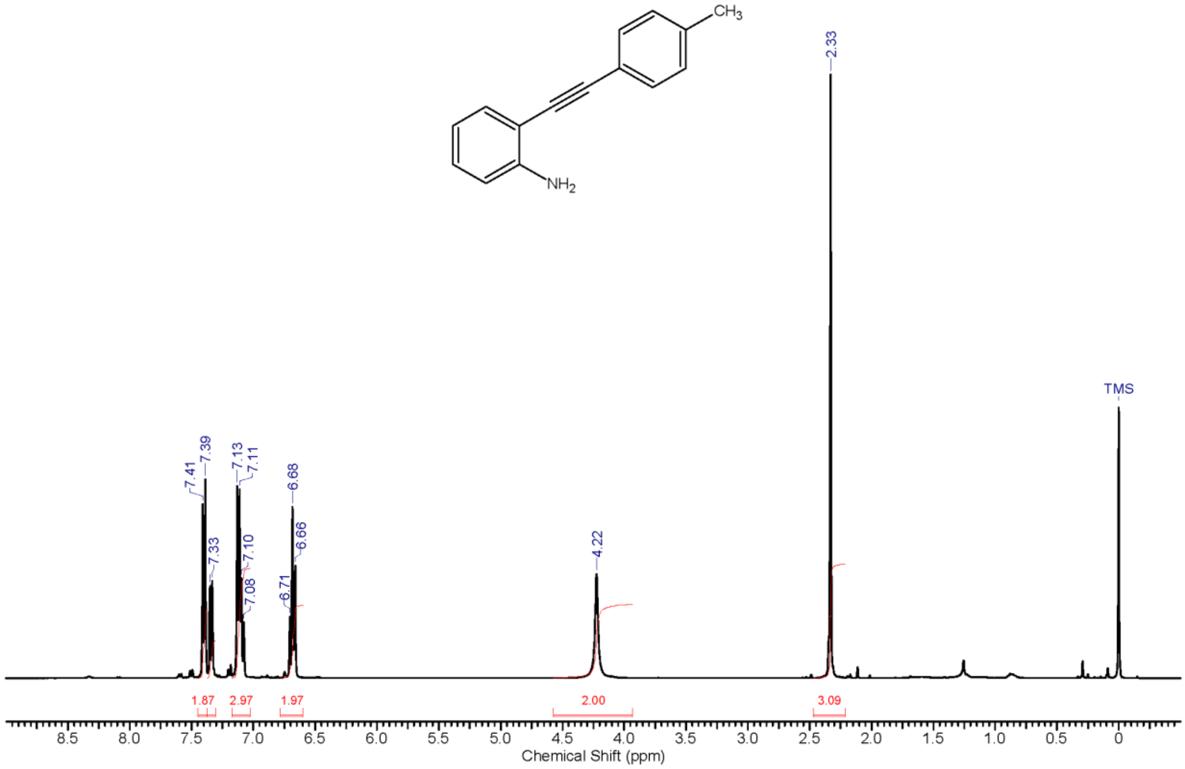

1c

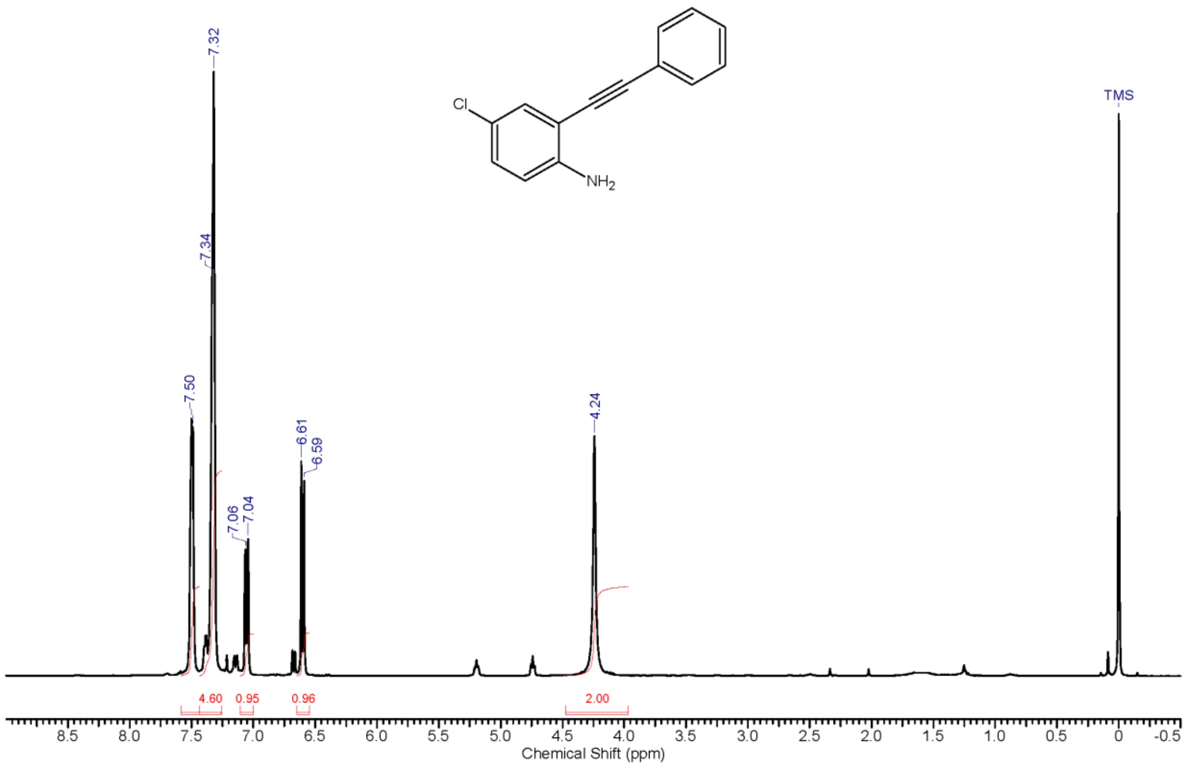
Cl
NH2
TMS
7.32
7.34
7.50
7.06
7.04
6.61
6.59
4.24
4.60
0.95
0.96
2.00
Chemical Shift (ppm)

1d

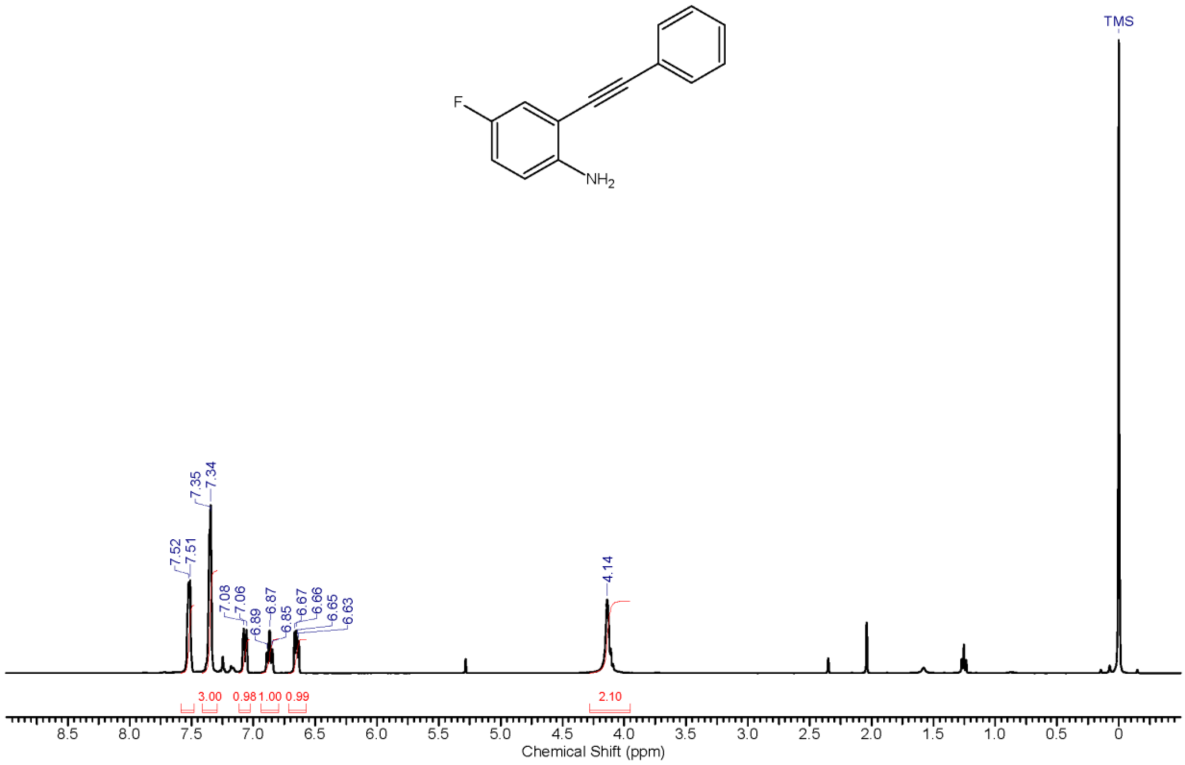
F
NH2
TMS
7.35
7.34
7.52
7.51
7.08
7.06
6.89
6.87
6.85
6.67
6.66
6.65
6.63
4.14
3.00
0.98
1.00
0.99
2.10
Chemical Shift (ppm)

1e

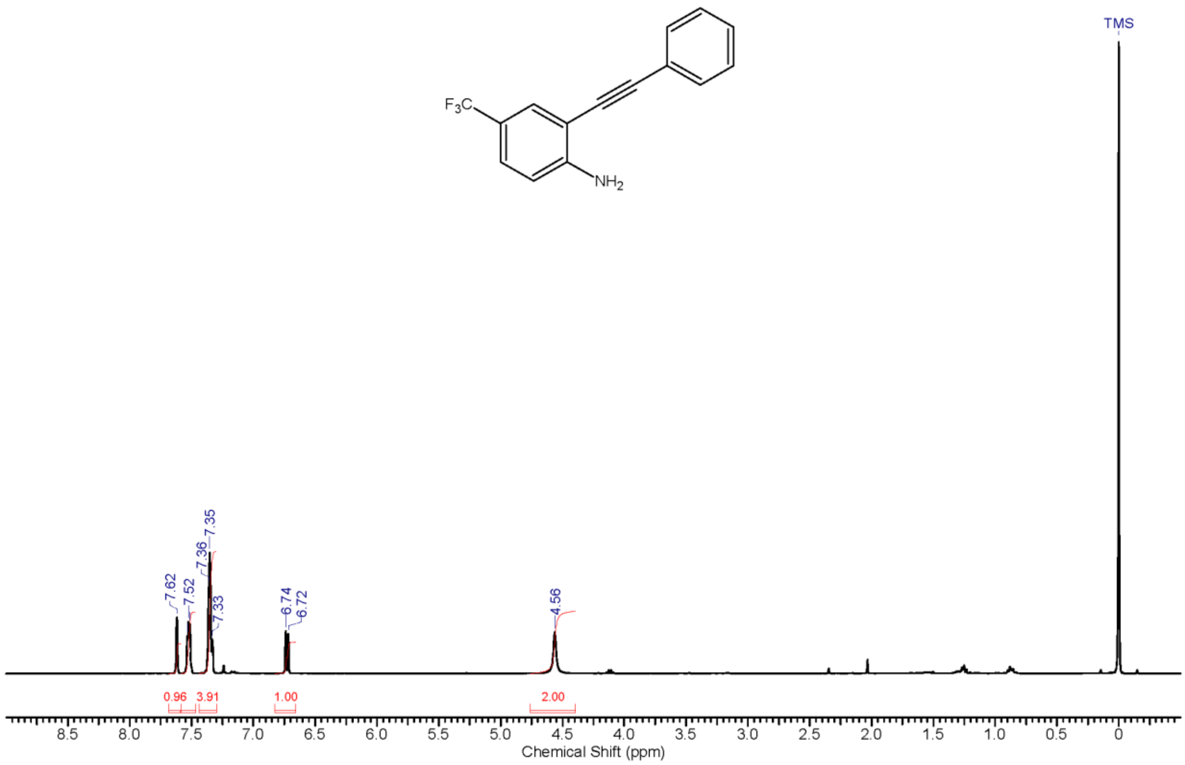
F3C
NH2
TMS
7.62
7.52
7.36
7.35
7.33
6.74
6.72
4.56
0.96
3.91
1.00
2.00
Chemical Shift (ppm)

1f

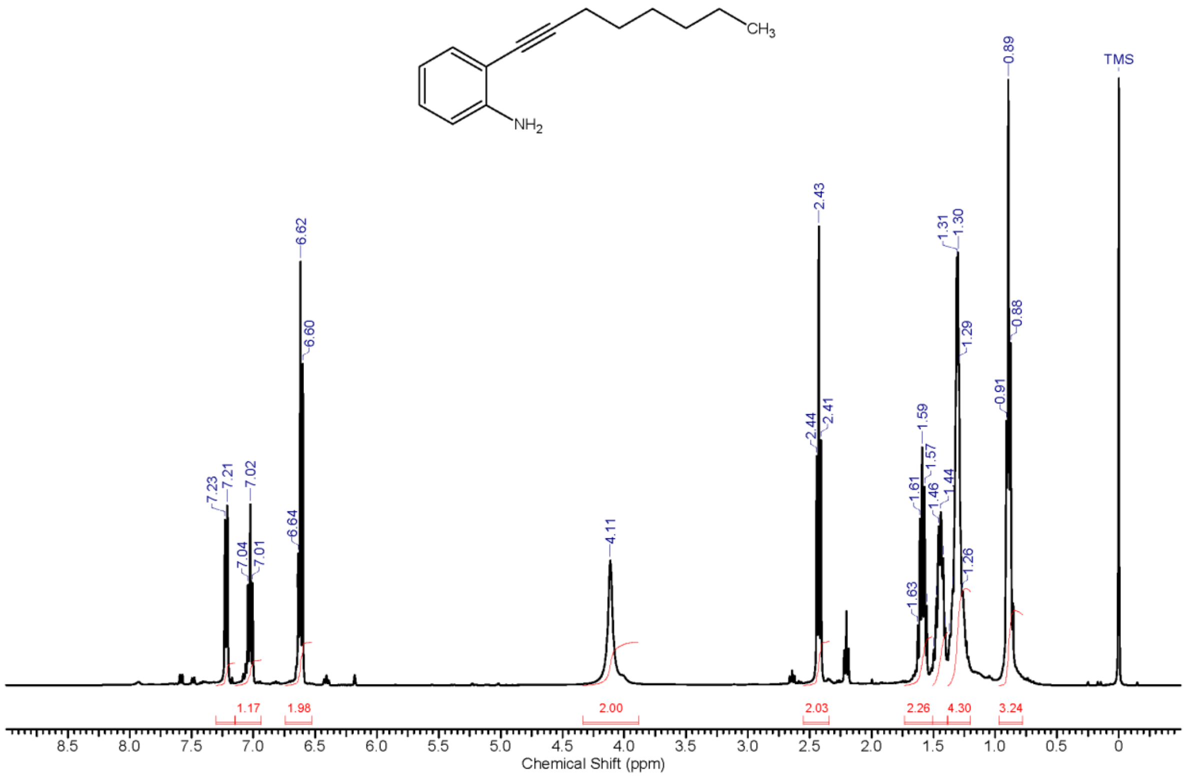
CH3
NH2
TMS
7.23
7.21
7.04
7.02
7.01
6.64
6.62
6.60
4.11
2.44
2.43
2.41
1.63
1.61
1.59
1.57
1.46
1.44
1.31
1.30
1.29
1.26
0.91
0.89
0.88
1.17
1.98
2.00
2.03
2.26
4.30
3.24
Chemical Shift (ppm)

1g

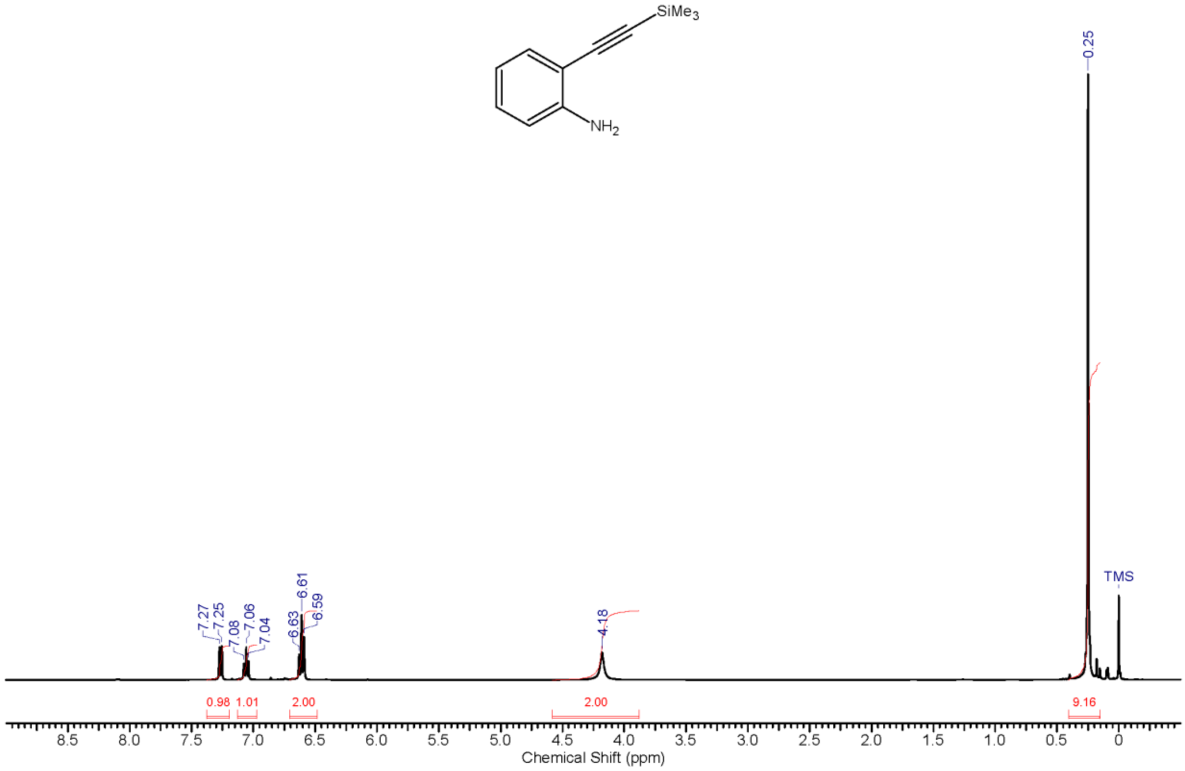
SiMe3
NH2
0.25
TMS
7.27
7.25
7.08
7.06
7.04
6.63
6.61
6.59
4.18
0.98
1.01
2.00
2.00
9.16
8.5
8.0
7.5
7.0
6.5
6.0
5.5
5.0
4.5
4.0
3.5
3.0
2.5
2.0
1.5
1.0
0.5
0
Chemical Shift (ppm)

1h

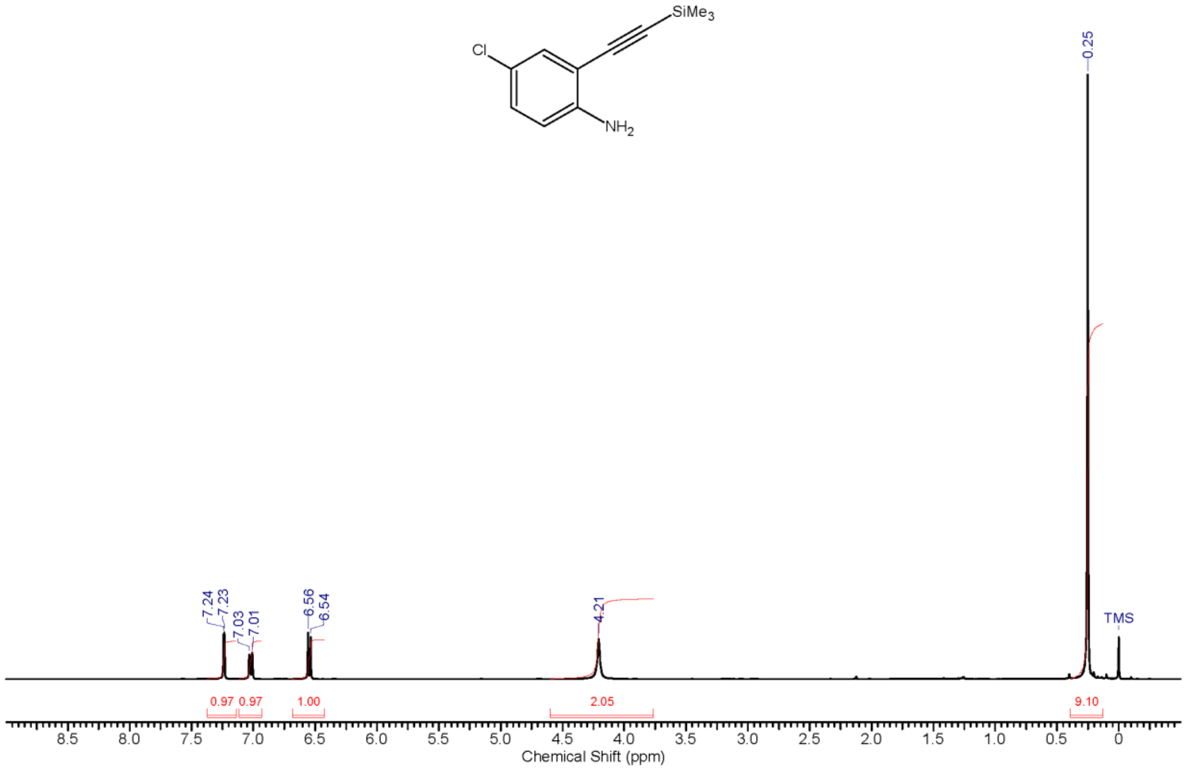
SiMe3
Cl
NH2
0.25
TMS
7.24
7.23
7.03
7.01
6.56
6.54
4.21
0.97
0.97
1.00
2.05
9.10
8.5
8.0
7.5
7.0
6.5
6.0
5.5
5.0
4.5
4.0
3.5
3.0
2.5
2.0
1.5
1.0
0.5
0
Chemical Shift (ppm)

5a

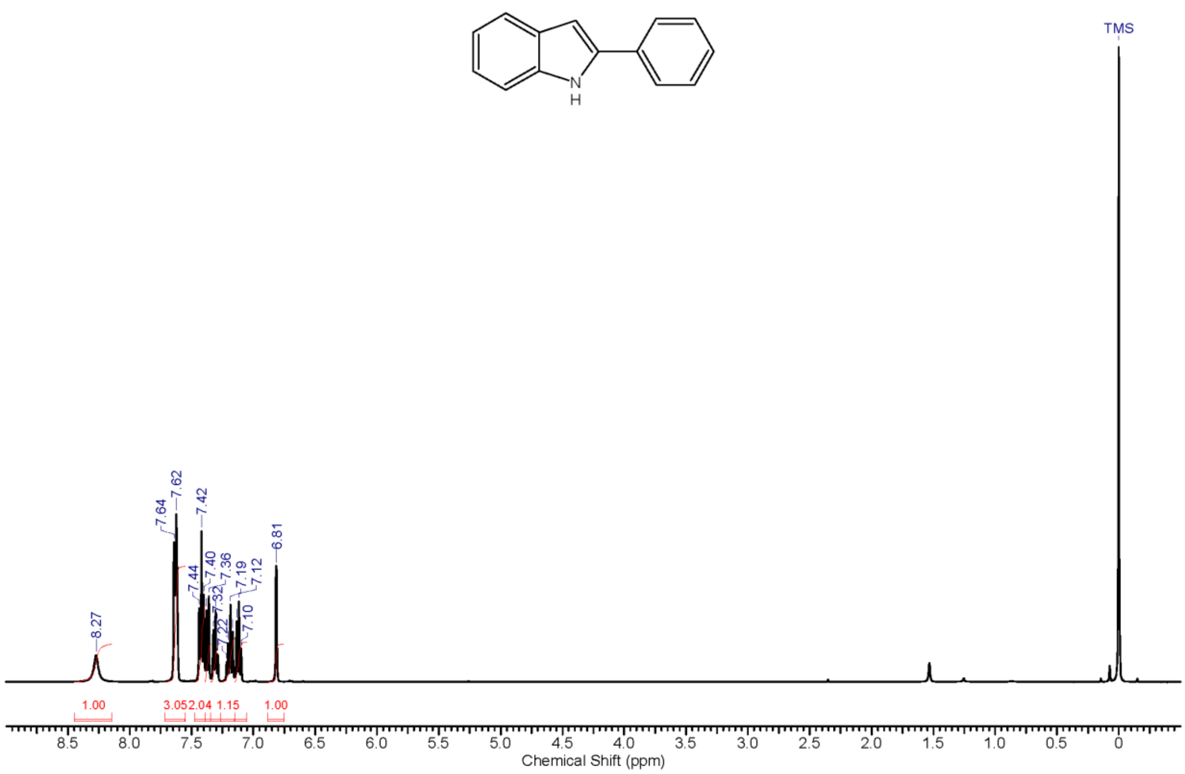

N
H
TMS
8.27
7.64
7.62
7.44
7.42
7.40
7.36
7.32
7.22
7.19
7.12
7.10
6.81
1.00
3.05
2.04
1.15
1.00
Chemical Shift (ppm)

5b

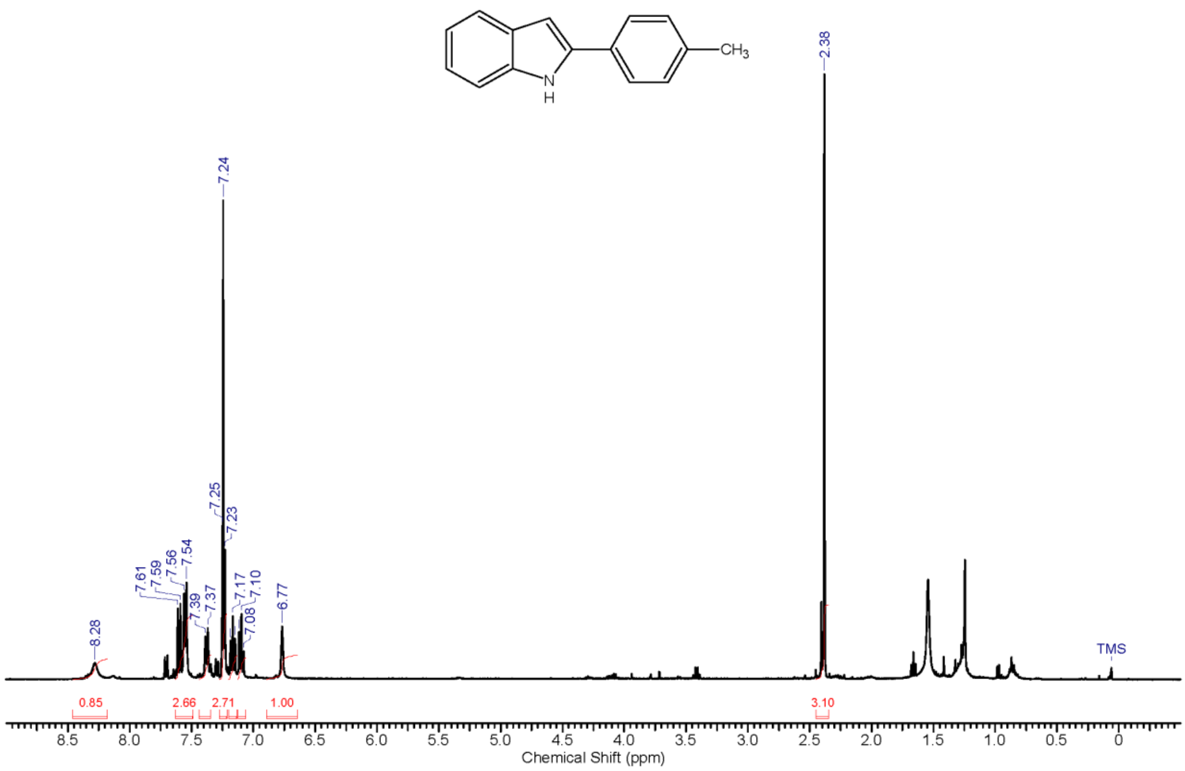

N
H
CH3
8.28
7.61
7.59
7.56
7.54
7.39
7.37
7.25
7.24
7.23
7.17
7.10
7.08
6.77
2.38
TMS
0.85
2.66
2.71
1.00
3.10
Chemical Shift (ppm)

5c

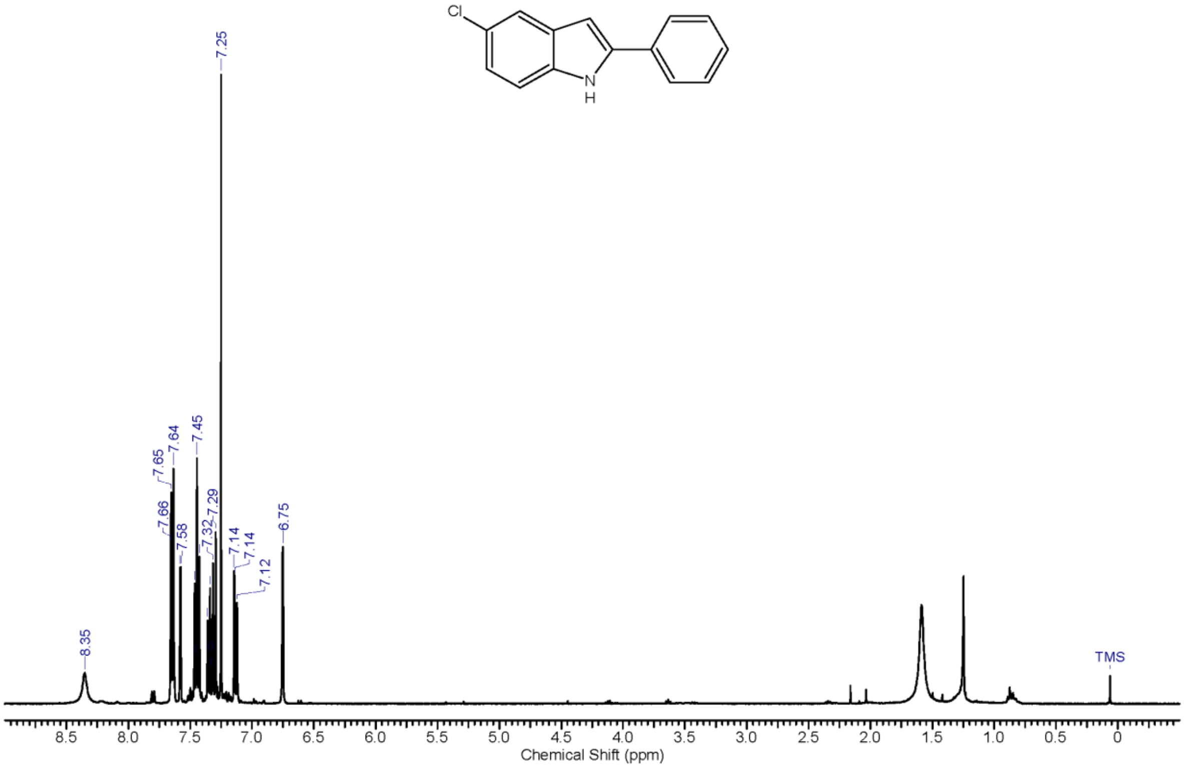
Cl
N
H
8.35
7.66
7.65
7.64
7.58
7.45
7.32
7.29
7.25
7.14
7.14
7.12
6.75
TMS
Chemical Shift (ppm)

5d

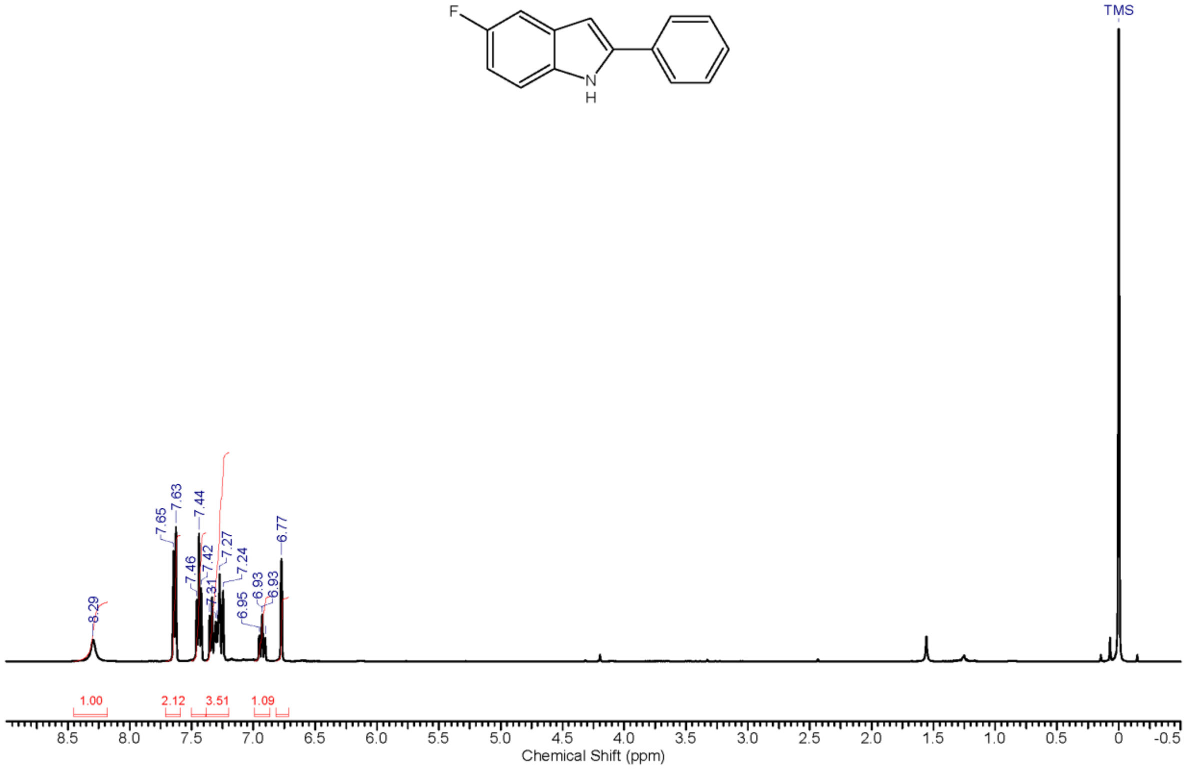
F
N
H
TMS
8.29
7.65
7.63
7.46
7.44
7.42
7.31
7.27
7.24
6.95
6.93
6.93
6.77
1.00
2.12
3.51
1.09
Chemical Shift (ppm)

5e

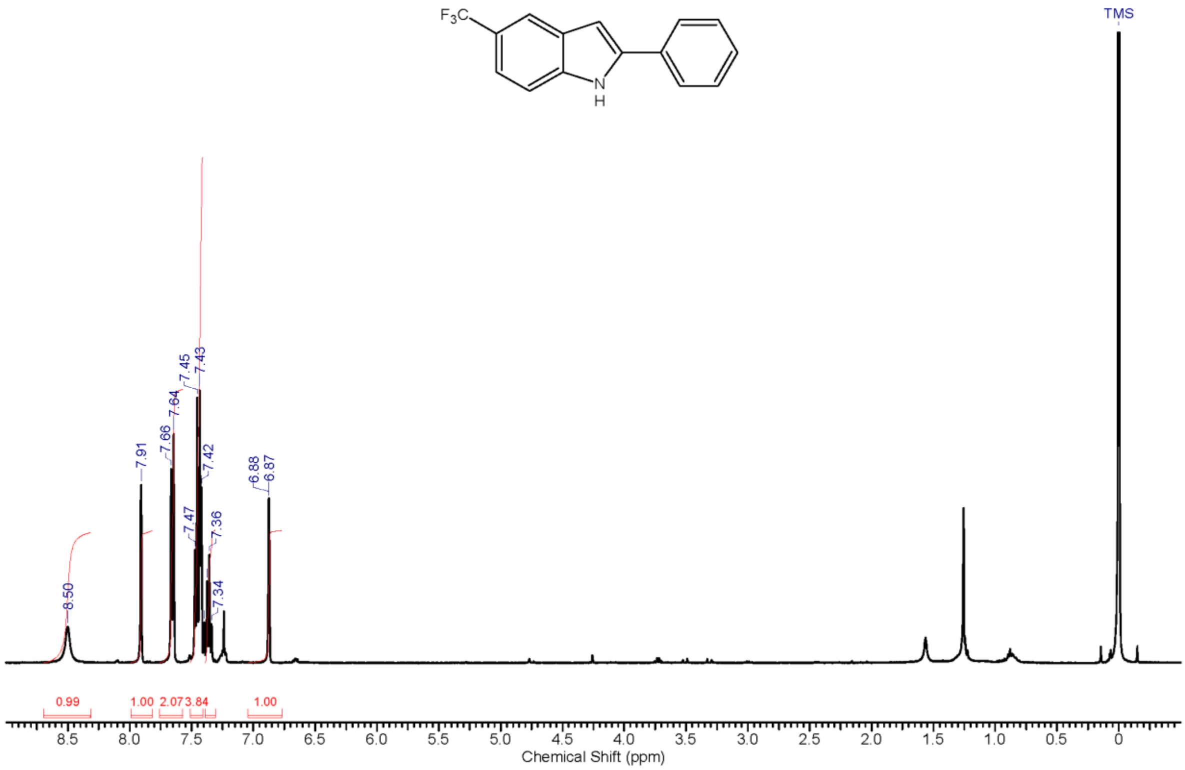

5f

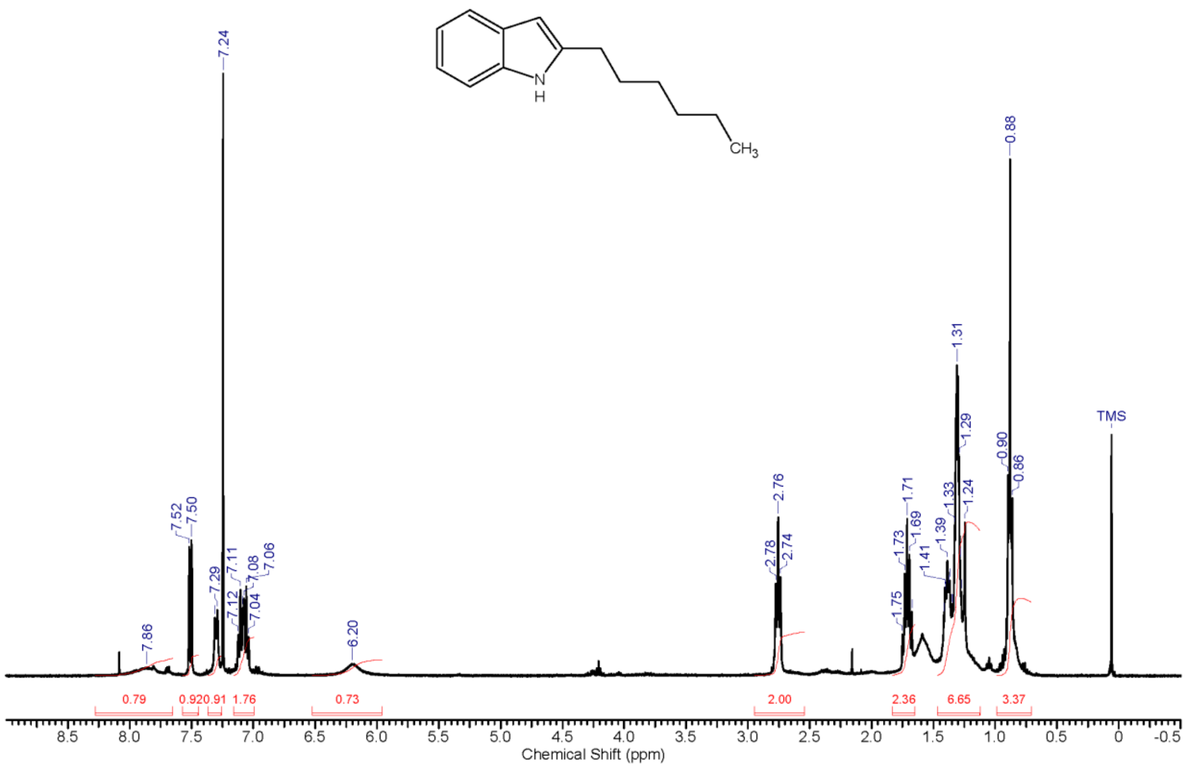

6a

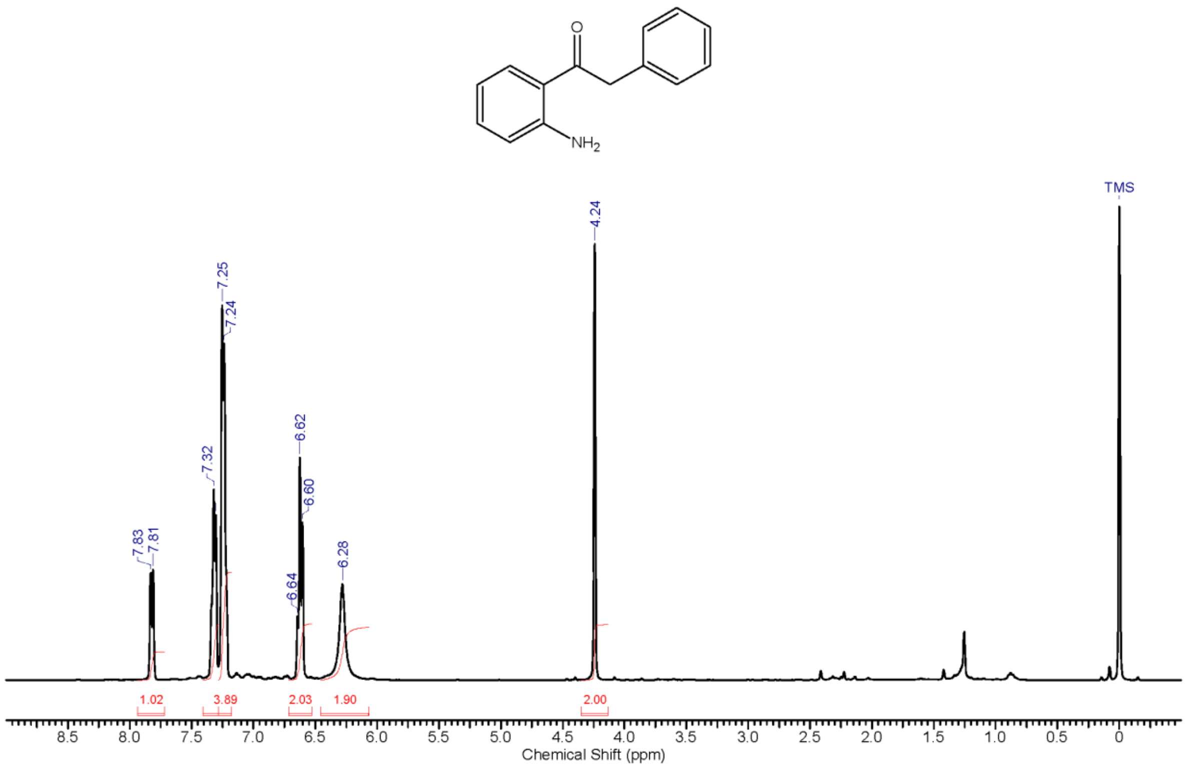
O
NH2
TMS
7.83
7.81
7.32
7.25
7.24
6.64
6.62
6.60
6.28
4.24
1.02
3.89
2.03
1.90
2.00
Chemical Shift (ppm)

6b

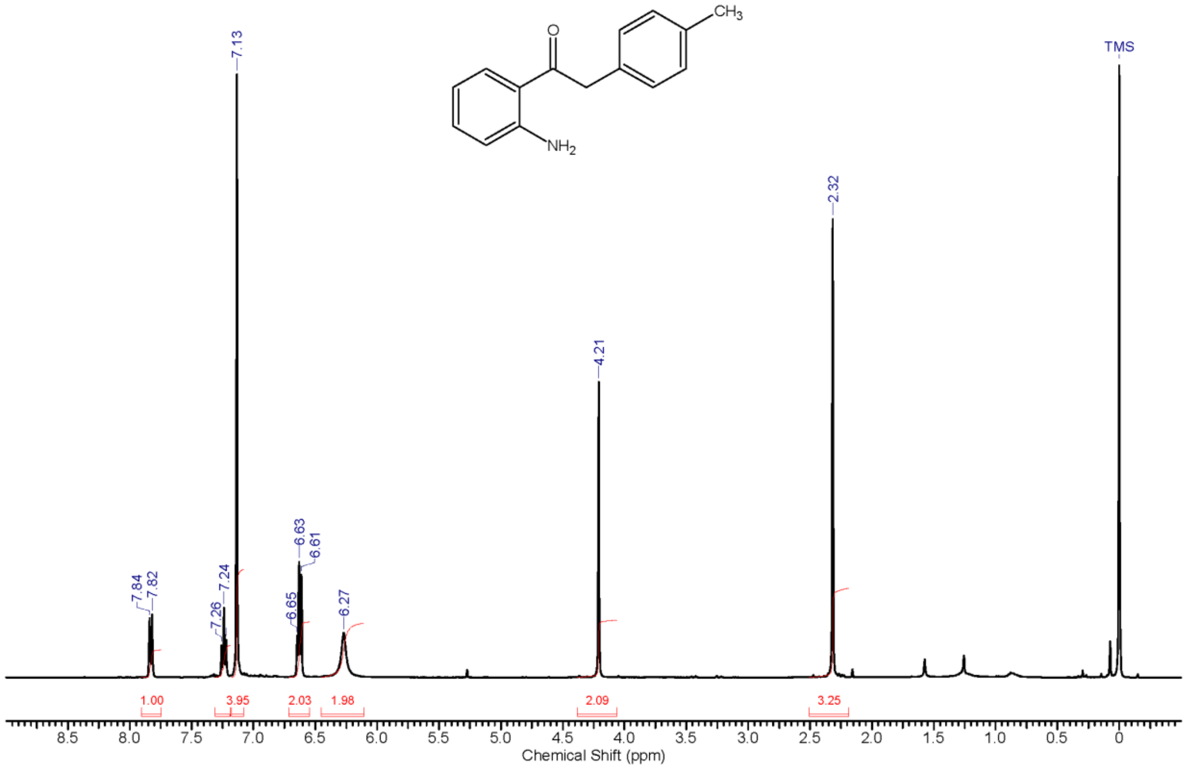
O
CH3
NH2
TMS
7.84
7.82
7.26
7.24
7.13
6.65
6.63
6.61
6.27
4.21
2.32
1.00
3.95
2.03
1.98
2.09
3.25
Chemical Shift (ppm)

6c

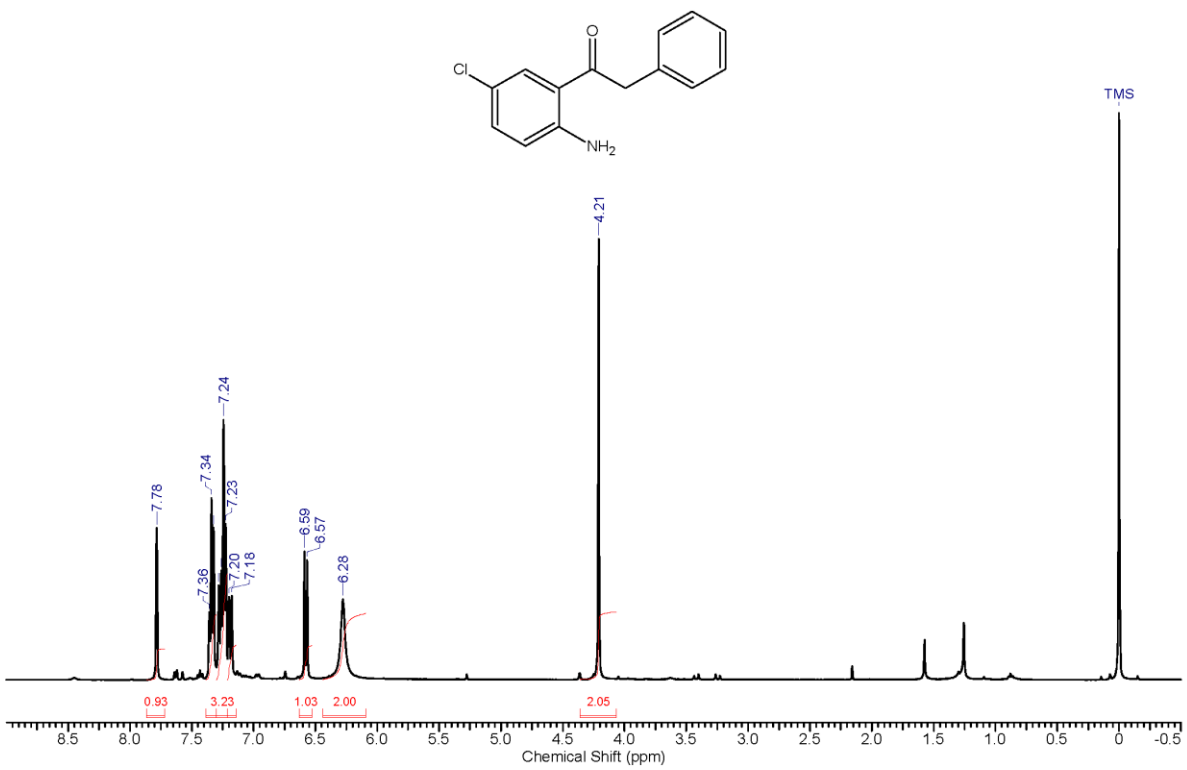

Cl
O
NH2
TMS
4.21
7.78
7.34
7.24
7.23
7.36
7.20
7.18
6.59
6.57
6.28
0.93
3.23
1.03
2.00
2.05
Chemical Shift (ppm)

6d

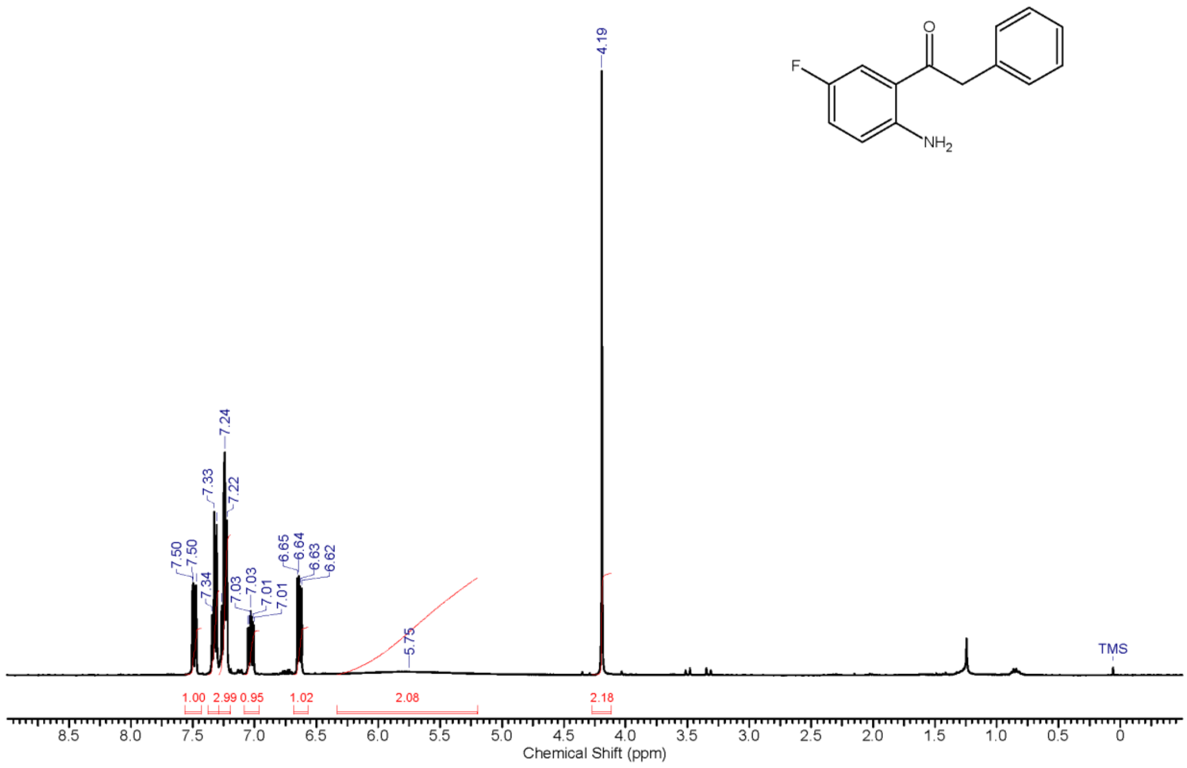

F
O
NH2
4.19
7.24
7.33
7.22
7.50
7.50
7.34
7.03
7.03
7.01
7.01
6.65
6.64
6.63
6.62
5.75
TMS
1.00
2.99
0.95
1.02
2.08
2.18
Chemical Shift (ppm)

6d - $^{19}F\{^{1}H\}$

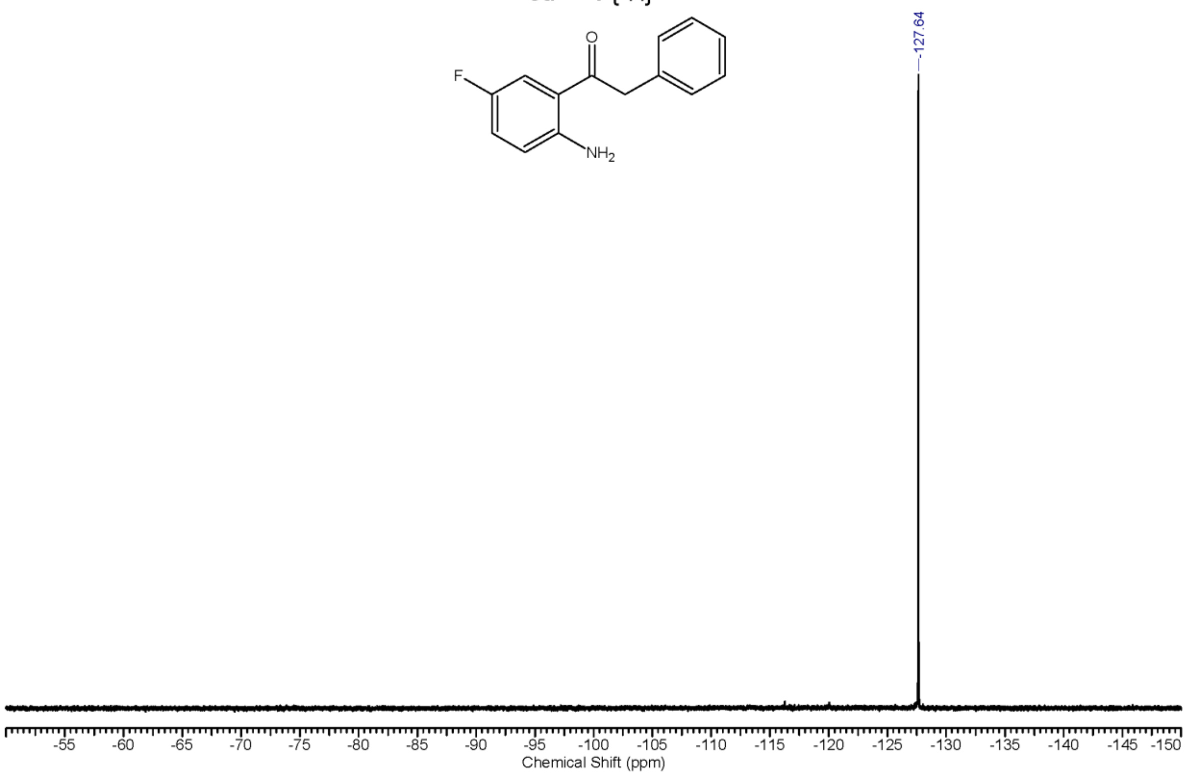

O
F
NH2
-127.64
-55 -60 -65 -70 -75 -80 -85 -90 -95 -100 -105 -110 -115 -120 -125 -130 -135 -140 -145 -150
Chemical Shift (ppm)

6d

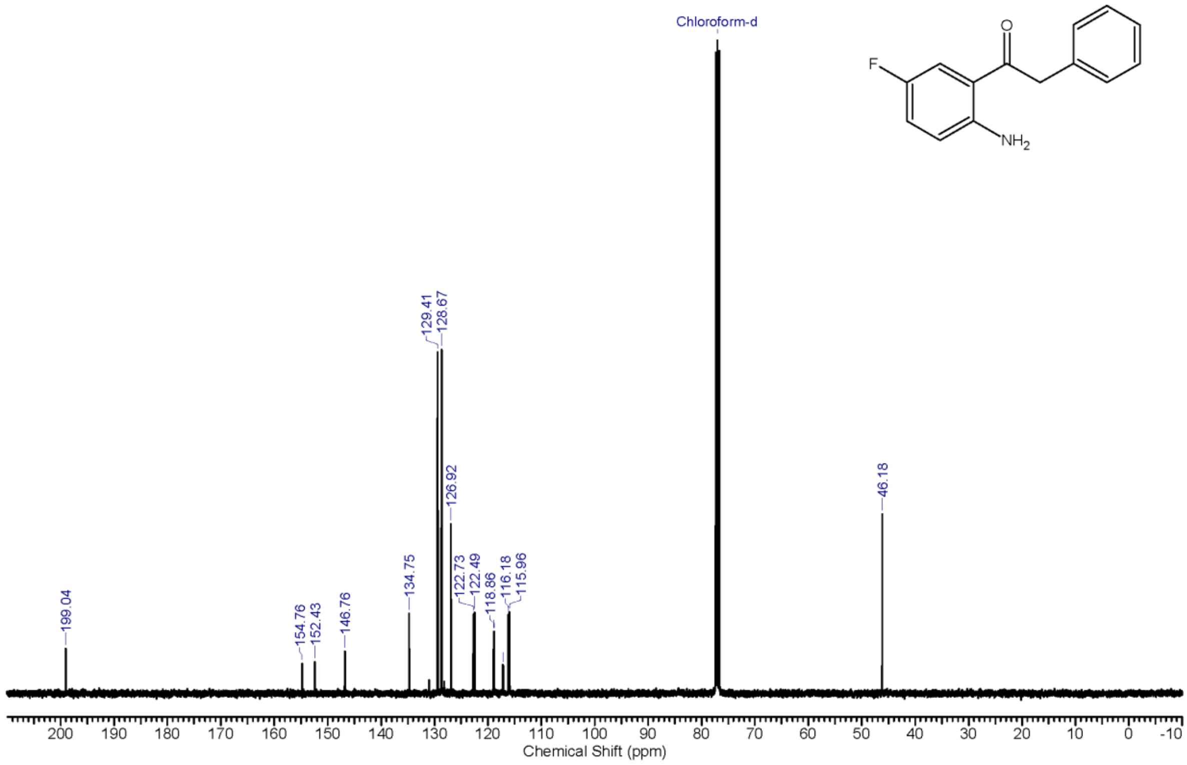

Chloroform-d
O
F
NH2
199.04
154.76
152.43
146.76
134.75
129.41
128.67
126.92
122.73
122.49
118.86
116.18
115.96
46.18
200 190 180 170 160 150 140 130 120 110 100 90 80 70 60 50 40 30 20 10 0 -10
Chemical Shift (ppm)

6e

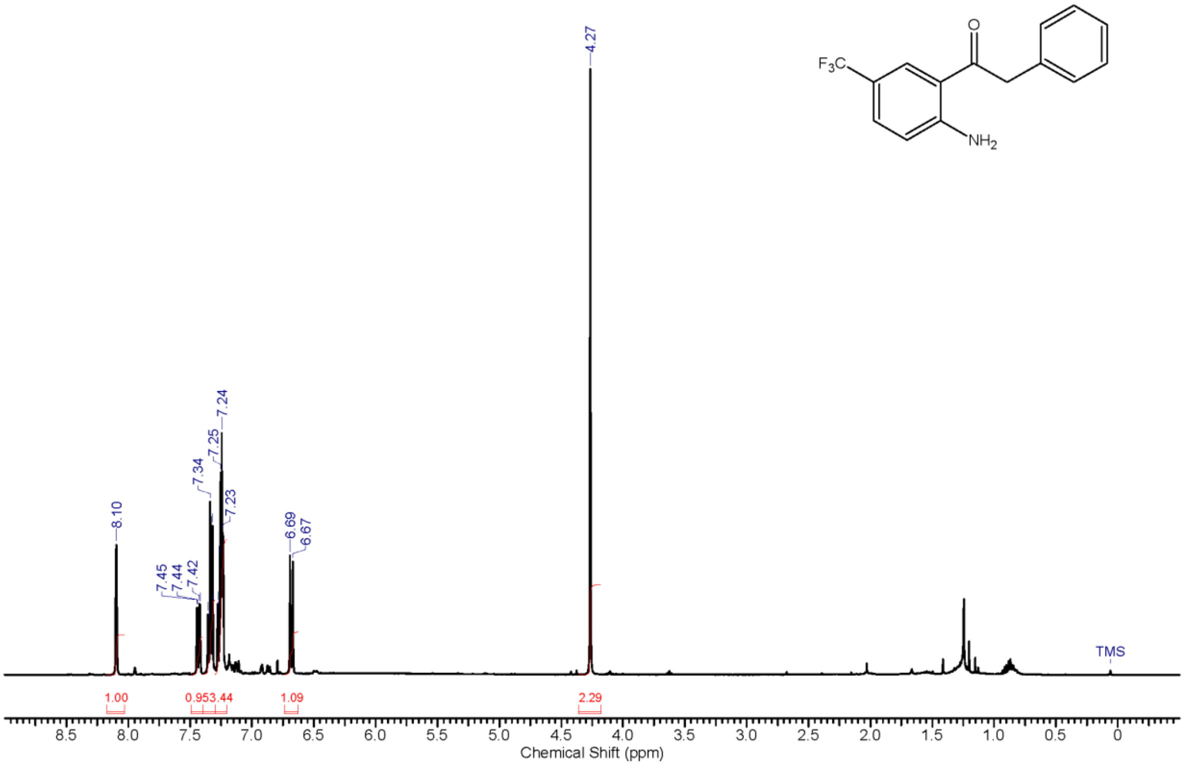
8.10
7.45
7.44
7.42
7.34
7.25
7.24
7.23
6.69
6.67
4.27
TMS
1.00
0.95
3.44
1.09
2.29
Chemical Shift (ppm)
F3C
O
NH2

6e - $^{19}F\{^{1}H\}$

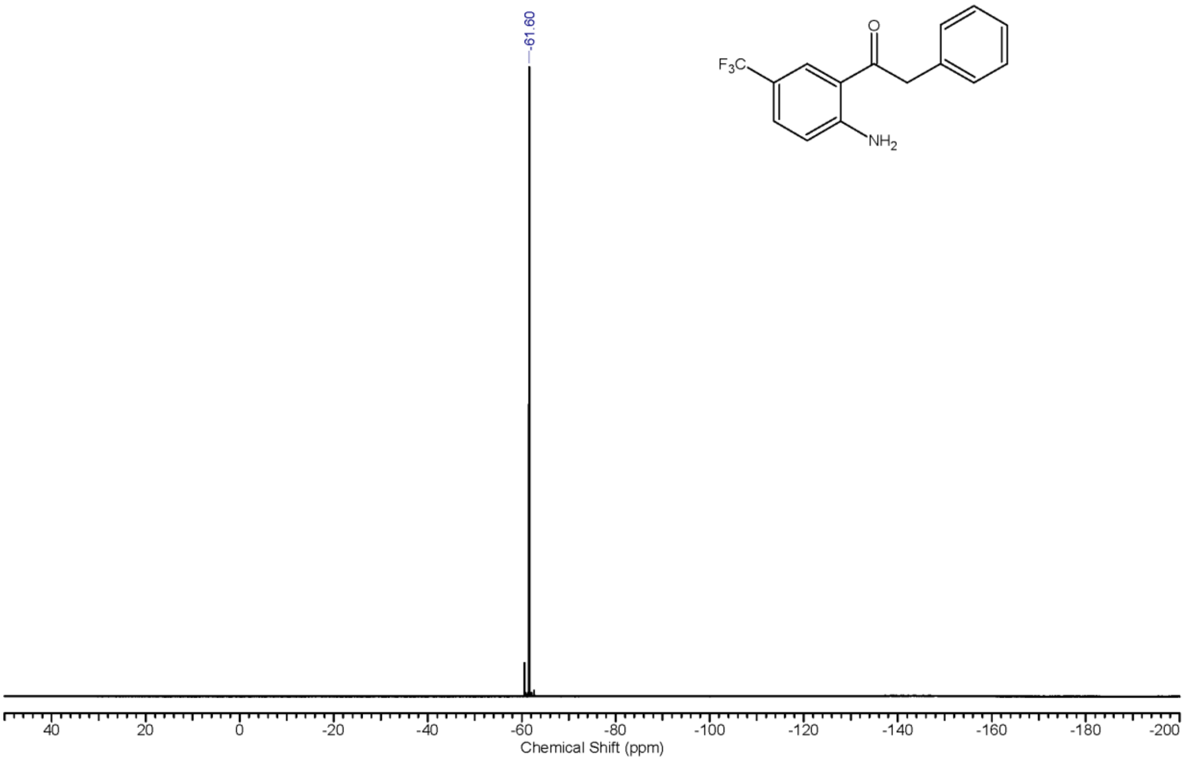
-61.60
Chemical Shift (ppm)
F3C
O
NH2

6e

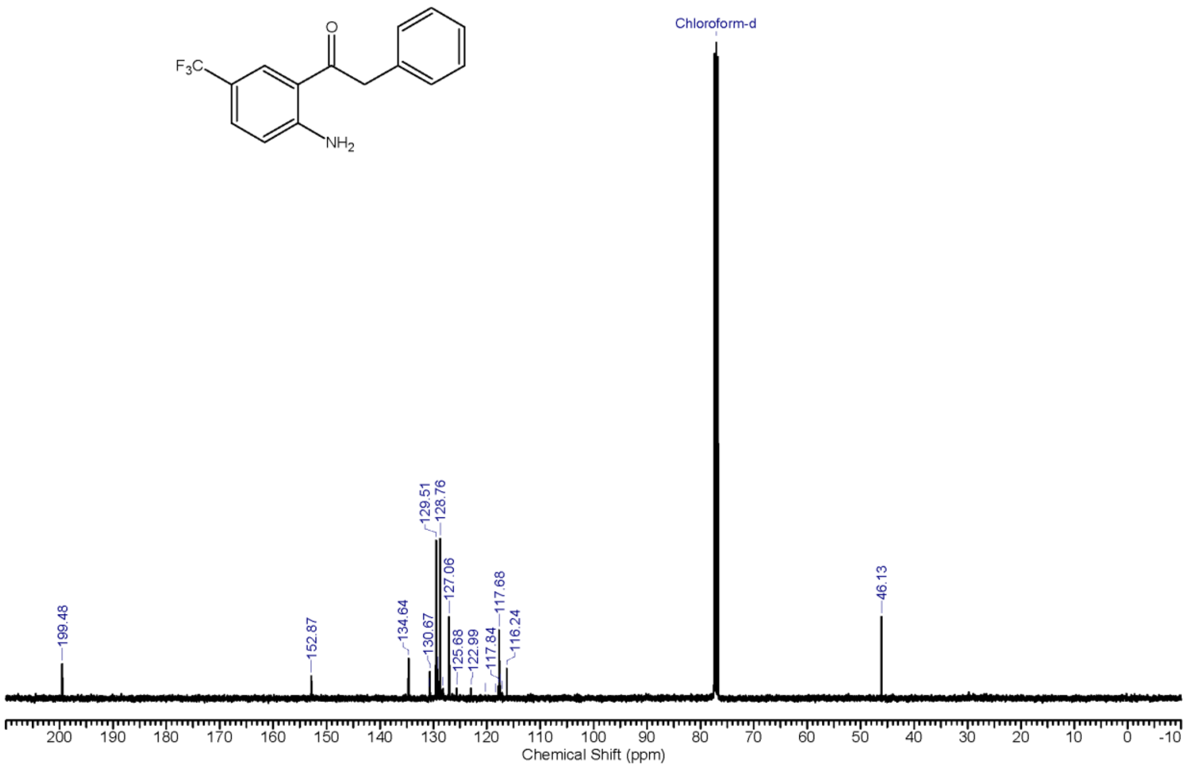
O
F3C
NH2
Chloroform-d
199.48
152.87
134.64
130.67
129.51
128.76
127.06
125.68
122.99
117.84
117.68
116.24
46.13
Chemical Shift (ppm)

6f

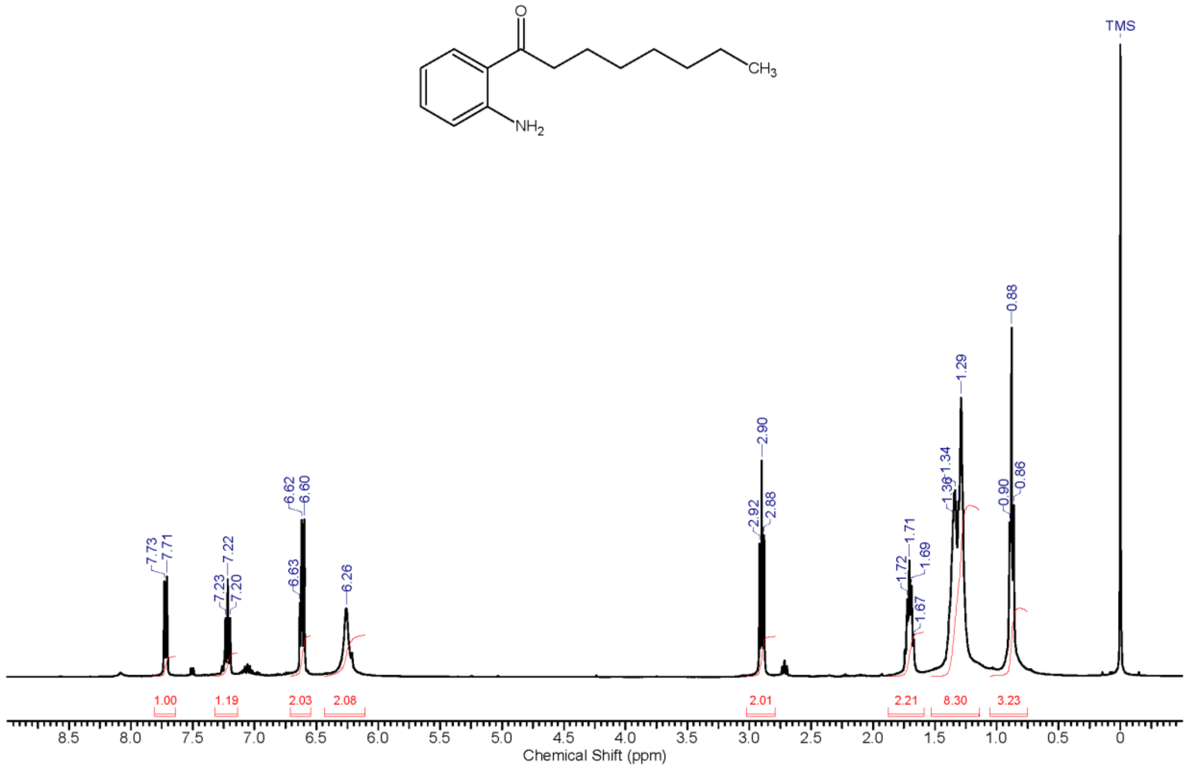
O
CH3
NH2
TMS
7.73
7.71
7.23
7.22
7.20
6.63
6.62
6.60
6.26
2.92
2.90
2.88
1.72
1.71
1.69
1.67
1.36
1.34
1.29
0.90
0.88
0.86
1.00
1.19
2.03
2.08
2.01
2.21
8.30
3.23
Chemical Shift (ppm)

6g

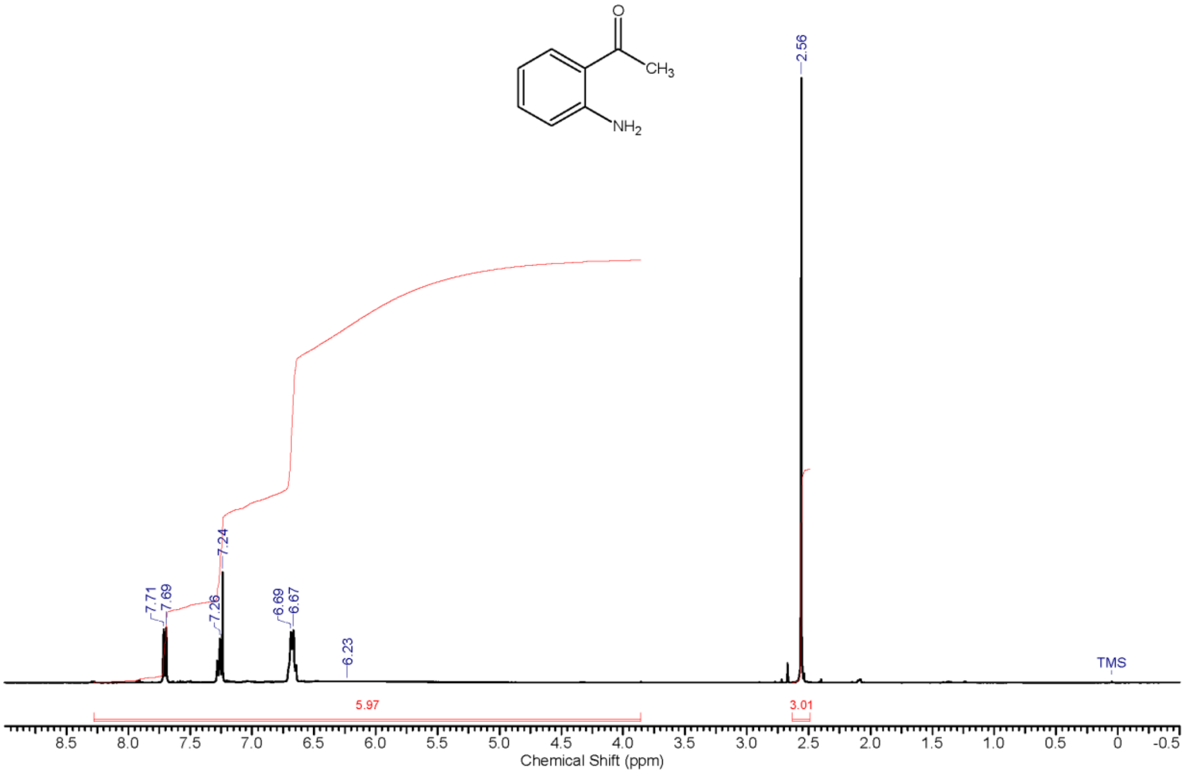
O
CH3
NH2
2.56
7.71
7.69
7.26
7.24
6.69
6.67
6.23
TMS
5.97
3.01
Chemical Shift (ppm)

6h

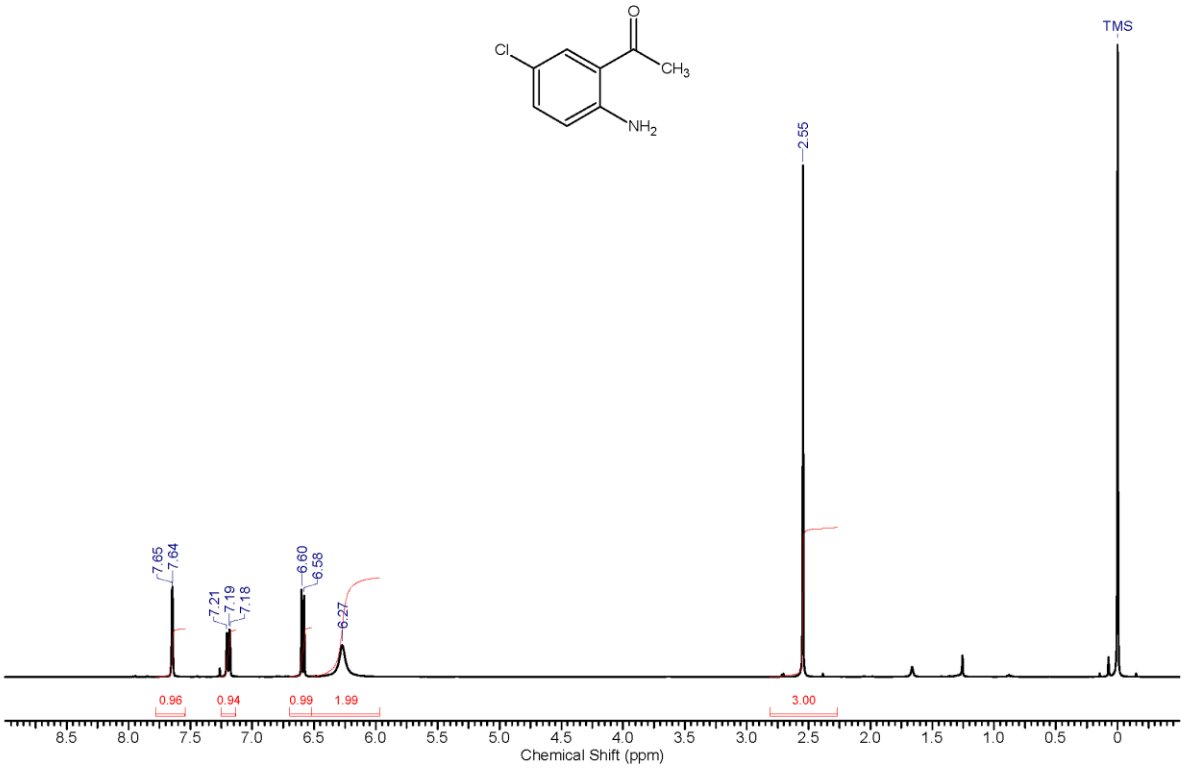
O
Cl
CH3
NH2
TMS
2.55
7.65
7.64
7.21
7.19
7.18
6.60
6.58
6.27
0.96
0.94
0.99
1.99
3.00
Chemical Shift (ppm)

7a+8a

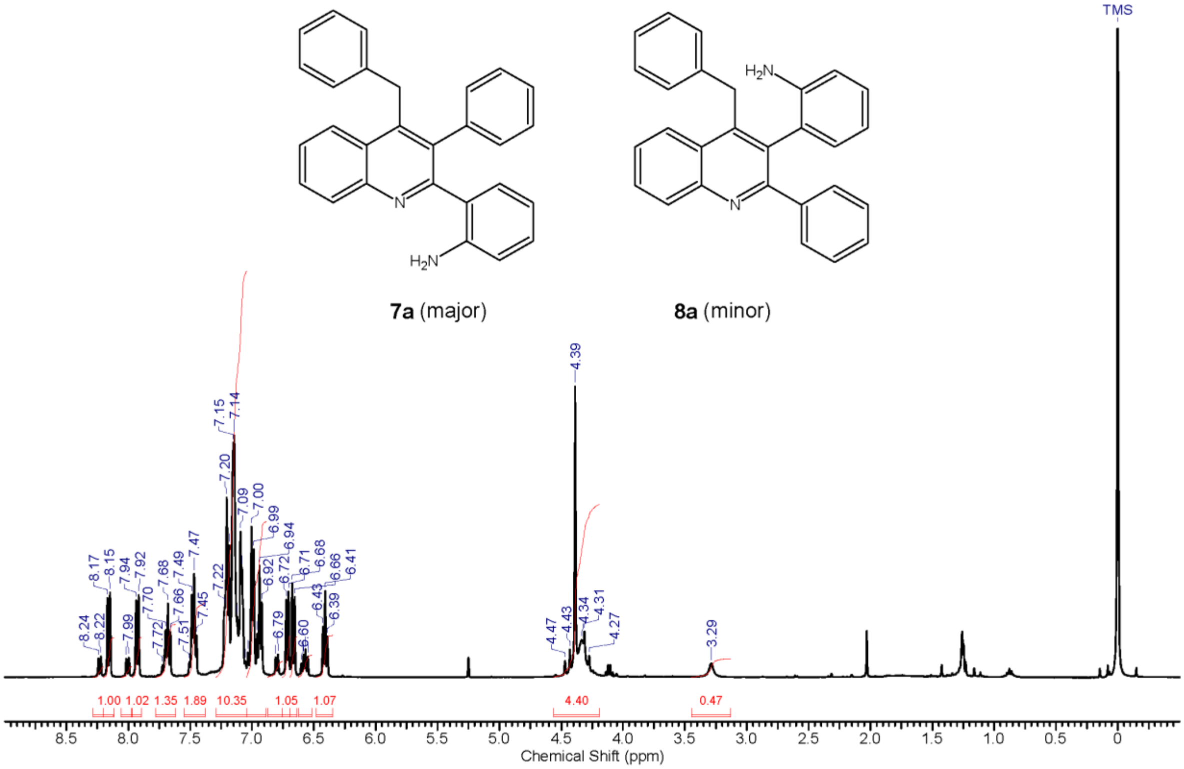
7a (major)
8a (minor)
TMS
Chemical Shift (ppm)

7b+8b

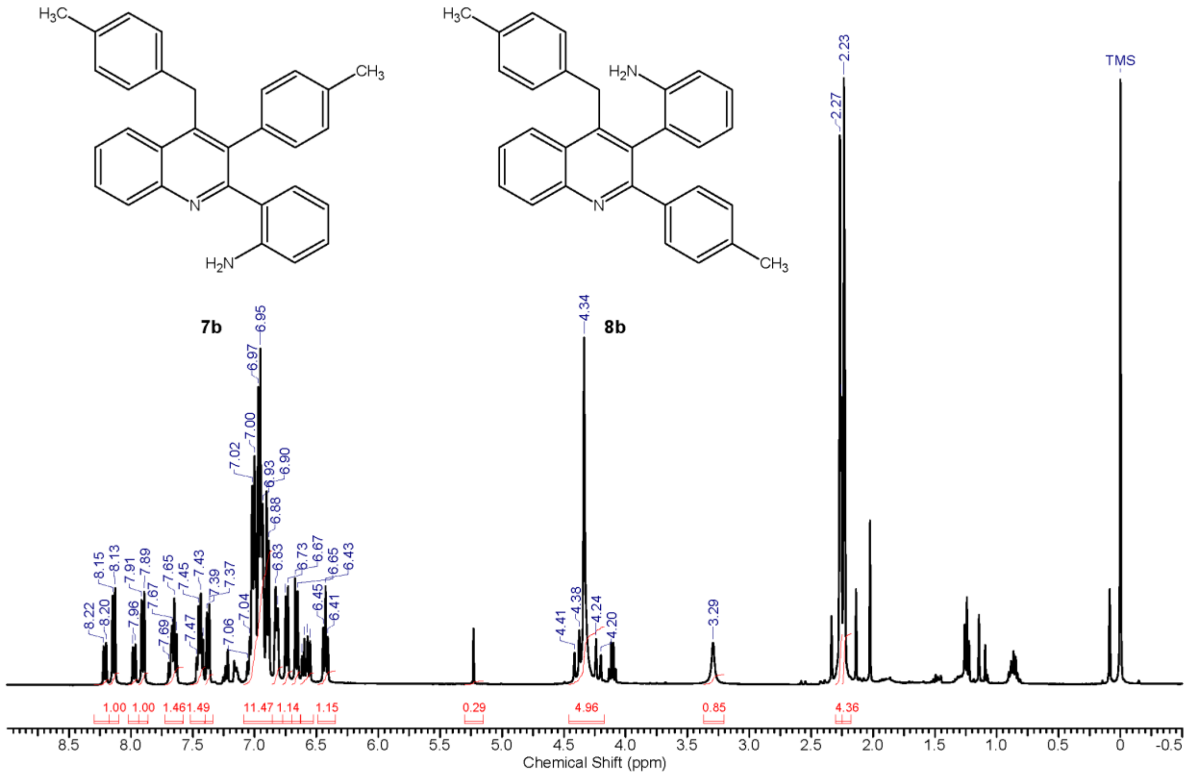
7b
8b
TMS
Chemical Shift (ppm)

7b+8b

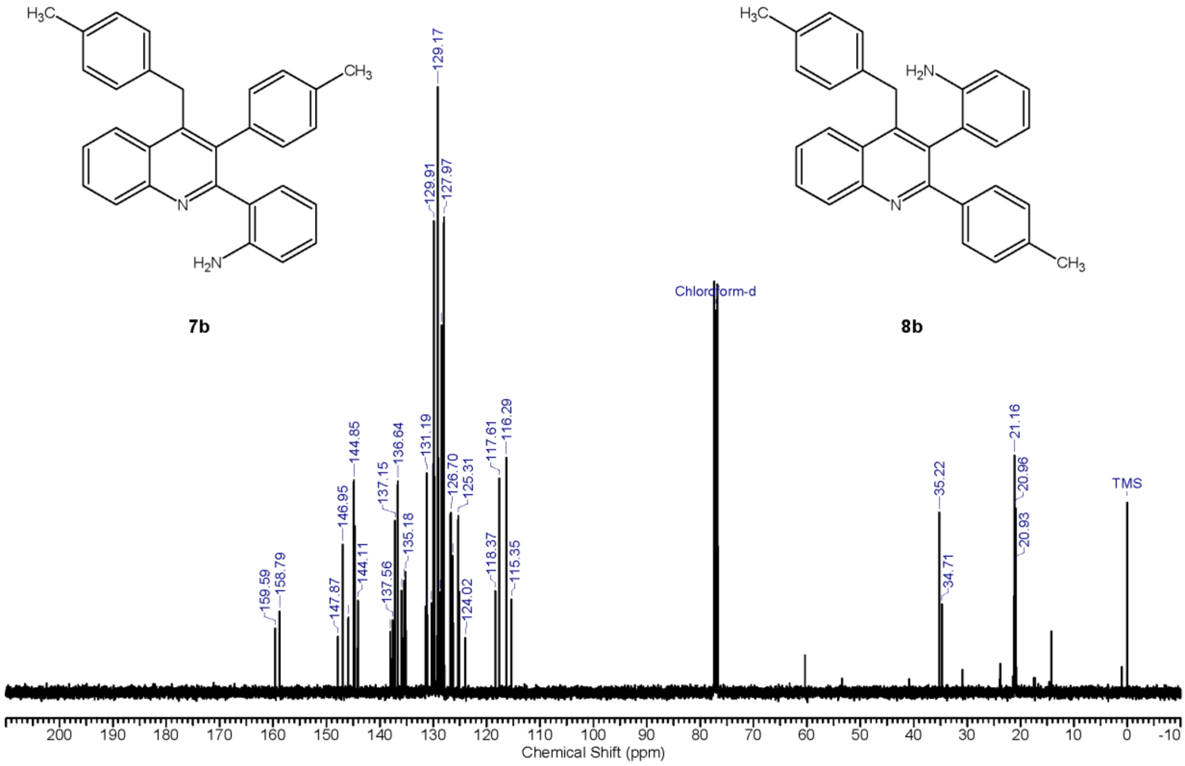
7b
8b
Chloroform-d
TMS
159.59
158.79
147.87
146.95
144.85
144.11
137.56
137.15
136.64
135.18
131.19
129.91
129.17
127.97
126.70
125.31
124.02
118.37
117.61
116.29
115.35
35.22
34.71
21.16
20.96
20.93
Chemical Shift (ppm)

7c+8c

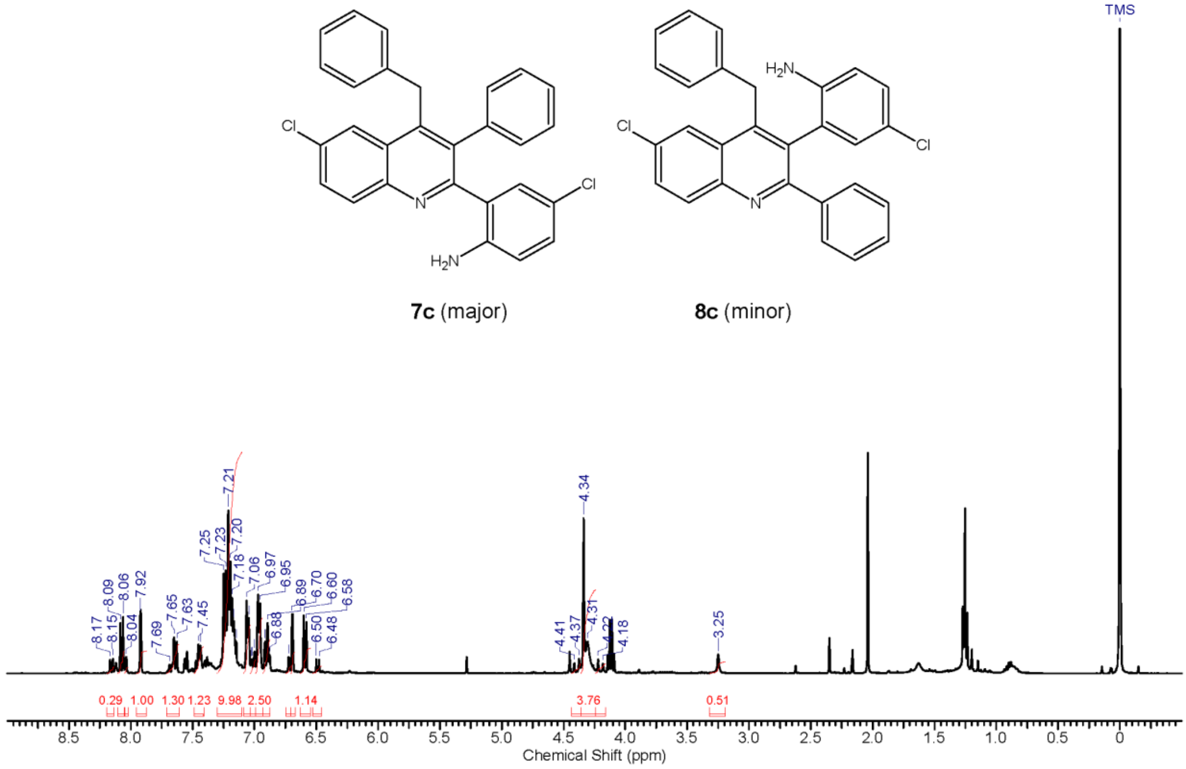
7c (major)
8c (minor)
TMS
8.17
8.15
8.09
8.06
8.04
7.92
7.69
7.65
7.63
7.45
7.25
7.23
7.21
7.20
7.18
7.06
6.97
6.95
6.89
6.88
6.70
6.60
6.58
6.50
6.48
4.41
4.37
4.34
4.31
4.22
4.18
3.25
0.29
1.00
1.30
1.23
9.98
2.50
1.14
3.76
0.51
Chemical Shift (ppm)

7d+8d

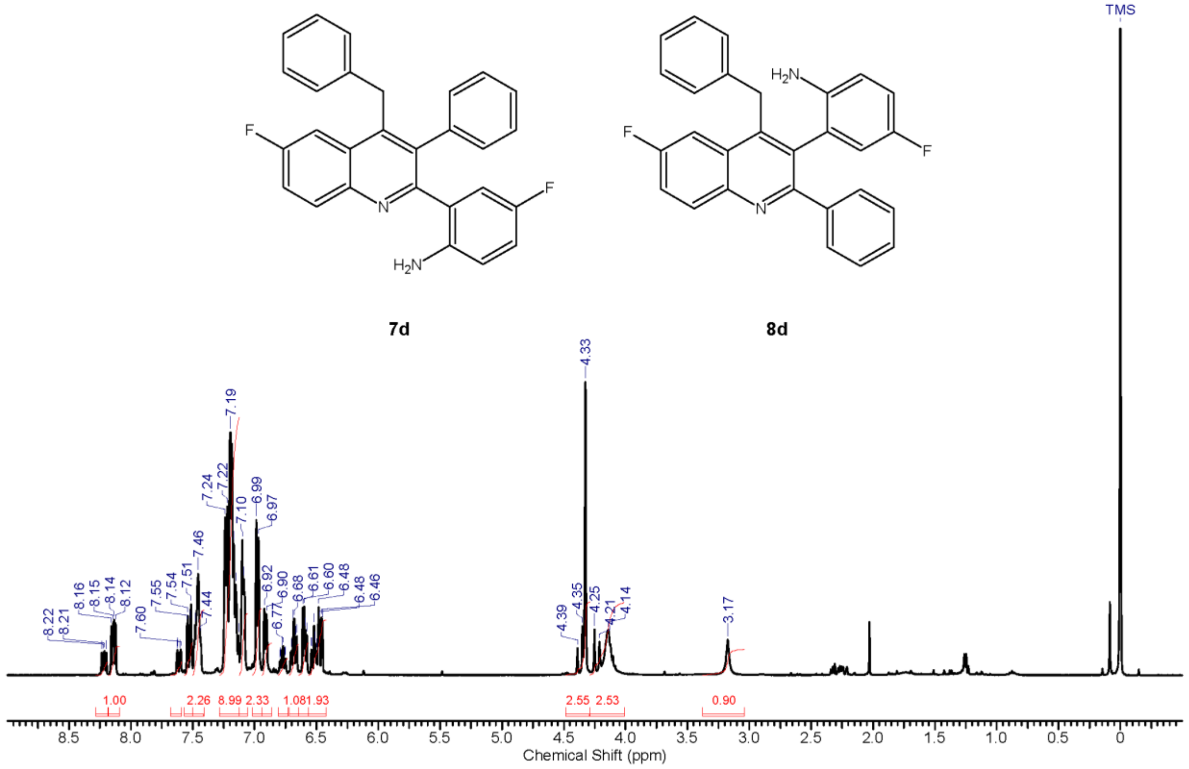

7d+8d - $^{19}F\{^{1}H\}$

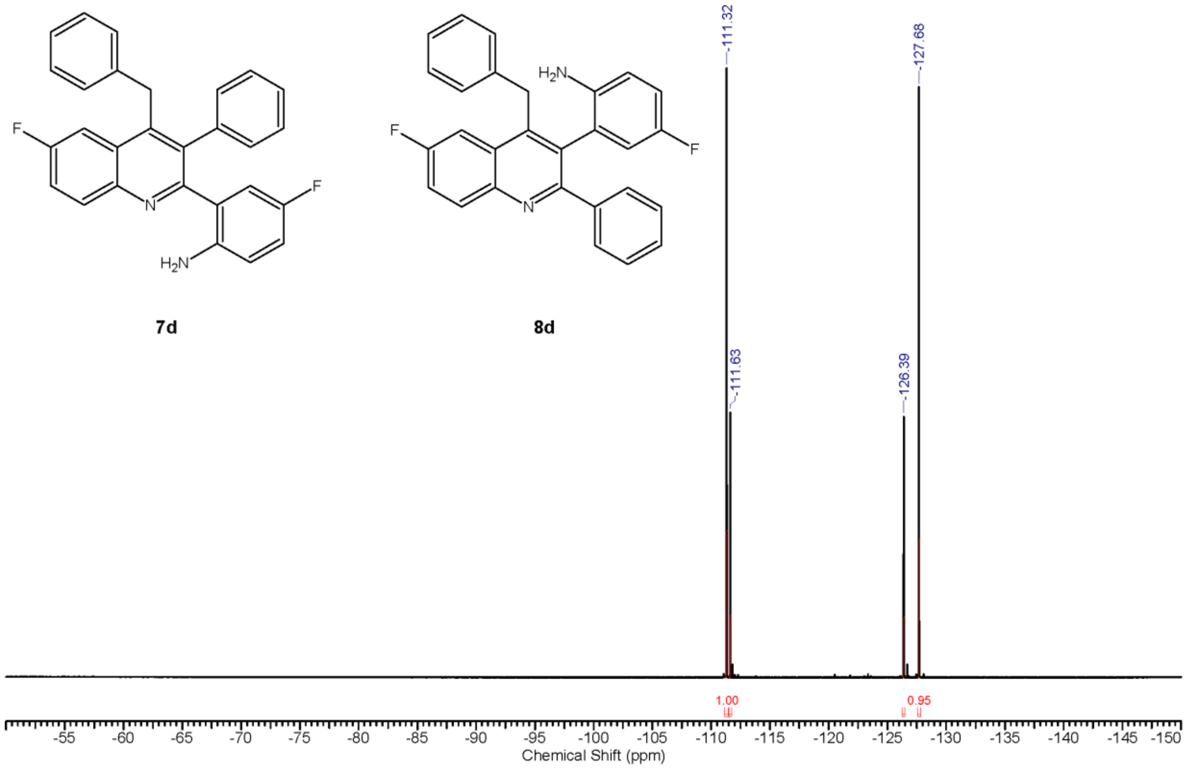

7d+8d

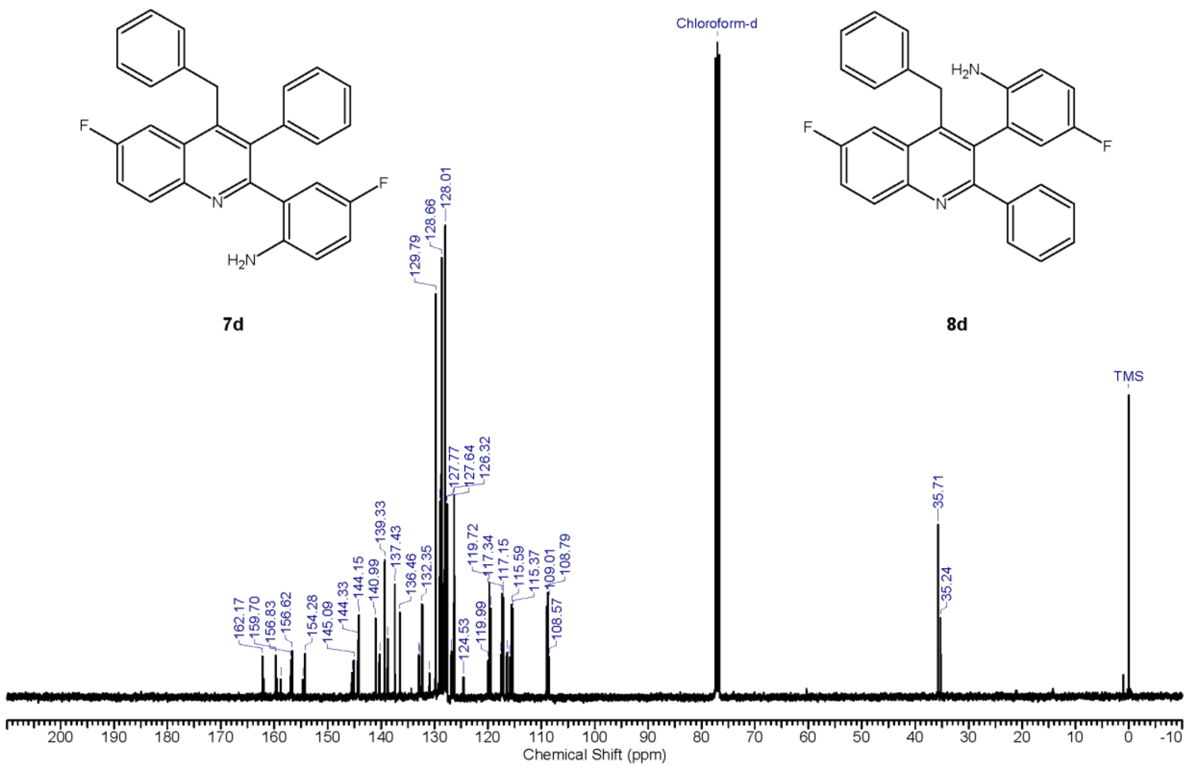

7d
8d
Chloroform-d
TMS
Chemical Shift (ppm)

7e

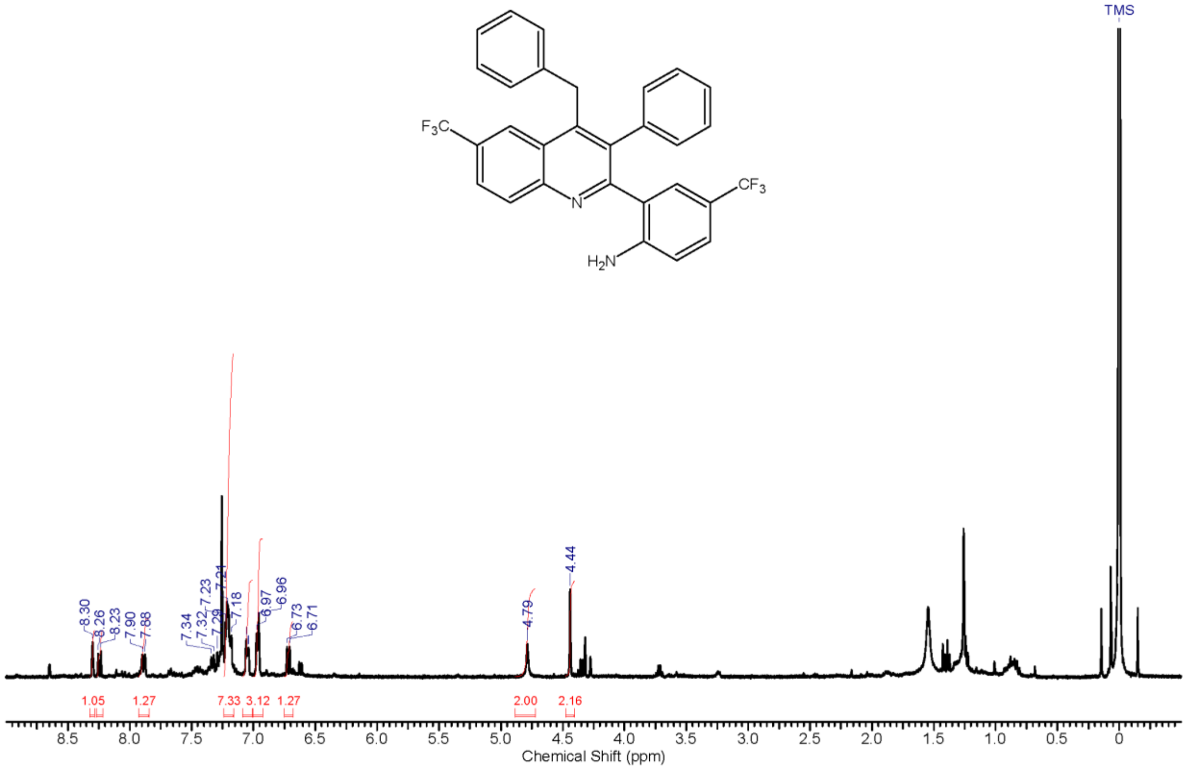

TMS
Chemical Shift (ppm)

7e - ^{19}F{^{1}H}

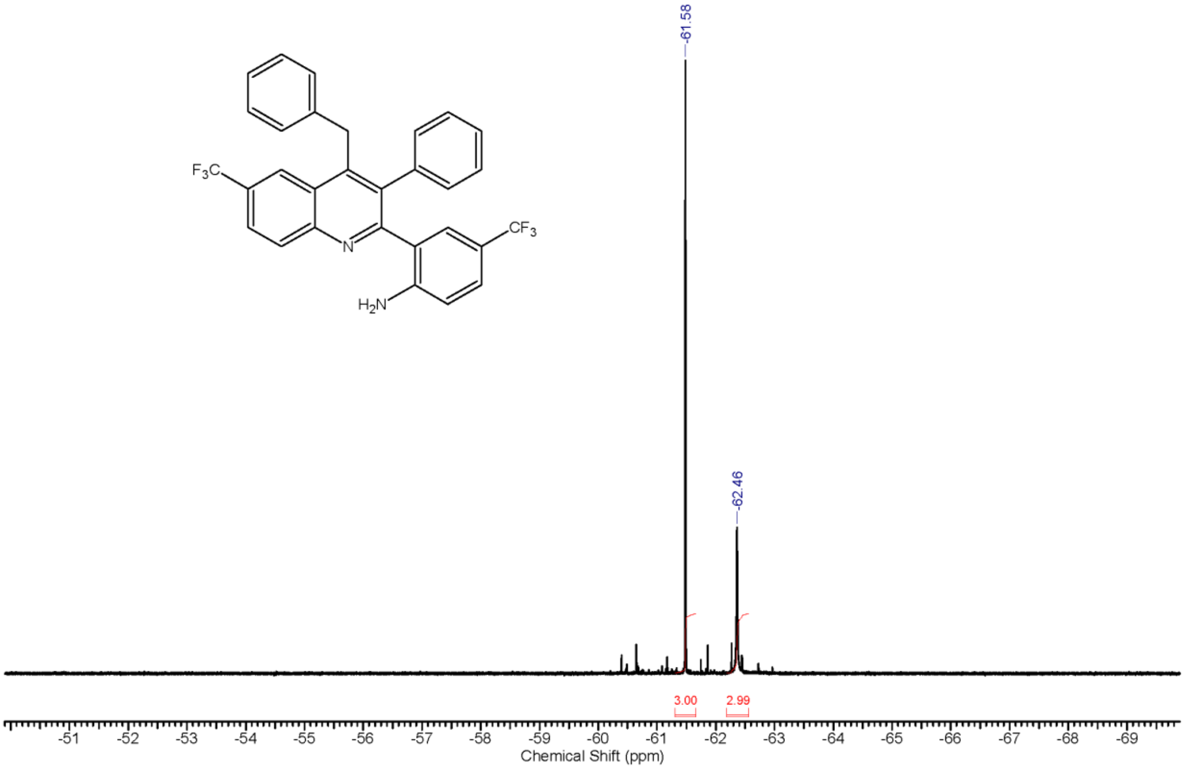

F3C
CF3
N
H2N
-61.58
-62.46
3.00
2.99
-51 -52 -53 -54 -55 -56 -57 -58 -59 -60 -61 -62 -63 -64 -65 -66 -67 -68 -69
Chemical Shift (ppm)

7f

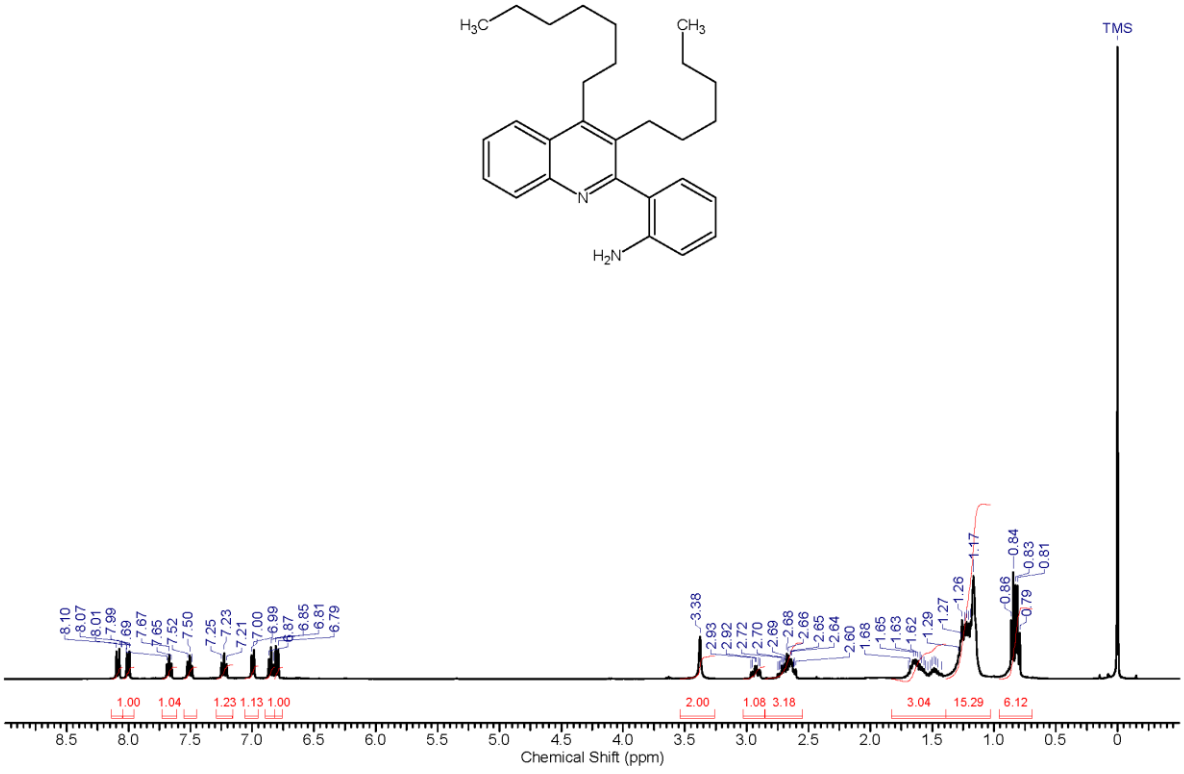

H3C
CH3
N
H2N
TMS
8.10 8.07 8.01 7.99 7.69 7.67 7.65 7.52 7.50 7.25 7.23 7.21 7.00 6.99 6.87 6.85 6.81 6.79
3.38 2.93 2.92 2.72 2.70 2.69 2.68 2.66 2.65 2.64 2.60 1.68 1.65 1.63 1.62 1.29 1.27 1.26 1.17 0.86 0.84 0.83 0.81 0.79
1.00 1.04 1.23 1.13 1.00
2.00 1.08 3.18 3.04 15.29 6.12
8.5 8.0 7.5 7.0 6.5 6.0 5.5 5.0 4.5 4.0 3.5 3.0 2.5 2.0 1.5 1.0 0.5 0
Chemical Shift (ppm)

7f

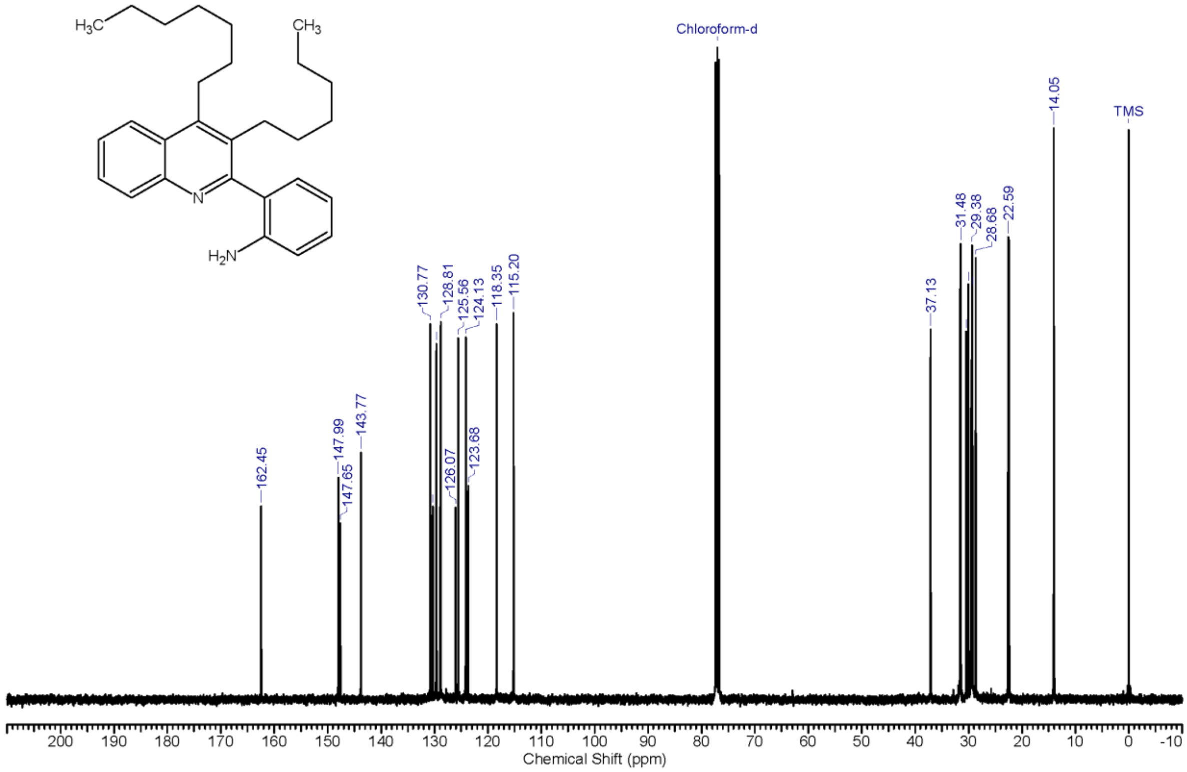
Chloroform-d
TMS
162.45
147.99
147.65
143.77
130.77
126.07
128.81
125.56
124.13
123.68
118.35
115.20
37.13
31.48
29.38
28.68
22.59
14.05
Chemical Shift (ppm)

7g

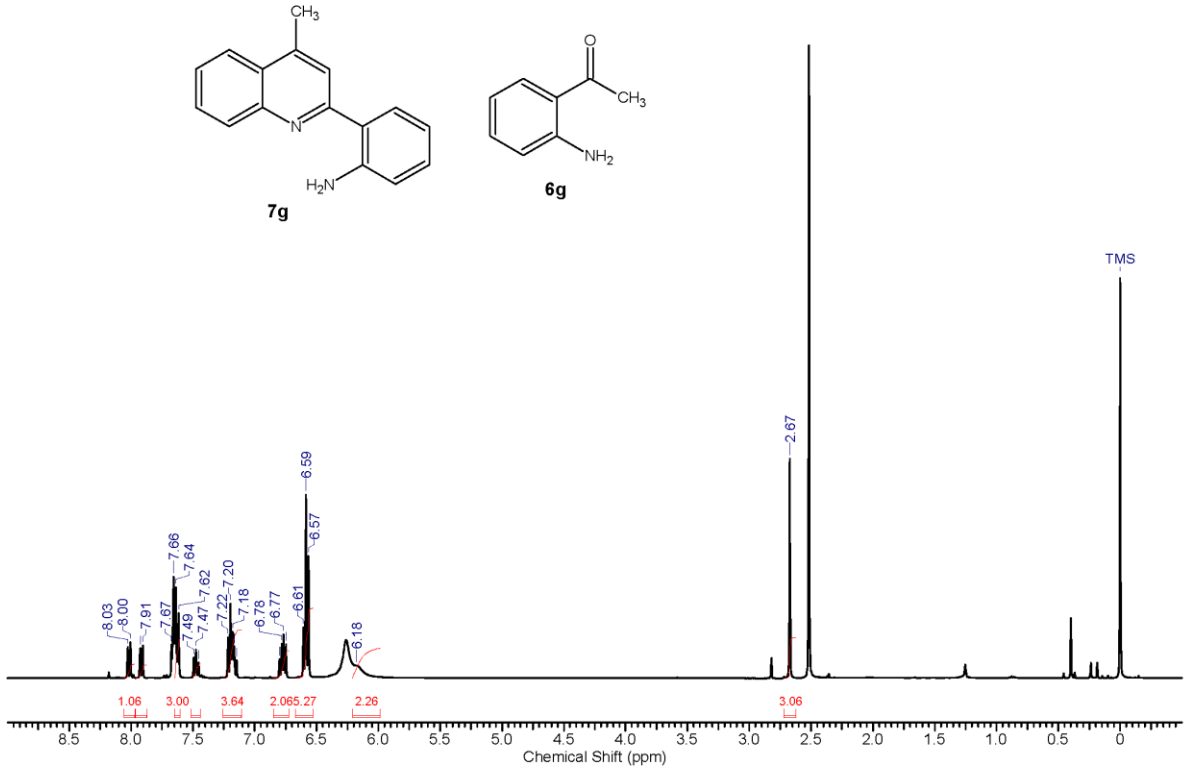
7g
6g
TMS
2.67
6.59
6.57
6.61
6.18
7.66
7.64
7.62
7.67
7.49
7.47
7.22
7.20
7.18
6.78
6.77
8.03
8.00
7.91
1.06
3.00
3.64
2.06
5.27
2.26
3.06
Chemical Shift (ppm)

7h

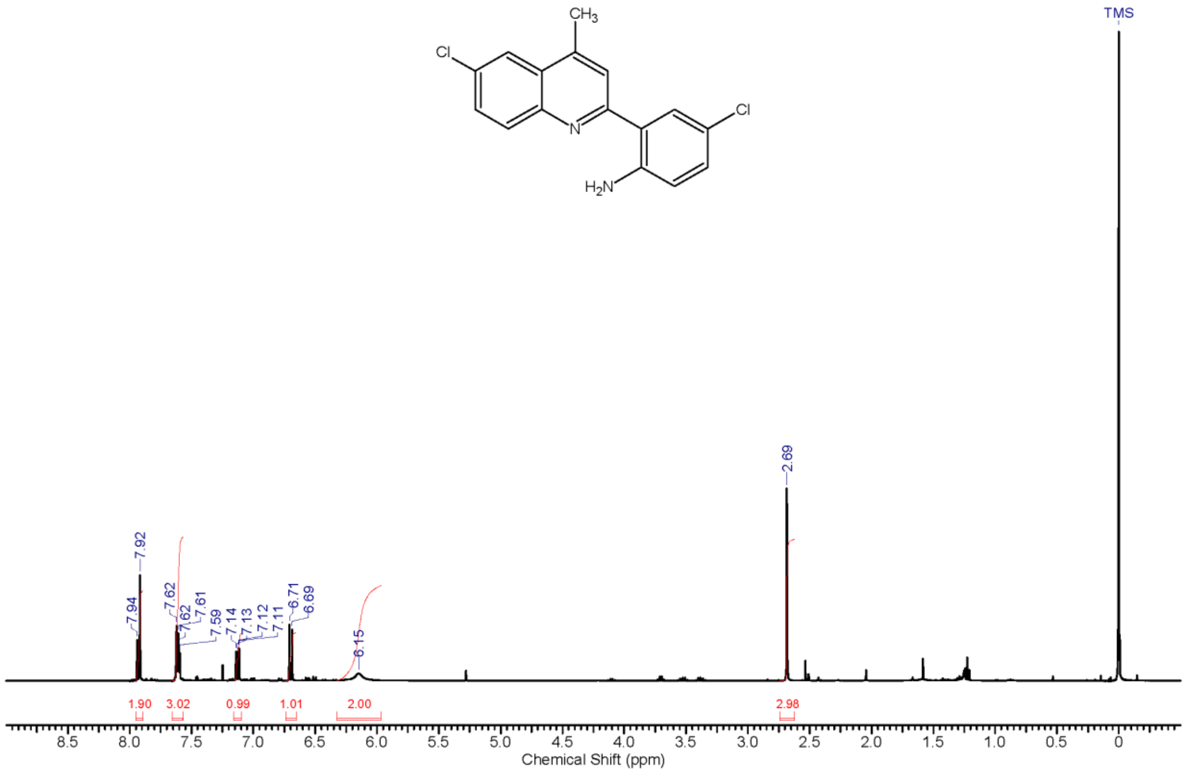
CH3
Cl
N
Cl
H2N
TMS
2.69
7.94
7.92
7.62
7.62
7.61
7.59
7.14
7.13
7.12
7.11
6.71
6.69
6.15
1.90
3.02
0.99
1.01
2.00
2.98
Chemical Shift (ppm)

8d

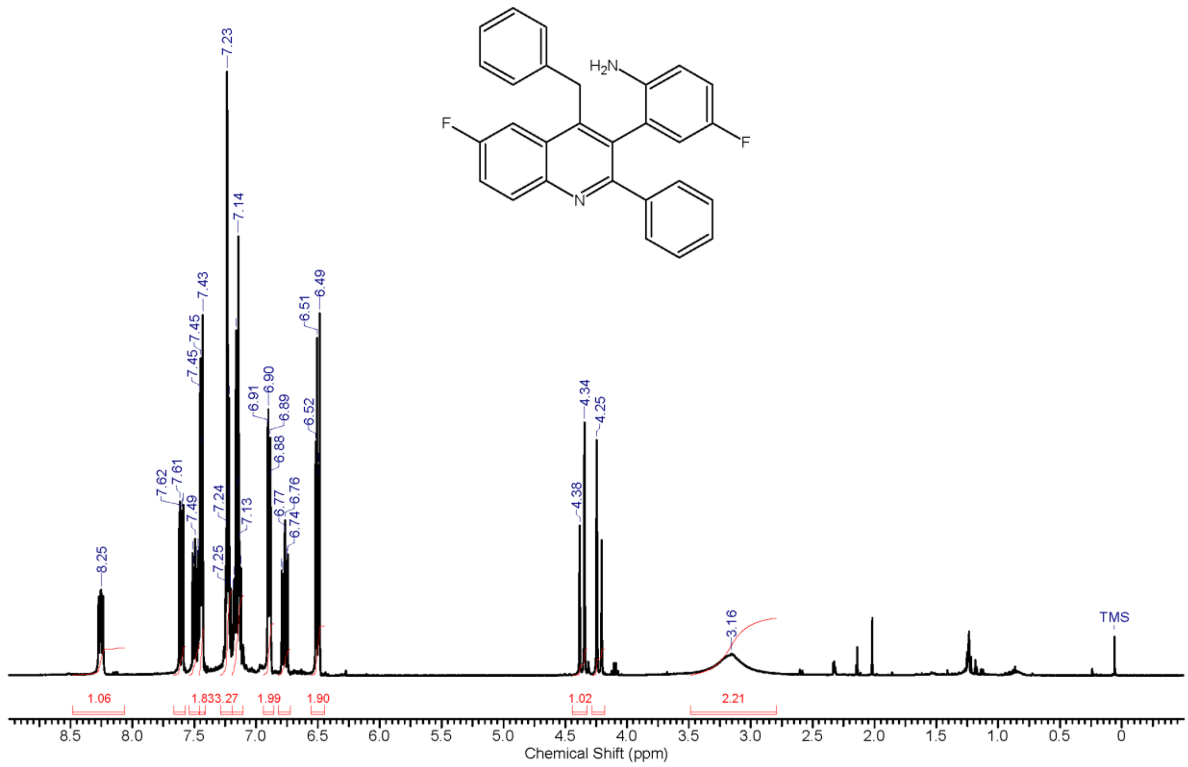
H2N
F
F
N
7.23
7.14
7.43
7.45
7.45
6.51
6.49
6.91
6.90
6.89
6.88
6.52
7.62
7.61
7.49
7.24
7.25
7.13
6.77
6.76
6.74
8.25
4.34
4.25
4.38
3.16
TMS
1.06
1.83
3.27
1.99
1.90
1.02
2.21
Chemical Shift (ppm)

8d - $^{19}F\{^{1}H\}$

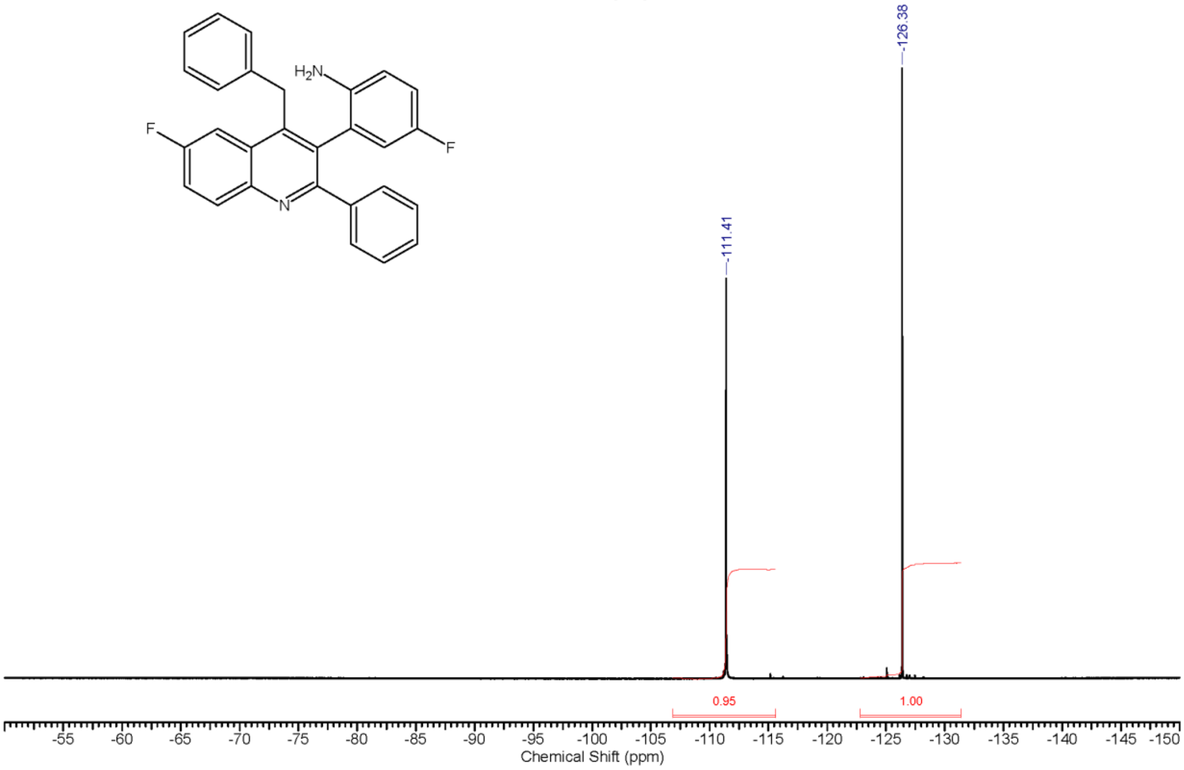

H2N
F
F
N
-111.41
-126.38
0.95
1.00
-55 -60 -65 -70 -75 -80 -85 -90 -95 -100 -105 -110 -115 -120 -125 -130 -135 -140 -145 -150
Chemical Shift (ppm)

8d

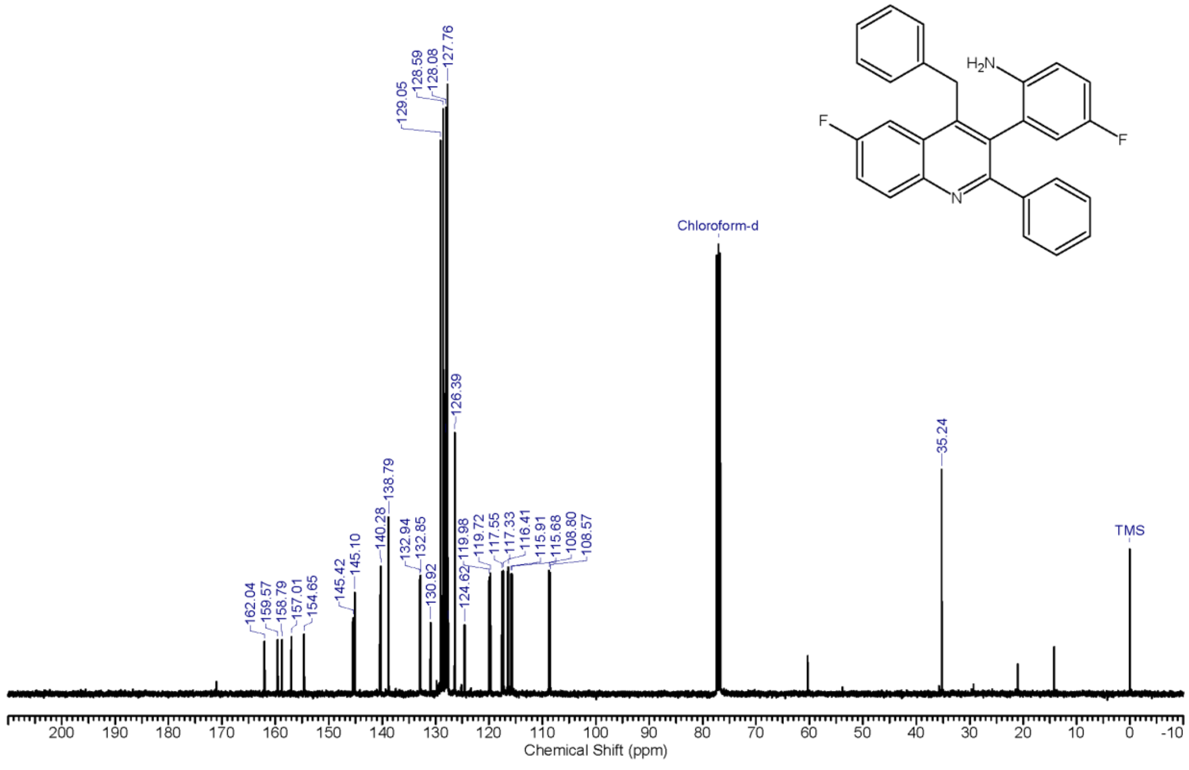

H2N
F
F
N
162.04
159.57
158.79
157.01
154.65
145.42
145.10
140.28
138.79
132.94
132.85
130.92
129.05
128.59
128.08
127.76
126.39
124.62
119.98
119.72
117.55
117.33
116.41
115.91
115.68
108.80
108.57
Chloroform-d
35.24
TMS
200 190 180 170 160 150 140 130 120 110 100 90 80 70 60 50 40 30 20 10 0 -10
Chemical Shift (ppm)

9a

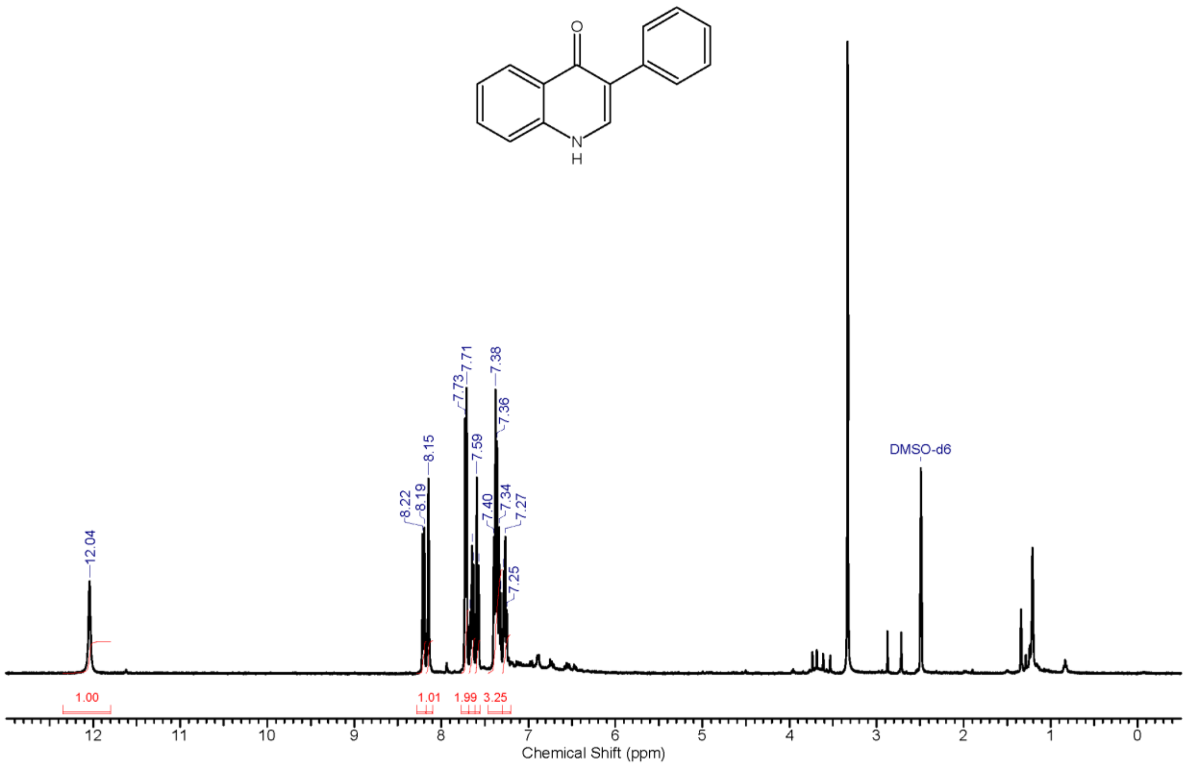
O
N
H
12.04
8.22
8.19
8.15
7.73
7.71
7.59
7.40
7.38
7.36
7.34
7.27
7.25
DMSO-d6
1.00
1.01
1.99
3.25
Chemical Shift (ppm)

10a

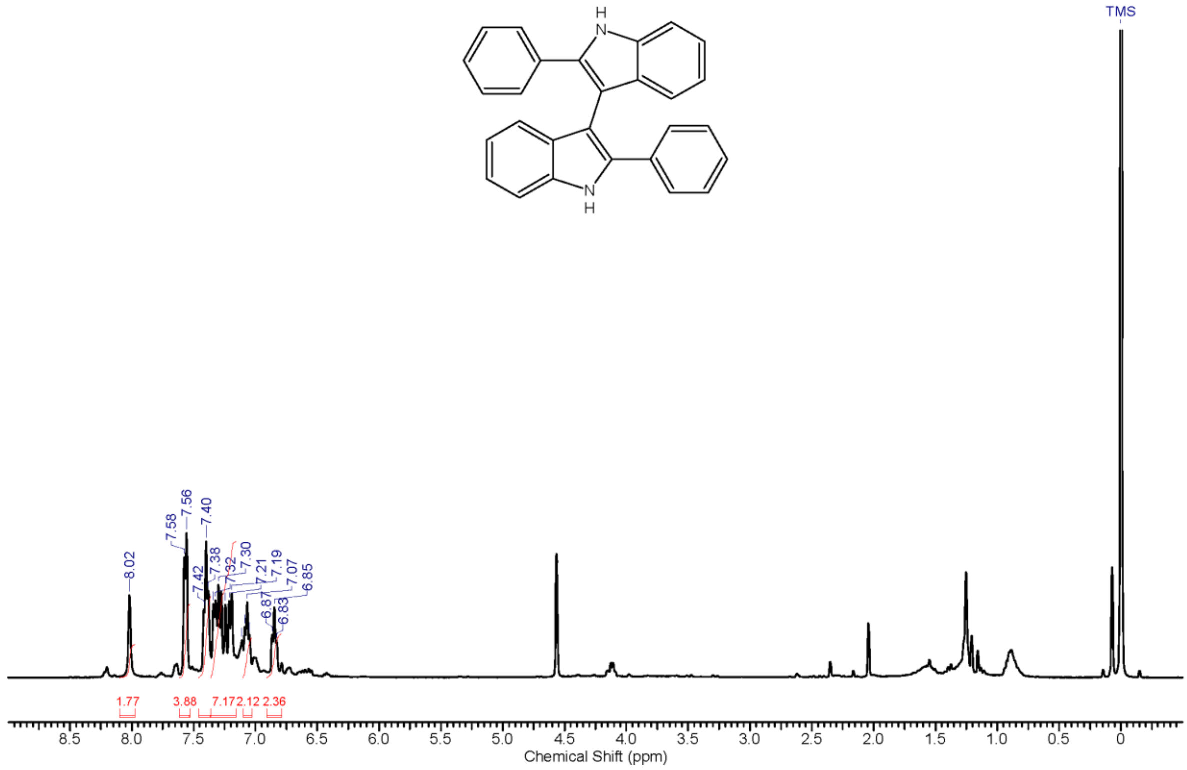
H
N
N
H
TMS
8.02
7.58
7.56
7.42
7.40
7.38
7.32
7.30
7.21
7.19
7.07
6.87
6.85
6.83
1.77
3.88
7.17
2.12
2.36
Chemical Shift (ppm)

11g

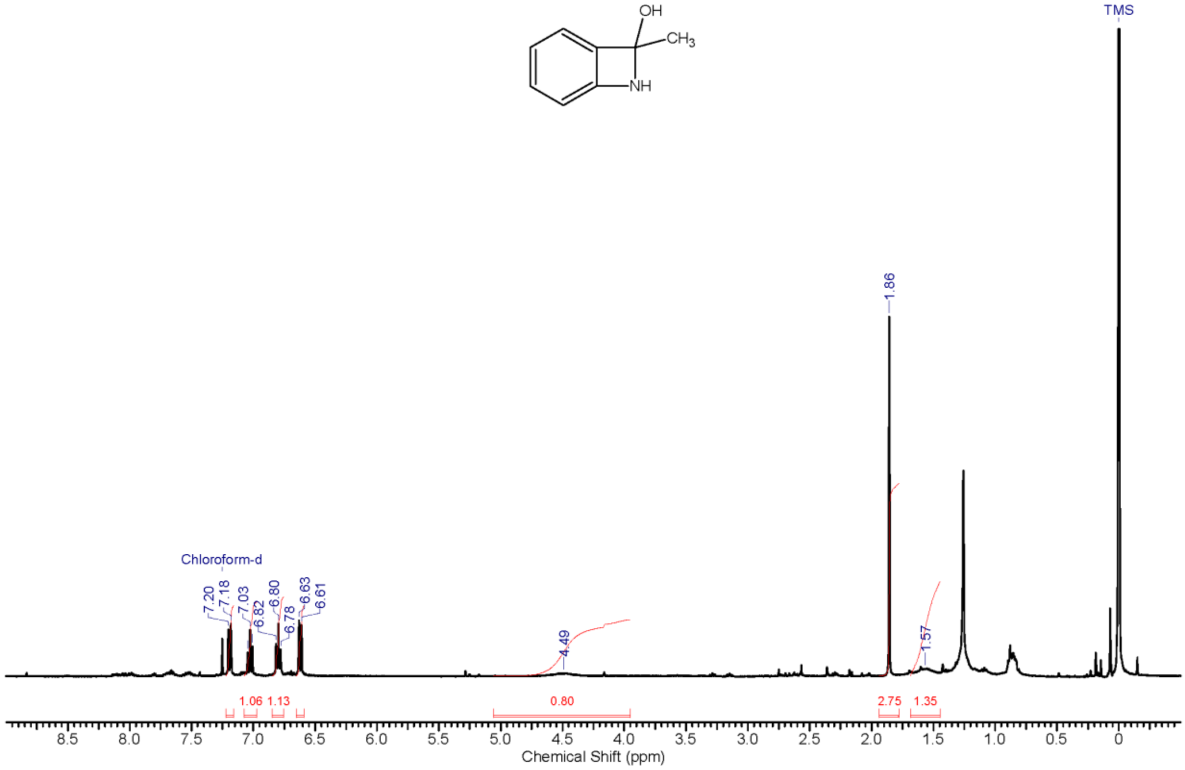

OH
CH3
NH
TMS
1.86
Chloroform-d
7.20
7.18
7.03
6.82
6.80
6.78
6.63
6.61
4.49
1.57
1.06 1.13
0.80
2.75 1.35
8.5 8.0 7.5 7.0 6.5 6.0 5.5 5.0 4.5 4.0 3.5 3.0 2.5 2.0 1.5 1.0 0.5 0
Chemical Shift (ppm)

11h

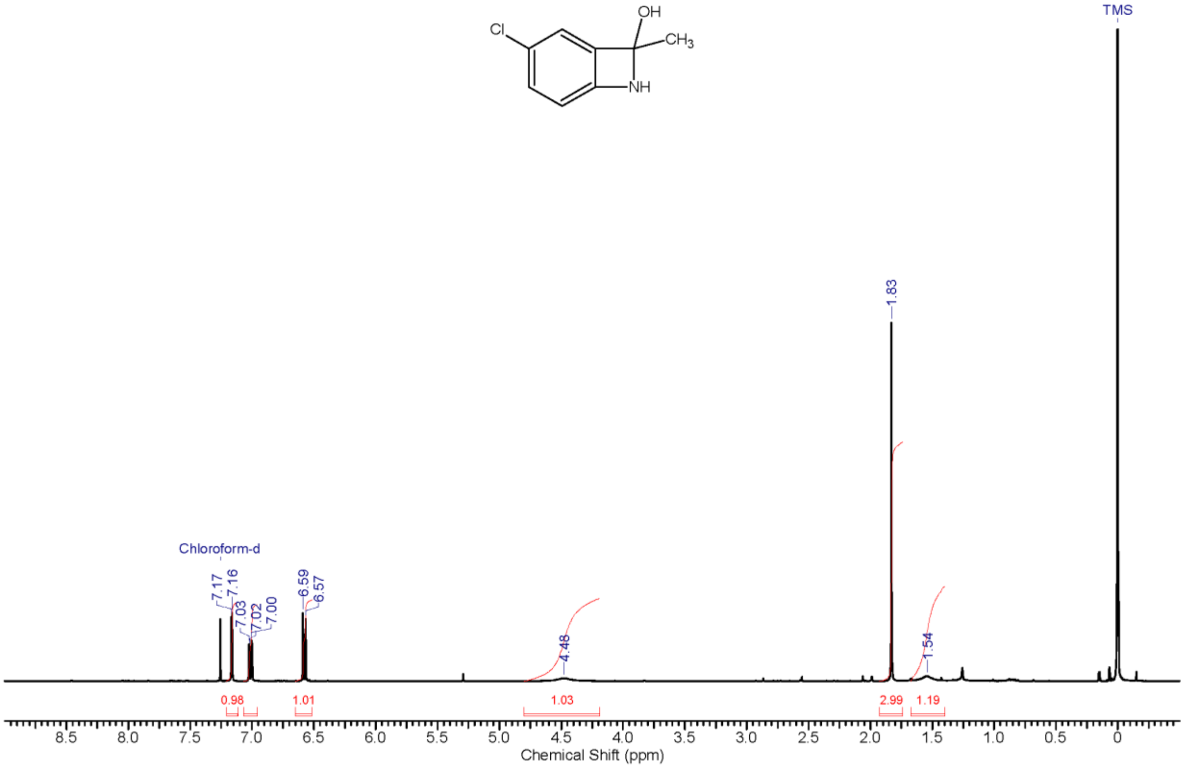

Cl
OH
CH3
NH
TMS
1.83
Chloroform-d
7.17
7.16
7.03
7.02
7.00
6.59
6.57
4.48
1.54
0.98
1.01
1.03
2.99 1.19
8.5 8.0 7.5 7.0 6.5 6.0 5.5 5.0 4.5 4.0 3.5 3.0 2.5 2.0 1.5 1.0 0.5 0
Chemical Shift (ppm)

11h

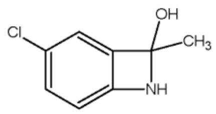

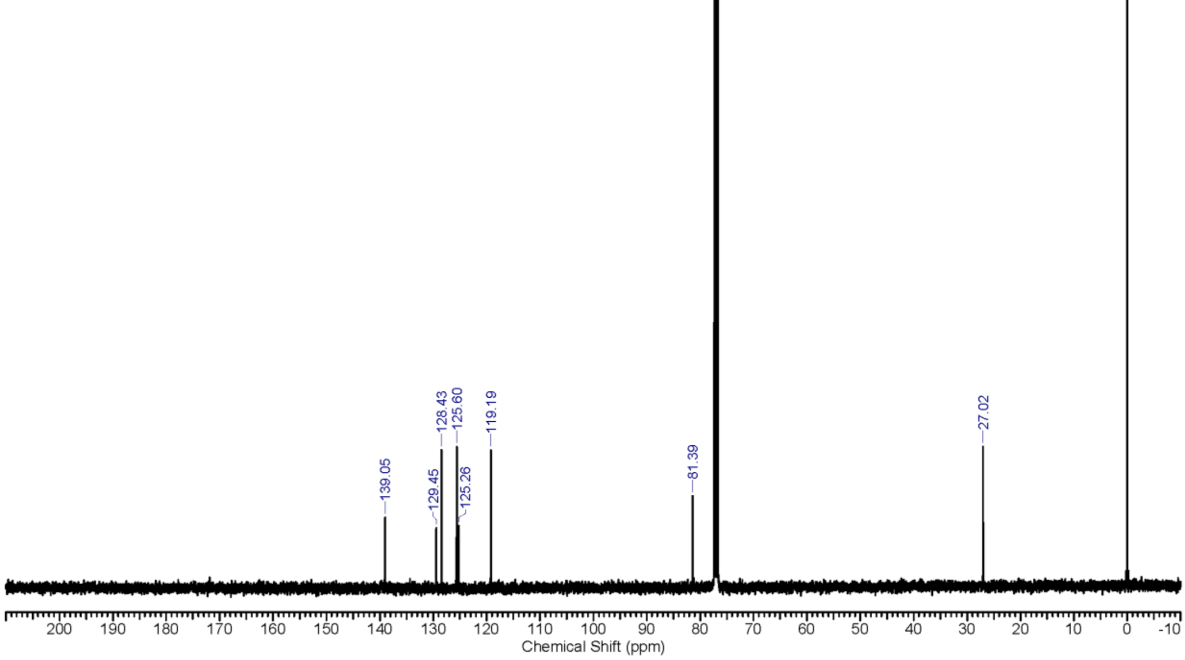

Chloroform-d
TMS
139.05
129.45
128.43
125.60
125.26
119.19
81.39
27.02
200 190 180 170 160 150 140 130 120 110 100 90 80 70 60 50 40 30 20 10 0 -10
Chemical Shift (ppm)

III. Optimized cartesian coordinates of all the investigated species.

1a

6	1.875482	-0.288116	-0.039166
6	2.566862	-1.501520	-0.154433
6	3.952438	-1.539719	-0.146456
6	4.662949	-0.345558	-0.022110
6	3.999096	0.866660	0.091123
6	2.598992	0.921173	0.084080
1	1.990200	-2.416537	-0.247446
1	4.474604	-2.486377	-0.235992
1	5.749035	-0.358071	-0.013465
1	4.563361	1.791555	0.179447
7	1.934168	2.132936	0.150646
6	0.448380	-0.255008	-0.036587
6	-0.761646	-0.180993	-0.023466
6	-2.190715	-0.121055	-0.015709
1	0.961871	2.077376	0.421924
1	2.433599	2.884931	0.601576
6	-2.853314	1.092661	-0.249147
6	-4.241394	1.146581	-0.238526
6	-4.986543	-0.005273	0.002179
6	-4.335670	-1.214976	0.233079
6	-2.947952	-1.276477	0.226250
1	-2.269842	1.987312	-0.443240
1	-4.743721	2.091802	-0.421229
1	-6.071383	0.039498	0.008921
1	-4.912045	-2.115932	0.420706
1	-2.436653	-2.216396	0.407602

Frequencies

12.0228004
41.9788017
51.2673988
131.639893
136.701401
220.025299
248.130707
311.056305
352.641113
396.253113
411.532715
415.859802
485.420685
493.792786
529.835083

544.493225
559.784119
579.025574
584.722290
605.881470
642.820007
711.065002
713.683472
751.076904
773.516907
783.264893
832.650696
870.580505
874.019470
887.530884
945.375610
960.762695
992.004822
993.302002
1018.29968
1027.10059
1068.83826
1074.34497
1100.23987
1122.96484
1173.91846
1192.77173
1203.45459
1205.01685
1223.83264
1307.46216
1337.28979
1355.79248
1366.49524
1371.97925
1372.24609
1505.35046
1521.73816
1554.55005
1571.34631
1659.28845
1664.17065
1686.14001
1698.12500
1710.23865
2361.05151
3202.31104
3211.81177

3218.59375
3220.00122
3226.46924
3229.52515
3237.09155
3242.59448
3243.32129
3610.48096
3721.61011

Ia

6	-1.861254	-0.355846	-0.001266
6	-2.595320	-1.548276	-0.005143
6	-3.984581	-1.515647	-0.004890
6	-4.672231	-0.302912	-0.000812
6	-3.969173	0.897962	0.003090
6	-2.586979	0.840970	0.002750
1	-2.061598	-2.491715	-0.008308
1	-4.539239	-2.447660	-0.007909
1	-5.756285	-0.288242	-0.000654
1	-4.496378	1.847849	0.006279
7	-1.792758	2.089341	0.006770
6	-0.432285	-0.312467	-0.001159
6	0.778829	-0.226100	-0.000896
6	2.208548	-0.150644	-0.000555
6	2.907617	-0.108973	-1.215326
6	4.294320	-0.039836	-1.208789
6	4.986756	-0.011759	0.000068
6	4.294524	-0.054894	1.208590
6	2.907812	-0.124115	1.214507
1	2.361754	-0.140064	-2.152824
1	4.836635	-0.011927	-2.148039
1	6.070757	0.040033	0.000292
1	4.836984	-0.038684	2.148027
1	2.362100	-0.166826	2.151637
1	-0.790817	1.816468	0.006402
1	-1.966647	2.667705	-0.821788
1	-1.967470	2.662902	0.838481

Frequencies
11.1606998
46.2398987
49.9832993
131.891098
135.261993
167.709503
220.052994
248.096405
344.444611

347.502014
396.605011
411.516693
444.637512
484.670410
523.490906
544.311584
564.191406
571.291382
588.133606
641.484314
708.294006
710.660400
722.232727
785.223328
786.003906
800.126892
874.611572
876.617126
903.896973
962.878723
990.850220
1004.18140
1027.97729
1039.75415
1042.90369
1045.46741
1068.99634
1079.06738
1116.32166
1127.95886
1138.51575
1191.10693
1210.83228
1212.84082
1221.95337
1228.70093
1315.34753
1345.21399
1357.12915
1371.66394
1375.21936
1506.48376
1514.98254
1540.73145
1549.54297
1567.51355
1655.05078

1665.30725
1680.11182
1691.11243
1691.56335
1717.43835
2353.61792
3225.08325
3226.96167
3231.73291
3237.75439
3245.47168
3246.78320
3253.60913
3256.82349
3264.18481
3282.99951
3476.91284
3535.32764

IIa (first isomer)

6	3.283339	0.305059	-1.104985
6	2.064502	0.021645	-0.481439
6	2.046983	-0.423749	0.846728
6	3.238191	-0.580380	1.538612
6	4.452718	-0.295075	0.913483
6	4.474550	0.146168	-0.405956
6	0.822364	0.203880	-1.250573
6	-0.399436	0.027442	-0.835814
6	-1.656965	-0.151586	-0.416713
6	-2.397027	0.957860	0.187524
6	-3.729296	0.722876	0.610727
6	-4.287153	-0.516339	0.452309
6	-3.584479	-1.617148	-0.131009
6	-2.310371	-1.442356	-0.552641
1	1.103027	-0.653906	1.334362
1	3.300686	0.647061	-2.136044
1	3.223799	-0.928298	2.566171
1	5.382978	-0.420382	1.457824
1	5.418514	0.365365	-0.893430
1	0.922276	0.520832	-2.291831
1	-1.739570	-2.247318	-1.002242
1	-4.079372	-2.575897	-0.229636
1	-5.309259	-0.670779	0.785059
1	-4.296730	1.531876	1.059003
7	-1.831246	2.148905	0.334642
1	-2.335691	2.919706	0.749531
1	-0.879449	2.319623	0.037229

Frequencies
31.0580997
39.1110992
46.1012993
104.525902
126.455299
215.352005
265.618011
295.159790
358.987396
412.095306
414.967285
456.044495
479.773712
495.410400
517.326294
529.798218
557.180115
583.419617
602.781311
636.016479
662.899597
708.447693
741.639709
761.557190
776.523071
794.387573
843.099792
866.720703
876.788879
883.682678
888.545898
961.408630
1002.96301
1016.26593
1027.58984
1027.73303
1040.53613
1050.10889
1067.28210
1069.55786
1131.92786
1170.18188
1204.72205
1212.20020
1217.02625
1228.15503

1233.74341
1276.81702
1345.12891
1379.20569
1387.61499
1404.51648
1476.90234
1499.36047
1524.03577
1554.45447
1560.38928
1609.53027
1674.50781
1690.20850
1701.05286
1732.29602
1999.05518
3155.02979
3208.87866
3219.95215
3232.99438
3233.64941
3244.11133
3245.62036
3251.87183
3253.23071
3267.25439
3610.70483
3730.33960

IIa (second isomer)

6	3.283339	0.305059	-1.104985
6	2.064502	0.021645	-0.481439
6	2.046983	-0.423749	0.846728
6	3.238191	-0.580380	1.538612
6	4.452718	-0.295075	0.913483
6	4.474550	0.146168	-0.405956
6	0.822364	0.203880	-1.250573
6	-0.399436	0.027442	-0.835814
6	-1.656965	-0.151586	-0.416713
6	-2.397027	0.957860	0.187524
6	-3.729296	0.722876	0.610727
6	-4.287153	-0.516339	0.452309
6	-3.584479	-1.617148	-0.131009
6	-2.310371	-1.442356	-0.552641
1	1.103027	-0.653906	1.334362
1	3.300686	0.647061	-2.136044
1	3.223799	-0.928298	2.566171

1	5.382978	-0.420382	1.457824
1	5.418514	0.365365	-0.893430
1	0.922276	0.520832	-2.291831
1	-1.739570	-2.247318	-1.002242
1	-4.079372	-2.575897	-0.229636
1	-5.309259	-0.670779	0.785059
1	-4.296730	1.531876	1.059003
7	-1.831246	2.148905	0.334642
1	-2.335691	2.919706	0.749531
1	-0.879449	2.319623	0.037229

Frequencies
31.0580997
39.1110992
46.1012993
104.525902
126.455299
215.352005
265.618011
295.159790
358.987396
412.095306
414.967285
456.044495
479.773712
495.410400
517.326294
529.798218
557.180115
583.419617
602.781311
636.016479
662.899597
708.447693
741.639709
761.557190
776.523071
794.387573
843.099792
866.720703
876.788879
883.682678
888.545898
961.408630
1002.96301
1016.26593
1027.58984
1027.73303

1040.53613
1050.10889
1067.28210
1069.55786
1131.92786
1170.18188
1204.72205
1212.20020
1217.02625
1228.15503
1233.74341
1276.81702
1345.12891
1379.20569
1387.61499
1404.51648
1476.90234
1499.36047
1524.03577
1554.45447
1560.38928
1609.53027
1674.50781
1690.20850
1701.05286
1732.29602
1999.05518
3155.02979
3208.87866
3219.95215
3232.99438
3233.64941
3244.11133
3245.62036
3251.87183
3253.23071
3267.25439
3610.70483
3730.33960

IIa (third isomer)

6	-2.398797	0.957702	-0.183983
6	-1.657981	-0.151369	0.419884
6	-2.309886	-1.443024	0.553894
6	-3.582128	-1.620106	0.127408
6	-4.284502	-0.520325	-0.458036
6	-3.728653	0.720505	-0.612716
6	-0.399393	0.027856	0.836531

6	0.823028	0.205294	1.249491
6	2.064910	0.023330	0.480310
6	3.284584	0.296525	1.106730
6	4.475653	0.136626	0.407595
6	4.452810	-0.295314	-0.914880
6	3.237363	-0.569706	-1.543191
6	2.046381	-0.412028	-0.851201
1	-1.739289	-2.246769	1.005784
1	1.101630	-0.632781	-1.341541
1	3.302686	0.631561	2.140030
1	3.222123	-0.909707	-2.573377
1	5.382954	-0.421484	-1.459246
1	5.420278	0.347809	0.897325
1	0.922981	0.523072	2.290618
7	-1.835885	2.150792	-0.324295
1	-4.296485	1.529087	-1.061225
1	-5.305034	-0.676544	-0.794756
1	-4.075850	-2.579679	0.224030
1	-2.339307	2.920685	-0.742153
1	-0.885834	2.323420	-0.022440

Frequencies

30.9493999
39.0191002
45.9929008
104.612701
126.640198
215.487000
265.781006
295.510315
358.882202
412.284088
415.321991
455.918488
479.748901
495.553009
517.280701
530.171509
557.294373
583.361511
602.674377
635.993774
663.072205
708.387329
741.413818
761.797119
776.412292
794.419189

843.219299
866.645813
876.900818
883.663330
888.659607
961.384399
1002.93390
1016.17493
1027.63965
1027.82886
1040.55969
1050.06006
1067.52612
1069.57263
1131.87585
1170.16113
1204.78577
1212.19531
1217.10046
1228.04688
1233.72156
1276.93396
1345.15833
1379.21228
1387.70581
1404.51746
1476.91895
1499.34766
1524.00354
1554.69849
1560.44556
1609.29321
1674.53528
1690.21362
1701.12500
1732.53821
1998.01038
3153.81372
3209.47998
3220.31860
3232.99414
3233.72803
3244.33984
3245.62842
3252.22095
3253.33325
3267.35986
3610.68774

3730.08032

IIIa (first isomer)

6	1.689394	0.092360	-0.086577
6	1.632916	-1.282979	0.175534
6	2.797815	-1.999219	0.383773
6	4.028018	-1.339326	0.327022
6	4.091967	0.027280	0.098606
6	2.923345	0.771083	-0.092688
1	0.673309	-1.792721	0.177906
1	2.755987	-3.066341	0.570924
1	4.946901	-1.896645	0.477486
1	5.052940	0.533276	0.087905
7	2.940785	2.152320	-0.340420
6	0.499365	0.868963	-0.452612
6	-0.747469	0.589659	-0.265217
1	0.703882	1.816732	-0.971923
6	-2.068654	0.282819	-0.117333
6	-2.732381	-0.495088	-1.115729
6	-4.064503	-0.807553	-0.957529
6	-4.746308	-0.359387	0.182172
6	-4.115613	0.403314	1.175087
6	-2.785137	0.730210	1.034813
1	-2.172967	-0.824930	-1.984909
1	-4.584782	-1.394950	-1.705468
1	-5.796331	-0.610685	0.299699
1	-4.674873	0.732446	2.043431
1	-2.265832	1.319703	1.782899
1	2.488932	2.707546	0.377419
1	3.870808	2.517515	-0.508370

Frequencies

35.4943008
38.6948013
53.8088989
122.671501
138.836594
199.100800
227.858093
270.824310
333.416992
374.794098
394.887299
447.457397
462.298401
469.167389
499.170197
536.901978

554.649597
580.074402
624.980286
658.434509
675.061218
726.784119
762.090698
781.722290
803.654785
811.528198
833.803284
866.560120
876.388184
890.656982
905.726624
976.974670
1012.36963
1017.84259
1025.23169
1028.67676
1051.64893
1066.21704
1081.12024
1111.19934
1133.45447
1155.36975
1182.31616
1213.43396
1213.90039
1220.36743
1229.60645
1319.30542
1344.79004
1346.96472
1361.45593
1382.60144
1411.07581
1495.60034
1521.84497
1529.46497
1559.72302
1626.35779
1661.25671
1673.45996
1689.63867
1716.27026
1955.92993
3082.32104

3215.75488
3223.41919
3237.18701
3240.48560
3247.86914
3250.02759
3254.75952
3261.16016
3264.30127
3579.17578
3671.70410

IIIa (the second isomer collapses into the below reported cyclized structure)

6	2.549875	-1.226103	0.211274
6	1.781635	-0.077416	-0.026197
6	2.431084	1.148156	-0.225271
6	3.818351	1.221827	-0.194026
6	4.572247	0.074667	0.032495
6	3.934405	-1.148008	0.233847
6	0.327513	-0.192508	-0.063114
6	-0.528113	-1.219523	-0.182034
6	-1.909437	-0.756688	-0.099413
6	-1.897213	0.623576	0.072321
6	-3.020162	1.412417	0.185367
6	-4.243034	0.739052	0.123903
6	-4.294183	-0.645818	-0.047221
6	-3.134300	-1.411453	-0.162294
1	-3.187623	-2.486203	-0.296737
7	-0.486573	1.060142	0.092147
1	-2.974814	2.488664	0.317008
1	-5.163724	1.304985	0.210831
1	-5.260264	-1.136891	-0.092098
1	-0.228827	-2.248659	-0.328637
1	1.875191	2.062530	-0.422909
1	4.309021	2.176115	-0.352613
1	5.655206	0.132991	0.055815
1	4.518324	-2.043060	0.419799
1	2.063202	-2.178678	0.395511
1	-0.293083	1.714556	-0.674607
1	-0.238606	1.539580	0.965789

Frequencies
14.9844999
60.9944000
108.156303
169.868301
220.093399
280.681610

300.610504
329.093597
407.461609
422.454010
432.954102
509.316315
537.933472
561.544495
572.627197
613.446411
636.595825
683.088989
706.592590
731.186890
774.766296
782.971313
800.315674
852.386230
877.868591
891.701904
895.940979
913.075378
942.107727
981.614685
987.879211
997.875671
999.690125
1025.49158
1039.45874
1041.69373
1059.69629
1075.19104
1135.06750
1138.33496
1192.59119
1208.68726
1214.74634
1234.16089
1238.90344
1249.27673
1279.81018
1293.67847
1309.38574
1356.91833
1367.98914
1388.15796
1409.11475
1510.53223

1527.30396
1544.68835
1561.35632
1654.28906
1668.88293
1681.00867
1694.25916
1707.47205
1738.12378
3192.82788
3229.19458
3236.52905
3238.49438
3242.57593
3247.25806
3252.42310
3256.69409
3260.15942
3291.67529
3446.06128
3508.82202

TS from Ia + 1a to Va

6	2.189060	-3.171437	-0.525289
6	2.199658	-1.931189	0.145411
6	2.353906	-1.939639	1.547537
6	2.467858	-3.140756	2.246883
6	2.467312	-4.354761	1.568087
6	2.339673	-4.358809	0.178783
6	2.002660	-0.679352	-0.546820
6	0.996073	-0.248678	-1.201504
6	-0.364369	-0.648946	-1.556184
6	-1.177945	-1.191053	-0.554736
6	-2.455113	-1.669096	-0.785534
6	-2.966232	-1.606625	-2.077306
6	-2.194998	-1.058911	-3.098894
6	-0.912473	-0.585148	-2.841922
1	2.417650	-0.990602	2.074620
7	1.184873	1.435031	-1.824222
6	1.979342	2.211578	-0.917788
6	1.394270	2.639989	0.285405
6	2.175579	3.357024	1.196507
6	3.514201	3.612213	0.922959
6	4.087609	3.145463	-0.255432
6	3.320389	2.437774	-1.177036
6	0.033623	2.301624	0.566473
6	-1.118463	1.970519	0.755820

6	-2.450451	1.489772	0.975769
6	-3.390179	1.508614	-0.062897
6	-4.653737	0.965018	0.132820
6	-4.989550	0.390578	1.356569
6	-4.061539	0.373393	2.397342
6	-2.798234	0.924869	2.214061
1	1.723612	3.704959	2.119025
1	4.112448	4.168292	1.636460
1	5.136605	3.328117	-0.461652
1	3.770000	2.064197	-2.092001
1	-3.119800	1.940128	-1.021010
1	-5.378551	0.987985	-0.674450
1	-5.978173	-0.031494	1.505616
1	-4.329755	-0.052151	3.359238
1	-2.081968	0.943969	3.031460
1	1.628458	1.372343	-2.738768
1	0.250868	1.832479	-1.937962
7	-0.647288	-1.174729	0.816175
1	-3.061026	-2.056824	0.028752
1	-3.964624	-1.977495	-2.279894
1	-2.591520	-1.009885	-4.107656
1	-0.313655	-0.189416	-3.657150
1	2.075309	-3.186523	-1.604990
1	2.344278	-5.300613	-0.361924
1	2.578171	-5.287363	2.111180
1	2.586442	-3.120186	3.326372
1	-1.391757	-1.312004	1.504996
1	0.113830	-1.864024	0.969913
1	-0.217533	-0.250176	0.991652

Freq.

20.1704006
26.7334995
37.6464005
43.1525993
44.2391014
53.2179985
62.2103004
69.4319992
98.7221985
103.373199
118.731201
135.452301
153.031296
186.286896
191.161499
215.167603
254.175705

265.153107
285.582397
329.720001
338.715088
369.707001
381.649689
403.515900
410.742096
414.045410
414.986206
441.642303
455.072906
484.412903
488.579803
515.040710
519.088013
533.284973
541.226624
556.389282
571.271790
577.661804
579.430786
597.729187
617.720276
637.093018
640.182678
641.301575
708.366211
716.910217
721.812988
734.283630
742.576416
747.328613
762.648499
782.185486
783.943726
786.870728
789.260620
814.160889
833.020874
873.182312
877.715881
881.941528
894.327515
895.633911
910.574524
927.642029
955.932495

971.582275
980.011108
994.532776
1001.93091
1016.76642
1023.79010
1025.74048
1027.41858
1028.25134
1035.47058
1067.41846
1068.13391
1076.00183
1084.07166
1088.06860
1115.45056
1120.04785
1123.99036
1126.69263
1147.77649
1159.33630
1188.39380
1199.10413
1202.55261
1203.78357
1208.50464
1213.80945
1214.61694
1222.03699
1224.86133
1237.77295
1265.67395
1305.92126
1320.95740
1331.76587
1334.03809
1339.87292
1355.11194
1369.36646
1372.50220
1373.86035
1385.66064
1496.73059
1504.71558
1516.25732
1520.95142
1542.92798
1551.05078

1554.27051
1569.02539
1570.68774
1647.07507
1652.15466
1661.45349
1665.73059
1669.88696
1674.44543
1677.62634
1685.15430
1691.14050
1707.58936
1718.33325
1872.44312
2354.72339
3203.07764
3213.58228
3217.74292
3218.77124
3221.07129
3224.43311
3226.75708
3227.04419
3233.20459
3234.64893
3237.83984
3241.09399
3242.47583
3245.90161
3247.65454
3250.74780
3256.01147
3257.17334
3269.60522
3349.02393
3500.25024
3533.66406
3603.28394

TS from Ia + 1a to IVa

6	3.766013	-1.819615	0.915678
6	3.736979	-0.918827	-0.154342
6	4.939887	-0.705778	-0.840214
6	6.134063	-1.327029	-0.521204
6	6.129917	-2.217249	0.548801
6	4.952505	-2.458063	1.258311
6	2.583357	-0.162056	-0.648166

6	1.393434	-0.148660	-0.219936
6	0.299328	-0.506763	0.637703
6	0.030479	0.253393	1.785430
6	-1.026977	-0.102494	2.612787
6	-1.815012	-1.207634	2.301874
6	-1.545511	-1.972616	1.167529
6	-0.494609	-1.623719	0.332990
7	4.791816	0.254435	-1.938738
7	0.302905	1.243558	-1.432809
6	-0.028048	2.418079	-0.719571
6	-1.315769	2.558965	-0.168438
6	-1.599684	3.693556	0.603476
6	-0.629528	4.658905	0.829407
6	0.644708	4.504095	0.284812
6	0.945106	3.387053	-0.483516
6	-2.291170	1.539716	-0.383302
6	-3.055788	0.615732	-0.554551
6	-3.936404	-0.503111	-0.682573
6	-3.713835	-1.471549	-1.670762
6	-4.535852	-2.588505	-1.750637
6	-5.585340	-2.750353	-0.848750
6	-5.814501	-1.788021	0.132441
6	-4.996731	-0.668578	0.218940
1	-2.594871	3.802507	1.020948
1	-0.865230	5.533010	1.426783
1	1.406313	5.257420	0.458044
1	1.939690	3.257380	-0.901366
1	-2.894848	-1.341637	-2.371804
1	-4.360310	-3.333370	-2.520511
1	-6.227279	-3.623128	-0.913785
1	-6.635029	-1.909914	0.832424
1	-5.166999	0.081929	0.983962
1	0.979653	1.389607	-2.173569
1	-0.522591	0.760338	-1.782469
1	0.645226	1.118614	2.010988
1	-1.240478	0.487512	3.497971
1	-2.647677	-1.475886	2.944347
1	-2.170436	-2.825518	0.924370
1	-0.282177	-2.204551	-0.559450
1	2.855229	-2.012927	1.473212
1	4.963144	-3.154227	2.090550
1	7.047415	-2.723251	0.828463
1	7.043995	-1.131545	-1.080530
1	3.709468	0.467548	-1.808735
1	5.349202	1.103264	-1.822438
1	4.979443	-0.142348	-2.861863

Freq

26.7334995
37.6464005
43.1525993
44.2391014
53.2179985
62.2103004
69.4319992
98.7221985
103.373199
118.731201
135.452301
153.031296
186.286896
191.161499
215.167603
254.175705
265.153107
285.582397
329.720001
338.715088
369.707001
381.649689
403.515900
410.742096
414.045410
414.986206
441.642303
455.072906
484.412903
488.579803
515.040710
519.088013
533.284973
541.226624
556.389282
571.271790
577.661804
579.430786
597.729187
617.720276
637.093018
640.182678
641.301575
708.366211
716.910217
721.812988
734.283630
742.576416

747.328613
762.648499
782.185486
783.943726
786.870728
789.260620
814.160889
833.020874
873.182312
877.715881
881.941528
894.327515
895.633911
910.574524
927.642029
955.932495
971.582275
980.011108
994.532776
1001.93091
1016.76642
1023.79010
1025.74048
1027.41858
1028.25134
1035.47058
1067.41846
1068.13391
1076.00183
1084.07166
1088.06860
1115.45056
1120.04785
1123.99036
1126.69263
1147.77649
1159.33630
1188.39380
1199.10413
1202.55261
1203.78357
1208.50464
1213.80945
1214.61694
1222.03699
1224.86133
1237.77295
1265.67395

1305.92126
1320.95740
1331.76587
1334.03809
1339.87292
1355.11194
1369.36646
1372.50220
1373.86035
1385.66064
1496.73059
1504.71558
1516.25732
1520.95142
1542.92798
1551.05078
1554.27051
1569.02539
1570.68774
1647.07507
1652.15466
1661.45349
1665.73059
1669.88696
1674.44543
1677.62634
1685.15430
1691.14050
1707.58936
1718.33325
1872.44312
2354.72339
3203.07764
3213.58228
3217.74292
3218.77124
3221.07129
3224.43311
3226.75708
3227.04419
3233.20459
3234.64893
3237.83984
3241.09399
3242.47583
3245.90161
3247.65454
3250.74780

3256.01147
3257.17334
3269.60522
3349.02393
3500.25024
3533.66406
3603.28394

Va

6	-0.434062	-2.132719	-0.848078
6	-1.760238	-2.335820	-0.449349
6	-2.128841	-3.632003	-0.066906
6	-1.195311	-4.659912	-0.089719
6	0.119549	-4.421325	-0.487188
6	0.510074	-3.142483	-0.869270
6	-2.667598	-1.234519	-0.437570
6	-3.364029	-0.240323	-0.442089
6	-4.215439	0.907745	-0.421873
6	-5.587581	0.755745	-0.176857
6	-6.410992	1.873392	-0.152488
6	-5.877934	3.142098	-0.370064
6	-4.514932	3.297537	-0.613702
6	-3.683198	2.186548	-0.640574
7	-0.052653	-0.766546	-1.263420
1	-3.149519	-3.817035	0.247891
1	-1.494907	-5.658407	0.209795
1	0.842998	-5.228877	-0.496875
1	1.533822	-2.933693	-1.164667
1	-2.620386	2.306173	-0.832353
1	-4.100763	4.285815	-0.784045
1	-6.526271	4.012079	-0.350595
1	-7.472727	1.754146	0.036237
1	-5.996371	-0.235035	-0.008866
1	-0.884603	-0.175838	-1.096414
6	1.145195	-0.193364	-0.586657
1	0.098280	-0.739705	-2.277253
6	2.166211	0.147601	-1.385710
6	1.049701	-0.177657	0.885695
6	3.444302	0.782257	-1.046034
1	2.068881	-0.101058	-2.445425
6	-0.105789	0.266134	1.542564
6	-0.162810	0.272484	2.931655
6	0.930887	-0.159987	3.676346
6	2.082649	-0.602766	3.029689
6	2.142788	-0.617590	1.642387
1	-0.970520	0.616555	0.984050
1	-1.062195	0.618347	3.430216
1	0.885209	-0.153417	4.760500

1	2.934875	-0.944963	3.607466
1	3.038760	-0.962935	1.136511
6	4.580858	0.273007	-1.700464
6	5.841826	0.798844	-1.497619
6	5.979895	1.890140	-0.637109
6	4.876012	2.427416	-0.001598
6	3.586344	1.898874	-0.187558
1	4.455015	-0.567191	-2.379577
1	6.702566	0.378875	-2.005557
1	6.957941	2.328550	-0.465636
1	4.997289	3.285383	0.654294
7	2.507308	2.547940	0.387580
1	1.738948	1.983089	0.715778
1	2.755882	3.263251	1.056249

Frequencie
3.95090008
13.3373003
17.1366005
32.0451012
37.5527992
42.9950981
54.9124985
58.8983994
74.0179977
108.446800
114.824303
131.195999
147.653198
183.086197
202.956406
230.171204
238.311096
255.925095
272.308807
287.191498
308.123596
352.664307
369.253510
407.762512
411.095398
414.578491
423.128510
432.138611
461.516510
480.866913
505.196686
528.008972

531.857300
536.530579
545.064575
553.874390
571.679626
578.013306
580.053223
596.009094
604.831970
635.686096
642.201721
643.669800
680.936523
709.428284
712.702393
719.959900
734.712585
746.419678
762.748596
781.748474
783.289185
786.048706
796.328430
818.577087
831.163818
867.701599
873.967529
874.966003
882.327393
896.567078
907.644226
925.848389
953.596191
958.309204
972.225708
979.483887
990.278320
999.698975
1006.15417
1013.23969
1026.07178
1027.54004
1035.93689
1036.28149
1039.56030
1048.86206
1068.61206
1077.29834

1079.17358
1082.31335
1112.18188
1121.50232
1128.64404
1139.55273
1190.96289
1196.61121
1210.23242
1211.03735
1211.79895
1214.72302
1222.91699
1228.89514
1229.19385
1239.70496
1263.28284
1279.69556
1316.01685
1324.84204
1344.22156
1347.75049
1359.22217
1362.68115
1366.58862
1374.96545
1385.43835
1390.85413
1424.24512
1461.54712
1506.83093
1508.07739
1518.33606
1520.43555
1548.66602
1564.12964
1565.98425
1567.97534
1657.77209
1661.86450
1664.83716
1667.89746
1681.59619
1691.89539
1694.37146
1699.00061
1710.82959
1727.70190

1755.32104
2350.92798
3137.56177
3203.14893
3207.29468
3211.67920
3212.78418
3228.18066
3229.29956
3231.65649
3234.54321
3236.32275
3237.53931
3241.00293
3244.38770
3245.52588
3252.18604
3252.71338
3252.84985
3253.90771
3260.45239
3334.24365
3482.37231
3626.89771
3729.13696

IVa

6	5.070855	-0.248633	-0.968493
6	3.765891	-0.689363	-0.713031
6	3.579175	-1.941091	-0.110479
6	4.674858	-2.715925	0.245151
6	5.969037	-2.256705	0.008117
6	6.165672	-1.021487	-0.602012
6	2.657640	0.182258	-1.127556
6	1.408279	0.220077	-0.653783
6	0.756910	-0.518952	0.445605
6	1.216218	-0.427256	1.760942
6	0.522544	-1.056825	2.785805
6	-0.637937	-1.772732	2.497160
6	-1.096003	-1.883683	1.188637
6	-0.396160	-1.266041	0.155782
1	5.230088	0.711463	-1.453313
7	0.452348	1.176332	-1.288028
6	0.220841	2.412548	-0.512431
6	-1.042334	2.650262	0.040436
6	-1.219019	3.834606	0.770347
6	-0.171749	4.730563	0.931085
6	1.076220	4.465925	0.370333

6	1.275983	3.296972	-0.353871
6	-2.102533	1.709851	-0.120599
6	-2.960523	0.866428	-0.250995
6	-3.916065	-0.191222	-0.371419
6	-4.482276	-0.756066	0.780317
6	-5.369413	-1.819194	0.665294
6	-5.695574	-2.327706	-0.590548
6	-5.137735	-1.767953	-1.738112
6	-4.251947	-0.701805	-1.634163
1	-2.191235	4.035387	1.206050
1	-0.328652	5.641823	1.498082
1	1.894602	5.165882	0.497403
1	2.249817	3.074242	-0.779097
1	-3.822629	-0.252237	-2.524395
1	-5.400196	-2.157069	-2.716608
1	-6.389919	-3.157412	-0.675421
1	-5.809995	-2.251186	1.558015
1	-4.217846	-0.357920	1.754460
1	0.773162	1.417172	-2.229565
1	-0.430893	0.620128	-1.412973
1	2.916222	0.889896	-1.917074
1	2.112564	0.148049	1.970375
1	0.877170	-0.979592	3.807799
1	-1.186995	-2.258815	3.297279
1	-1.995880	-2.449959	0.965676
7	-0.833631	-1.295304	-1.201004
1	2.578744	-2.317866	0.070896
1	4.518226	-3.686093	0.705492
1	6.821807	-2.865989	0.289724
1	7.170459	-0.662644	-0.799341
1	-1.774195	-1.669839	-1.288026
1	-0.210574	-1.845727	-1.787335

Freq
16.3057995
25.1459999
30.5083008
36.6623001
43.5858994
46.0863991
51.8492012
72.6751022
88.2953033
108.506500
131.489395
150.353607
167.526703
177.185806

205.762695
220.313095
234.735901
265.217896
298.914093
330.126801
338.840302
372.662598
397.439301
414.085388
415.343994
416.793304
435.752899
443.605408
468.110596
496.232391
502.129791
518.078796
526.169922
534.023621
555.533386
566.400513
572.239197
580.297607
595.648621
596.808594
637.483704
639.650818
641.476379
682.255310
710.449219
714.745789
715.339111
736.916382
749.616089
767.656006
782.808777
786.400574
786.984985
799.070984
823.886780
850.672119
871.727905
873.593811
876.703125
878.376587
901.178101
903.595276

936.289917
947.994995
960.166016
967.055603
986.126709
987.230286
1001.33429
1003.52521
1006.42682
1026.00964
1027.03955
1027.25488
1032.17896
1034.66187
1035.66345
1036.26355
1068.52625
1073.24573
1084.93518
1085.54187
1124.24646
1124.81494
1132.73132
1135.61694
1194.21741
1195.84888
1209.08154
1209.50500
1211.14990
1212.01868
1221.40015
1224.56787
1227.77295
1233.04126
1254.48889
1265.41345
1291.03638
1318.27051
1341.52808
1344.11597
1358.52661
1363.39014
1367.01819
1372.03406
1373.62195
1380.21191
1399.43884
1455.77319

1505.07336
1513.50586
1515.46082
1524.01050
1551.16467
1556.37427
1565.24365
1571.00024
1662.58496
1663.41772
1665.95874
1671.13013
1687.79834
1691.43127
1695.62476
1700.60425
1710.44177
1719.57214
1779.09985
2380.31934
3068.61279
3160.10669
3207.51514
3221.96509
3222.70190
3227.09204
3228.10767
3229.77661
3231.64819
3236.72412
3236.85669
3238.93140
3240.63989
3244.09619
3246.81372
3251.23071
3252.07642
3252.38989
3253.07642
3259.63330
3504.26929
3536.96753
3629.61694

TS-to V!a

6	2.481328	-1.438367	0.066407
6	1.968644	-0.131300	-0.143164
6	2.847301	0.941012	-0.281745

6	4.218348	0.726291	-0.218476
6	4.722003	-0.553561	-0.011851
6	3.852759	-1.632401	0.131709
6	0.542237	-0.065390	-0.168721
6	-0.538429	-0.691188	-0.064941
6	-1.975297	-0.805311	-0.074591
6	-2.748816	0.295448	-0.470958
6	-4.132113	0.190100	-0.475870
6	-4.746589	-1.000669	-0.090038
6	-3.977923	-2.094583	0.301308
6	-2.592697	-2.001515	0.308937
7	1.531790	-2.513816	0.218235
8	0.013809	2.119728	-0.486235
1	-2.251139	1.210554	-0.773089
1	-4.735177	1.037866	-0.783994
1	-5.829081	-1.076466	-0.096126
1	-4.458521	-3.020081	0.599871
1	-1.990711	-2.852012	0.615621
1	2.444513	1.932919	-0.436701
1	4.895491	1.566071	-0.329587
1	5.792748	-0.717828	0.038300
1	4.243604	-2.632482	0.292983
6	-0.163443	2.916927	0.695660
1	0.260000	2.690291	-1.227104
1	0.254702	-1.697696	0.127651
1	1.588914	-3.190874	-0.541147
1	1.648355	-3.010622	1.099522
6	-1.196426	4.008432	0.490982
1	-0.487984	2.208311	1.461292
1	0.801440	3.335231	1.009060
1	-1.325108	4.579137	1.415478
1	-0.885706	4.710822	-0.290645
1	-2.164659	3.582198	0.212963

Frequencie
19.9834003
28.3068008
43.7005997
46.6940002
55.1510010
81.8342972
101.838699
119.912102
143.259201
157.130905
217.942001
234.911407
274.213715

277.429993
292.292786
347.324493
404.536804
415.540710
421.252808
443.529297
457.365295
489.252106
535.937317
541.274414
575.323486
585.778870
606.631409
635.692993
711.735596
716.315430
725.716492
746.920288
786.085999
787.831726
823.395874
826.316589
879.563904
881.076111
908.990601
912.317383
969.256470
989.970825
1010.79291
1013.23071
1028.42798
1036.45520
1042.61426
1069.47424
1078.17883
1083.60925
1098.58411
1128.51184
1129.69495
1144.38489
1156.65955
1194.92798
1211.40015
1213.46985
1229.11133
1235.23071
1262.21326

1294.27808
1299.92249
1335.04199
1344.10291
1363.56165
1378.74304
1385.68506
1438.90649
1464.20752
1497.95032
1520.77441
1523.44153
1526.18726
1527.62781
1551.56104
1554.45923
1564.89978
1647.82776
1668.51941
1677.83362
1689.88989
1702.46460
2103.68115
3071.51489
3074.83179
3143.53149
3162.44995
3171.70581
3220.58618
3229.01196
3231.04077
3240.85522
3244.53857
3249.91870
3255.24976
3257.25171
3284.63501
3530.46948
3615.00269
3819.11914

VIa

6	2.888562	-1.085863	1.242911
6	2.045069	-0.790103	0.166335
6	2.611669	-0.457825	-1.070157
6	3.993401	-0.391516	-1.216922
6	4.823741	-0.661268	-0.132525

6	4.268760	-1.012127	1.096319
6	0.582348	-0.912850	0.317191
6	-0.389200	-0.059047	-0.046499
8	-0.228644	1.255011	-0.449277
6	0.920607	2.019611	-0.011432
6	0.535956	3.484188	-0.036114
6	-1.820507	-0.472324	-0.060327
6	-2.162534	-1.802082	-0.346126
6	-3.481383	-2.236350	-0.340680
6	-4.518967	-1.346402	-0.078041
6	-4.222149	-0.014029	0.176796
6	-2.896267	0.390902	0.179513
1	-1.373417	-2.498529	-0.606024
1	1.963648	-0.272574	-1.922946
1	4.421454	-0.139655	-2.182073
1	5.901720	-0.611439	-0.247541
1	4.913165	-1.235088	1.940649
1	2.459340	-1.368087	2.200609
7	-2.610371	1.815808	0.455561
1	-5.018313	0.699856	0.371809
1	-5.550636	-1.679347	-0.086784
1	-3.701398	-3.274360	-0.565419
1	0.236503	-1.847141	0.752325
1	1.751607	1.822465	-0.688986
1	1.207344	1.683750	0.989823
1	1.413154	4.094042	0.196113
1	0.176758	3.777240	-1.027186
1	-0.228958	3.724702	0.712806
1	-3.270393	2.436487	-0.019822
1	-2.634138	2.031630	1.456657
1	-1.640340	1.987817	0.089808

Frequencie

30.6053009
37.0932007
41.2518997
54.5227013
66.9561996
79.9139023
139.202805
147.623505
205.628006
219.512299
251.835297
263.154999
274.404602
311.205109
318.738586

375.605286
411.603394
412.678497
456.644989
488.873810
505.545502
551.797424
562.240784
579.991821
599.158997
637.043274
640.440491
705.609009
706.778687
718.473083
750.466187
778.335876
784.706787
788.346680
824.952576
842.723572
847.977722
869.897278
889.352112
897.421570
931.930725
954.876526
972.854919
985.845093
1004.10199
1013.80261
1017.81451
1026.22070
1041.83215
1070.11536
1082.90479
1109.62219
1133.83716
1139.04175
1184.84058
1187.85510
1207.35071
1212.67078
1213.27490
1232.30786
1247.97644
1304.91650
1325.55505

1349.12061
1360.68201
1364.96106
1375.58936
1380.44177
1424.10950
1458.34656
1461.76733
1507.85449
1520.37012
1525.75134
1529.98938
1544.22388
1554.36755
1562.95496
1663.43884
1666.36450
1689.05859
1692.66895
1719.11902
1767.88562
3082.42358
3148.16187
3165.93896
3174.27588
3180.64575
3189.90894
3217.13672
3229.29956
3229.79175
3232.02808
3237.49365
3238.89478
3246.63501
3252.19946
3254.90381
3593.71167
3655.19507
3687.42041

Ts-to VIIa

6	-3.064358	-1.278884	-0.213060
6	-2.053570	-0.347210	0.072144
6	-2.378405	0.965587	0.404050
6	-3.723817	1.321606	0.440651
6	-4.722356	0.391148	0.153991
6	-4.401314	-0.925624	-0.176802

6	-0.729430	-0.941230	-0.031092
6	0.511672	-0.844348	0.033299
6	1.913316	-0.917068	0.040574
6	2.631104	-0.690270	-1.151327
6	4.015198	-0.747768	-1.137658
6	4.685328	-1.028267	0.054223
6	3.981660	-1.254529	1.239068
6	2.597954	-1.201655	1.239262
7	-2.535767	-2.588758	-0.530118
8	0.637585	1.867487	0.676931
1	2.090610	-0.474507	-2.067018
1	-1.585655	1.671487	0.630547
1	-3.998937	2.338960	0.698145
1	-5.764411	0.690865	0.189016
1	-5.179470	-1.649089	-0.397861
1	2.032539	-1.373017	2.149163
1	4.517525	-1.473529	2.156178
1	5.769897	-1.073088	0.059873
1	4.575660	-0.577554	-2.050409
1	1.177430	2.159621	1.422209
6	1.154906	2.481922	-0.499412
1	-1.197439	-2.128248	-0.354073
1	-2.811115	-3.316200	0.128310
1	-2.743858	-2.901548	-1.477367
6	1.176037	3.997834	-0.404733
1	2.159191	2.089486	-0.718752
1	0.490916	2.155006	-1.305756
1	1.538485	4.436287	-1.339551
1	0.172940	4.385637	-0.205148
1	1.841833	4.333382	0.398819

Frequencie

10.3504000
32.0833015
37.0141983
42.0872002
51.9585991
59.8014984
81.0795975
98.3243027
119.019798
140.460999
164.343796
227.925507
259.737396
270.181091
307.546387
364.802002

393.616089
407.781586
443.368286
446.491913
458.262787
466.582886
522.860779
562.300781
580.310608
583.049316
618.975403
637.637390
669.591309
697.778198
747.858215
750.145691
796.243896
798.175171
827.257324
834.595581
875.297302
884.035522
914.896484
926.632874
983.834473
1013.70050
1014.61438
1017.28363
1026.44055
1050.55664
1056.91077
1063.88208
1070.34131
1084.99146
1113.79675
1123.31018
1129.89905
1164.55090
1172.40442
1191.41846
1212.76440
1214.90894
1225.92407
1263.02197
1300.91785
1304.63647
1324.41821
1345.55249

1351.79102
1362.46875
1381.85828
1400.61768
1439.11865
1463.18018
1503.67944
1524.51807
1526.98523
1529.88281
1538.70654
1554.68445
1556.25964
1646.90942
1653.11743
1662.95544
1681.09534
1699.36157
1756.00281
2148.76880
3039.10498
3072.04761
3120.87695
3152.71729
3167.92627
3232.30322
3232.57788
3238.70435
3240.55103
3244.19849
3246.10522
3253.92700
3254.05493
3258.43604
3527.96460
3614.69116
3835.47144

VIIa

6	2.554433	-1.568966	-0.433206
6	2.036993	-0.385815	0.103795
6	2.910397	0.547453	0.675880
6	4.274357	0.290155	0.727187
6	4.780734	-0.897149	0.203044
6	3.919590	-1.824077	-0.378151
6	0.585643	-0.111984	0.077635
8	0.222848	1.215495	-0.049127
6	-0.372259	-1.043137	0.228568

6	-1.832892	-0.862354	0.113448
6	-2.559040	0.169066	0.716046
6	-3.933604	0.309575	0.592619
6	-4.639281	-0.622965	-0.156654
6	-3.957917	-1.682768	-0.750365
6	-2.581671	-1.801264	-0.609793
7	-1.825286	1.134188	1.555785
1	2.516031	1.471098	1.089838
1	-2.059784	-2.628896	-1.080108
1	-4.503284	-2.421456	-1.327795
1	-5.713775	-0.527071	-0.264402
1	-4.456535	1.128594	1.079965
1	1.891371	-2.279956	-0.917257
1	4.312986	-2.742504	-0.801356
1	5.846856	-1.095941	0.240915
1	4.943511	1.015129	1.179103
6	0.591036	1.877534	-1.285371
1	-1.009900	1.501675	0.995801
1	-2.423584	1.905370	1.859748
1	-1.438943	0.682708	2.391577
1	-0.039201	-2.063870	0.391317
6	0.114182	3.308881	-1.200541
1	1.676410	1.818268	-1.399784
1	0.115621	1.335045	-2.108860
1	0.386886	3.840414	-2.116094
1	-0.974966	3.358554	-1.098151
1	0.580309	3.826443	-0.356778

Frequencie

37.2639008
46.2661018
54.2258987
66.9384003
105.857597
125.691101
145.748093
161.264206
206.128494
234.584793
270.352997
273.736511
291.222198
312.143494
357.675293
374.754700
418.807495
422.045898
446.086304
476.452911

514.795471
532.172913
567.523376
603.583618
628.174927
639.902588
673.280518
723.839722
737.208191
745.814880
780.291321
789.233215
833.218323
851.441589
878.013489
894.370911
903.320679
907.173401
928.754089
960.467773
997.693176
1000.21320
1027.63428
1032.02832
1042.95020
1049.89026
1073.29358
1088.60522
1094.85486
1123.19507
1129.86584
1144.13135
1155.50781
1181.50415
1202.68689
1207.50842
1217.57166
1228.61182
1229.53418
1251.13989
1275.44409
1329.80347
1336.96362
1349.92139
1375.35388
1378.08496
1413.85095
1427.80054

1463.62329
1509.87280
1514.35254
1523.65442
1532.48877
1546.96667
1557.17896
1562.78857
1568.06104
1657.28992
1670.31604
1681.20447
1697.03357
1708.68567
1723.34058
1750.38843
3067.23218
3078.76660
3080.83643
3129.66602
3163.11572
3181.09937
3213.06177
3214.30396
3216.66968
3225.06641
3228.56519
3237.33521
3243.85791
3248.26904
3257.25366
3267.61792
3482.40918
3554.83008

TsOH

6	1.945862	-1.203577	0.019904
6	0.557067	-1.216536	-0.032629
6	-0.124999	-0.005890	-0.082537
6	0.556206	1.208472	-0.091418
6	1.943056	1.200225	-0.041568
6	2.656988	-0.001514	0.018080
16	-1.895852	-0.010507	-0.127307
8	-2.340963	-1.234999	-0.748840
8	-2.279666	-0.132953	1.449820
8	-2.364180	1.294000	-0.561814
1	0.004237	2.140173	-0.154655
1	2.483149	2.143212	-0.054937

6	4.163016	0.013824	0.068674
1	2.485573	-2.145596	0.057026
1	0.007638	-2.151691	-0.046735
1	-2.623180	0.730847	1.728234
1	4.573145	-0.997564	0.132034
1	4.578770	0.491574	-0.825179
1	4.519585	0.578745	0.936773

Frequencies

24.4405994
34.1104012
87.7325974
150.397202
171.605103
206.320694
290.361603
328.769501
354.416199
389.100311
418.624695
467.939087
480.920410
547.294678
560.688721
654.061707
672.326477
723.993225
811.954712
840.951477
848.293213
871.819397
991.791992
1001.72223
1029.37915
1047.65845
1083.95959
1131.77686
1159.29187
1169.59033
1217.01819
1231.62854
1255.69202
1349.39331
1358.97632
1430.99316
1452.07410
1465.14160
1520.96326

1530.44751
1563.75122
1668.80774
1696.45911
3071.65308
3140.25000
3164.35938
3216.00610
3221.00391
3248.62695
3252.53491
3800.67139

TsO-

6	1.918578	-1.199011	0.001279
6	0.527918	-1.194121	0.002865
6	-0.170459	0.010947	0.001627
6	0.539528	1.207195	0.000925
6	1.931255	1.197502	-0.001143
6	2.642605	-0.004229	-0.000614
16	-1.982699	0.002067	-0.001658
8	-2.328503	-0.720848	-1.240450
8	-2.327059	-0.725472	1.235412
8	-2.352757	1.431108	0.005650
1	-0.022944	2.135632	0.000894
1	2.476561	2.140774	-0.002408
6	4.152632	-0.003419	-0.001357
1	2.454051	-2.147426	0.001698
1	-0.028759	-2.126454	0.004707
1	4.551194	-1.023798	-0.000488
1	4.553127	0.509404	-0.884959
1	4.554163	0.511308	0.880689

Frequencies
16.0149994
91.3380966
162.364502
212.714294
277.334991
322.752899
355.114410
393.230988
418.248199
491.482513
548.005310
568.540710
580.184204
658.800781

682.698181
728.405823
826.969604
831.430908
867.756714
961.265076
994.872620
1021.41211
1037.73706
1059.50830
1079.16736
1145.23779
1152.11731
1219.32690
1251.58154
1279.18384
1282.49390
1338.48230
1355.23450
1444.53625
1457.34570
1522.70178
1533.83374
1564.35693
1664.50903
1698.08569
3048.44165
3110.17261
3132.76245
3176.94556
3181.41553
3233.05908
3241.95068

Ethanol

6	-1.219831	-0.219927	-0.000000
1	-1.287894	-0.857199	0.886751
6	0.091696	0.545213	-0.000000
1	-1.287895	-0.857199	-0.886751
1	-2.067345	0.473236	0.000000
1	0.142084	1.196421	-0.887883
1	0.142083	1.196421	0.887883
8	1.143803	-0.398491	0.000000
1	1.977355	0.084535	-0.000000

Frequencies
252.891907
301.278412
424.402191

836.127319
929.438171
1060.68945
1160.25427
1206.27466
1309.71106
1324.50586
1433.50024
1496.64563
1520.19775
1538.76526
1570.21216
3000.15186
3030.74219
3076.64111
3158.18115
3164.75708
3858.85522

Protonated ether

6	0.027051	-0.076856	0.040253
1	0.074731	-0.130165	1.132002
1	1.049634	-0.077532	-0.344032
1	-0.501694	-0.971698	-0.303886
6	-0.700197	1.188873	-0.329358
8	-0.714631	1.306308	-1.826269
1	-1.742762	1.213081	-0.003521
1	-0.178426	2.092259	-0.014859
6	-1.442271	2.474256	-2.427244
6	-1.376080	2.353850	-3.926806
1	-2.457625	2.451222	-2.024475
1	-0.894892	3.333181	-2.040094
1	-1.866484	3.232579	-4.356231
1	-0.343011	2.335135	-4.281489
1	-1.912831	1.472960	-4.293832
1	-1.002842	0.469309	-2.237155

Frequencies
65.4321976
96.1373978
175.497299
257.637787
265.871796
380.929993
414.538788
672.644287
817.639221
827.837097
850.644714

857.299194
988.854614
1023.08960
1093.57092
1155.32983
1173.98779
1222.08215
1294.18103
1338.61084
1374.10413
1439.81274
1454.70813
1463.17639
1469.50549
1518.82422
1523.87451
1526.98645
1529.20996
1531.57104
1546.54041
3085.23120
3085.39380
3131.52686
3132.34839
3166.69727
3166.84497
3188.35498
3188.53687
3217.29370
3217.89868
3709.20239

TS-C

6	-4.776431	-2.092771	8.989843
6	-4.143011	-1.997891	7.744049
6	-2.940004	-1.287438	7.653839
6	-2.401253	-0.656788	8.769666
6	-3.050398	-0.741447	9.997965
6	-4.235150	-1.466574	10.105387
6	-4.668734	-2.655837	6.535098
6	-5.945257	-2.817727	6.166853
8	-6.174305	-3.493368	4.960608
6	-6.600669	-2.079001	3.465963
6	-6.859951	-3.117852	2.422450
6	-7.185679	-2.281167	6.784510
6	-7.256755	-0.960400	7.241503

6	-8.451743	-0.433289	7.714103
6	-9.600450	-1.220794	7.725277
6	-9.547395	-2.537629	7.281906
6	-8.347801	-3.072027	6.818718
7	-8.258492	-4.412305	6.317707
8	-6.826577	-0.468603	2.385920
6	-5.603757	0.232716	2.031031
6	-4.823019	-0.607333	1.045802
1	-6.360947	-0.347811	7.223819
1	-8.488334	0.591867	8.067263
1	-10.538540	-0.814599	8.089216
1	-10.438843	-3.158763	7.307224
1	-7.739396	-5.014567	6.954550
1	-9.174219	-4.828380	6.179460
1	-6.988320	-4.048426	5.122390
1	-5.596124	-1.768011	3.717379
1	-7.393560	-1.741250	4.120578
1	-5.973419	-3.734856	2.268319
1	-7.126082	-2.639687	1.477463
1	-7.696813	-3.754284	2.718228
1	-7.409129	0.142696	2.860826
1	-5.029557	0.432681	2.943482
1	-5.899788	1.186385	1.586067
1	-4.497814	-1.555951	1.483756
1	-3.927700	-0.059873	0.738887
1	-5.421182	-0.815078	0.154883
1	-3.929461	-3.072836	5.852910
1	-2.422795	-1.228629	6.699305
1	-1.469867	-0.106431	8.681602
1	-2.627735	-0.256467	10.872096
1	-4.733970	-1.554331	11.065445
1	-5.689263	-2.671655	9.088773

Frequencies

8.27499962
18.1201000
29.5510006
35.9976997
53.1911011
56.2299004
69.2294006
76.4414978
98.9570007
112.932999
142.689194
161.780106
186.760406
201.008408
225.611603

266.334198
281.219391
291.463287
323.439911
334.135803
364.204498
381.161896
392.828186
421.395111
424.690887
435.947693
446.456207
487.594299
512.757324
529.486023
574.976379
607.533813
625.338013
638.539490
641.160828
661.783020
723.208923
732.111572
764.677124
784.797424
794.149414
829.725830
837.736084
849.456299
879.205994
883.117615
889.434692
898.790527
925.633789
946.325684
963.838501
989.437378
1001.96619
1012.74237
1020.96417
1024.07617
1027.33313
1031.41455
1061.02502
1072.58069
1078.37354
1084.10925
1092.10742

1101.88477
1128.48792
1137.53955
1138.82092
1190.18042
1195.61267
1207.02661
1209.57251
1211.98328
1222.42383
1233.91223
1258.07239
1276.64111
1284.30481
1319.29065
1334.59399
1336.80615
1351.28064
1366.50244
1378.43457
1384.01135
1434.75452
1439.82666
1477.93250
1486.50781
1494.43933
1512.11023
1516.17712
1519.85449
1522.58984
1530.51453
1535.65601
1551.54565
1554.51086
1566.82434
1663.92224
1670.75977
1687.34595
1698.00049
1711.09888
1776.01636
3080.79834
3087.84985
3105.43335
3146.85229
3172.17261
3182.52100
3187.55054

3191.75854
3202.53540
3207.57520
3214.71924
3220.22168
3222.46338
3229.13843
3230.42871
3231.30957
3239.93921
3240.22119
3248.23218
3250.85986
3347.63794
3534.42383
3632.80249
3808.08960

TS-C’

6	3.160901	-0.404757	-1.396984
6	2.633267	-0.367458	-0.099925
6	3.481385	-0.052804	0.968506
6	4.831050	0.195144	0.746028
6	5.349763	0.139313	-0.544253
6	4.510158	-0.159188	-1.615138
6	1.204865	-0.638040	0.157073
8	0.735627	-0.035860	1.309781
1	3.080943	-0.014445	1.975659
6	0.378132	-1.339001	-0.644049
6	-1.086491	-1.482319	-0.508823
6	-1.876705	-1.370438	-1.663682
6	-3.262119	-1.428195	-1.607460
6	-3.896019	-1.610684	-0.380048
6	-3.136661	-1.766957	0.772744
6	-1.743804	-1.725404	0.710959
6	-0.334135	1.656596	0.797049
6	-1.078902	1.755359	2.086066
8	-1.210693	3.186400	-0.144818
6	-2.124121	2.786809	-1.199001
6	-3.318855	2.100596	-0.576239
7	-0.953602	-1.912314	1.889563
1	-3.626596	-1.956096	1.724629
1	-4.978373	-1.657804	-0.321686
1	-3.846373	-1.333814	-2.516768
1	-1.381055	-1.223523	-2.619666
1	0.814798	-1.786007	-1.531713
1	2.513940	-0.607237	-2.245238
1	4.905942	-0.191330	-2.625168

1	6.403581	0.332867	-0.716920
1	5.480017	0.428071	1.584207
1	0.631706	2.133399	0.694396
1	-0.676004	1.040169	-0.021369
1	-0.402562	1.526946	2.913414
1	-1.931942	1.074024	2.104820
1	-1.438763	2.776744	2.226219
1	0.074338	-0.678524	1.749987
1	-0.531108	3.763238	-0.523579
1	-2.423163	3.694172	-1.730998
1	-1.600212	2.123552	-1.898385
1	-4.044063	1.862239	-1.359021
1	-3.043507	1.158177	-0.093086
1	-3.800518	2.754478	0.155544
1	-1.523990	-2.054585	2.717922
1	-0.331548	-2.714273	1.793229

Frequencies

25.3561993
38.4766998
39.5320015
53.6128006
65.1243973
66.8716965
92.8392029
100.638702
121.226097
145.276703
161.385895
180.858704
194.919998
226.781296
241.498596
273.985413
281.195190
306.604706
317.720703
327.711090
335.761597
371.910004
417.856506
434.841614
448.392914
459.379303
466.330902
497.265198
499.897705
553.692383
561.018127

604.822876
614.669495
637.860291
684.912781
691.916504
718.192322
750.728516
781.789429
790.802429
799.935913
831.153625
838.156982
856.458008
875.235229
882.463013
889.404785
893.691101
903.088623
961.044189
977.660278
993.658020
1004.10358
1018.02393
1020.24280
1026.73474
1034.67590
1057.47449
1065.20776
1071.30286
1084.48328
1088.72546
1089.34314
1122.92517
1128.37891
1135.65540
1169.44714
1190.77063
1198.14099
1209.46313
1211.49255
1222.55127
1233.72559
1250.21838
1268.62854
1285.99976
1312.64722
1316.18274
1333.65076

1340.16931
1354.33142
1372.60974
1381.03015
1392.08301
1429.96570
1439.16333
1476.87305
1492.01758
1509.06006
1509.56055
1515.50916
1516.82031
1529.32214
1534.46899
1552.31531
1557.92004
1562.31519
1579.36914
1665.64746
1671.75586
1682.73145
1697.00244
1706.36475
1736.01123
2749.75366
3074.53613
3088.76440
3098.84302
3140.72217
3173.09644
3181.13501
3184.80371
3193.43164
3210.22266
3215.02930
3218.10889
3226.00293
3232.33398
3235.72095
3236.34375
3236.86426
3245.67383
3249.62524
3252.48730
3361.14453
3529.89746
3630.34058

3810.02148

Product from TS (Figure 3B)

6	2.133452	-0.468804	0.056702
6	2.699227	0.216861	-1.029488
6	4.060848	0.509281	-1.047698
6	4.873062	0.115274	0.010273
6	4.323942	-0.585700	1.083097
6	2.966670	-0.878519	1.105920
1	2.086775	0.468805	-1.892727
1	4.487846	1.028658	-1.899644
1	5.934700	0.339075	-0.006241
1	4.957813	-0.907481	1.903114
1	2.543654	-1.424879	1.944402
6	0.690314	-0.766127	0.115935
6	-0.276554	0.117665	-0.166341
6	-1.724549	-0.179660	-0.120807
6	-2.196484	-1.453474	-0.463689
6	-3.547871	-1.766412	-0.404720
6	-4.480814	-0.806547	-0.020729
6	-4.049147	0.471933	0.308667
6	-2.693609	0.757871	0.253511
1	-1.486318	-2.196995	-0.808513
1	-3.877273	-2.762579	-0.679375
1	-5.537724	-1.045248	0.013257
1	-4.764521	1.233691	0.607684
7	-2.260195	2.122365	0.640140
8	-0.014411	1.438579	-0.497480
1	-1.287622	2.260749	0.276697
1	0.941706	1.599109	-0.405696
1	-2.254043	2.248201	1.657032
1	-2.868899	2.839879	0.236790
1	0.389637	-1.748025	0.468269ù

32.3465004
49.2661018
64.3330994
104.896500
128.870895
196.635406
230.325806
250.227493
264.661713
332.727509
393.657013
414.585114
424.649689
440.976288

462.363892
489.936401
523.289673
547.471802
570.451416
591.762512
636.521423
653.029175
673.649170
727.710815
735.780273
764.377930
787.900696
794.392029
848.345520
860.600403
880.404785
886.669128
905.752197
959.251770
990.704102
1001.77972
1025.61780
1036.12805
1040.62439
1053.18115
1072.37158
1090.71753
1097.90613
1128.29138
1131.00879
1166.22473
1209.26355
1213.38086
1220.23999
1229.19482
1254.21545
1277.86255
1324.11719
1344.88708
1360.13538
1374.12476
1386.75342
1398.86804
1508.70239
1515.41760
1555.52173
1563.77014

1582.51099
1657.93713
1667.51794
1683.32349
1694.41638
1711.64807
1723.36194
1764.96704
3121.30371
3197.39893
3216.58643
3220.02588
3230.56836
3232.83813
3239.52368
3243.25586
3250.45093
3254.27148
3261.87671
3481.74707
3548.11670
3708.63623

Product from TS-C' (Figure 3B)

6	-2.944845	1.081749	-0.004856
6	-2.079196	-0.016868	0.078048
6	-2.609239	-1.311301	0.054164
6	-3.980155	-1.501906	-0.064689
6	-4.834997	-0.405436	-0.151238
6	-4.316149	0.886024	-0.120370
6	-0.623993	0.196325	0.206502
8	-0.242913	1.270760	0.984462
6	0.321657	-0.552364	-0.386511
6	1.781844	-0.470109	-0.198874
6	2.519943	0.715564	-0.229969
6	3.893948	0.769956	-0.045973
6	4.587241	-0.414106	0.167184
6	3.894586	-1.622936	0.174270
6	2.519134	-1.647758	-0.011581
7	1.801358	1.973466	-0.521232
1	-1.948803	-2.166850	0.156805
1	1.988110	-2.594372	0.004061
1	4.431064	-2.552771	0.329477
1	5.660969	-0.391531	0.314969
1	4.425510	1.717781	-0.075697
1	-2.550661	2.095387	-0.015535
1	-4.978480	1.742226	-0.194368

1	-5.905684	-0.557768	-0.239194
1	-4.383817	-2.508916	-0.075844
1	-0.975545	1.538414	1.558174
1	2.425347	2.782385	-0.474126
1	1.382779	1.948183	-1.457498
1	1.015707	2.091499	0.158014
1	-0.031359	-1.365004	-1.012948

45.3053017
51.3650017
76.6466980
120.232498
156.230392
187.721207
252.921707
272.860291
285.930115
294.731812
345.265106
414.069794
428.939087
435.775909
447.775787
469.321991
505.336914
535.151306
560.459900
608.780029
635.683472
676.780518
678.551392
722.836182
732.786072
771.841675
781.149170
793.770081
846.767700
864.788879
876.279114
880.620178
908.250610
961.731628
984.297913
1005.34808
1026.01379
1037.29919
1039.90186
1055.89575

1071.10498
1085.87280
1090.54773
1135.62805
1143.32971
1159.30627
1211.38330
1212.55371
1217.70618
1233.75610
1259.47498
1272.29358
1319.99805
1349.08118
1359.98706
1376.06299
1380.86792
1396.46570
1509.71582
1516.43408
1559.16174
1561.41565
1571.83337
1663.25818
1669.86230
1672.40576
1694.63794
1702.75183
1735.47095
1766.03613
3140.58105
3202.54492
3213.56030
3227.33105
3233.46826
3234.93799
3237.89014
3244.06958
3247.44580
3252.89038
3259.54932
3474.72217
3559.31421
3807.95874

TS addition of water to Ia (Figure 3C)

6	2.114387	-0.174536	0.059613

6	2.812542	1.020364	-0.165252
6	4.199164	0.998972	-0.199601
6	4.888123	-0.198979	-0.012963
6	4.192275	-1.385030	0.210524
6	2.804794	-1.377880	0.247904
1	2.258970	1.941813	-0.308650
1	4.746694	1.919298	-0.373496
1	5.972852	-0.207422	-0.041138
1	4.731145	-2.314845	0.357198
1	2.257815	-2.298931	0.427107
6	0.673255	-0.158777	0.093504
6	-0.470185	0.354852	0.058722
6	-1.886563	0.231510	0.008664
6	-2.799001	1.275925	0.165243
6	-4.159671	1.003956	0.170710
6	-4.613910	-0.305584	0.044916
6	-3.708698	-1.352964	-0.102174
6	-2.342582	-1.106704	-0.135452
1	-2.423647	2.284953	0.272955
1	-4.867279	1.818036	0.282161
1	-5.677724	-0.516534	0.056933
1	-4.065861	-2.373108	-0.205494
7	-1.362916	-2.148271	-0.241656
8	-0.074642	2.727827	-0.070212
1	-0.235049	3.321095	-0.814864
1	0.045530	3.294366	0.702193
1	0.007689	-1.191893	0.126881
1	-1.613108	-2.963987	0.310867
1	-1.224057	-2.448318	-1.205514

33.3235016
44.7279015
61.4216003
72.8665009
106.642197
141.405594
163.425293
183.714905
213.965698
217.871994
243.633194
274.145905
319.382904
343.261108
401.603790
414.216095
415.179688
462.154388

503.542786
526.744873
537.460571
568.126892
584.948914
610.664795
639.374084
709.937988
723.828918
729.402527
784.123413
788.787781
825.995605
868.273315
878.328003
886.415283
914.141785
958.224487
971.187683
999.280884
1013.11121
1028.94019
1039.81531
1043.94690
1062.90466
1072.74170
1083.81653
1130.11462
1145.33411
1196.71021
1209.12195
1212.69031
1214.69446
1231.36182
1267.78394
1304.37292
1340.01477
1352.69873
1363.69104
1382.05615
1512.32605
1519.19617
1523.71753
1558.16101
1644.85376
1667.26965
1677.94995
1689.41895

1695.90881
1699.78406
1849.28845
2119.02979
3223.37915
3229.34961
3233.05298
3243.71460
3244.19336
3252.89453
3256.78638
3260.57886
3278.96460
3533.06006
3625.72827
3815.19019
3935.64575

TS addition of water to Ia (Figure 3C)

6	-2.811677	-0.268993	1.223160
6	-2.110478	-0.288057	0.000031
6	-2.812147	-0.261822	-1.222695
6	-4.195978	-0.219416	-1.214984
6	-4.882502	-0.202561	0.000824
6	-4.195523	-0.226598	1.216241
6	-0.709586	-0.328021	-0.000343
8	-0.652532	2.573460	-0.001427
1	-2.258319	-0.287940	2.156149
6	0.521531	-0.522188	-0.000762
6	1.888324	-0.023201	0.000322
6	2.312761	1.302729	0.002873
6	3.681669	1.555994	0.003560
6	4.606011	0.511534	0.001736
6	4.184657	-0.818456	-0.000850
6	2.824161	-1.069593	-0.001529
1	1.575164	2.099122	0.004145
1	4.034271	2.582038	0.005542
1	5.668216	0.732062	0.002316
1	4.905177	-1.630054	-0.002281
7	2.196467	-2.373750	-0.004110
1	-2.259144	-0.275261	-2.155992
1	-4.745115	-0.201305	-2.150034
1	-5.967596	-0.171195	0.001121
1	-4.744289	-0.214022	2.151600
1	-0.925526	3.094817	-0.765308

1	-0.932451	3.093471	0.760864
1	0.892232	-1.781461	-0.003180
1	2.397646	-2.928330	-0.834983
1	2.397378	-2.931484	0.824714

Freq.
10.8781004
43.0427017
43.4840012
54.5485992
84.8561020
124.336800
134.706696
157.990295
172.186005
192.227905
221.289200
228.500198
260.339691
306.817596
393.425812
406.391113
441.985596
458.471313
465.321106
522.709595
563.424988
581.224976
582.873108
619.876709
637.498474
671.380615
696.127686
749.589111
750.312073
796.320007
799.145386
836.107727
875.020874
884.137329
925.833008
984.541382
1013.19598
1014.83130
1015.40039
1026.26953
1050.95251
1055.88867

1063.87183
1071.33594
1123.79333
1130.43665
1171.81995
1191.39050
1213.72693
1214.79480
1226.10144
1265.89233
1293.33264
1325.95435
1349.10181
1352.03052
1362.45581
1382.87964
1503.86218
1524.60559
1539.56726
1555.78503
1647.18665
1652.74255
1663.66943
1681.73474
1700.16174
1717.46106
1763.30444
2151.00708
3232.36133
3232.43994
3239.97949
3241.06641
3244.46802
3252.45020
3254.49170
3258.79590
3262.35522
3528.13184
3614.87109
3824.92529
3943.41772

TS-D

6	3.843660	0.244127	-1.024516
6	3.083276	-0.251396	0.029041
6	3.688880	-0.942440	1.072781
6	5.064139	-1.143918	1.052277
6	5.847385	-0.663722	-0.000280

6	5.218053	0.036523	-1.032637
16	1.313754	-0.024890	0.023158
8	0.888043	0.028688	1.435525
6	7.334492	-0.913395	-0.032605
8	0.779482	-1.239488	-0.688828
8	1.036584	1.206242	-0.739474
6	-1.880825	-0.569167	-0.532997
6	-2.096396	0.685842	-0.422678
6	-2.019351	2.055010	-0.186115
6	-1.179212	2.511873	0.855179
6	-1.074422	3.875537	1.080715
6	-1.801853	4.768646	0.296343
6	-2.635637	4.321117	-0.734949
6	-2.737736	2.967589	-0.989467
6	-2.744263	-1.707522	-0.251840
6	-2.322944	-3.010496	-0.519802
6	-3.205537	-4.068666	-0.347020
6	-4.503610	-3.821962	0.096805
6	-4.915064	-2.529096	0.406624
6	-4.032133	-1.461942	0.253241
7	-4.366759	-0.121354	0.543648
1	-1.307729	-3.172084	-0.868754
1	-5.916325	-2.350181	0.789635
1	-5.198654	-4.646356	0.224184
1	-2.886478	-5.082171	-0.565978
1	-0.797148	-0.797344	-0.854835
1	-3.369679	2.587564	-1.784911
1	-3.187490	5.035027	-1.337072
1	-1.714636	5.835335	0.482331
1	-0.416509	4.243843	1.860204
1	-0.591600	1.787962	1.415542
1	-5.363513	0.023070	0.661248
1	-3.886556	0.225031	1.369798
1	3.082465	-1.300203	1.898263
1	5.539273	-1.678764	1.871299
1	5.814217	0.429771	-1.852735
1	3.355467	0.801018	-1.817383
1	7.864549	-0.104083	-0.544581
1	7.746776	-1.004174	0.977185
1	7.562534	-1.844345	-0.565904

Frequencies

12.2751999
19.0156002
28.3899002
36.1521988
41.9523010
46.7587013
47.6282997

57.6189995
64.4029999
93.2684021
105.342201
131.261398
142.386597
173.463593
227.647293
232.039703
233.645905
274.461487
301.053497
320.741791
342.444092
370.703491
387.925201
393.678802
411.798798
419.714508
434.715607
464.641907
488.328400
507.920502
528.672302
539.077698
544.014099
559.715881
572.061218
581.085510
588.133118
634.192810
656.073730
663.785706
689.533630
694.833618
730.451294
742.714294
775.421082
777.773987
803.696716
821.060608
829.759827
841.270203
843.028809
872.285889
878.019714
886.165527
900.763794

916.254883
982.053589
987.575928
991.532776
996.907471
1015.35107
1023.02838
1023.26819
1027.65430
1028.11218
1047.03088
1055.87292
1063.90967
1081.18579
1081.85754
1125.03516
1132.44604
1151.40405
1153.98291
1181.02222
1187.20874
1193.93945
1206.85950
1211.14600
1226.62451
1231.88806
1254.50623
1260.20215
1274.63867
1297.45215
1339.90833
1345.72534
1355.62476
1356.84924
1366.79932
1381.20764
1388.83521
1450.11060
1462.28870
1498.99097
1521.98120
1526.53870
1528.72083
1546.75854
1563.50085
1566.96960
1646.74524
1665.82837

1668.19714
1681.58667
1686.84473
1700.71997
1711.33447
1952.70544
2317.96729
3063.91162
3132.16797
3154.09570
3202.07642
3204.10474
3208.06299
3219.81665
3224.44849
3225.93604
3235.74390
3237.19116
3244.94946
3246.50220
3247.81641
3248.03101
3252.94238
3547.90552
3648.09424

TS-A

6	1.275539	0.829954	1.581215
6	0.280859	-0.138165	1.378950
6	0.597724	-1.462532	1.724643
6	1.833137	-1.787666	2.271450
6	2.790589	-0.805942	2.482106
6	2.505823	0.509369	2.132732
6	-0.995798	0.304002	0.745688
8	-2.155046	-0.976852	0.969240
7	-0.370481	-2.540357	1.495815
7	-0.906180	-0.136017	-0.992736
6	-0.690966	-1.494135	-1.410497
6	0.630115	-1.938549	-1.603308
6	0.836294	-3.290922	-1.916724
6	-0.240002	-4.159440	-2.047238
6	-1.544022	-3.690748	-1.883821
6	-1.768991	-2.357806	-1.565605
6	1.718450	-1.032180	-1.463607
6	2.609460	-0.230614	-1.286478
6	3.635916	0.713652	-0.988925
6	4.904840	0.274076	-0.588028
6	5.876999	1.196814	-0.225340

6	5.595982	2.560958	-0.257884
6	4.338666	3.004155	-0.662438
6	3.361308	2.088072	-1.027573
6	-1.500836	1.556609	0.880951
6	-2.628470	2.126284	0.140834
6	-2.738113	3.524856	0.045481
6	-3.762964	4.118038	-0.676300
6	-4.710688	3.334426	-1.333752
6	-4.630413	1.950742	-1.240506
6	-3.611011	1.350120	-0.505228
6	-3.045158	-0.726465	2.105805
1	1.087635	1.847312	1.268535
1	-3.613861	0.272615	-0.382638
1	-5.375632	1.331369	-1.725062
1	-5.506900	3.800056	-1.899600
1	-3.823134	5.197862	-0.731586
1	-2.002222	4.146404	0.543091
1	2.047038	-2.819727	2.524384
1	3.753293	-1.067661	2.899742
1	3.253177	1.281712	2.255689
1	-1.004593	2.209142	1.587386
1	-1.441474	-1.882367	1.187039
1	-0.501313	-3.120565	2.322314
1	-0.109472	-3.130701	0.704135
1	1.848263	-3.640793	-2.070140
1	-0.064505	-5.197655	-2.296040
1	-2.383163	-4.362191	-2.007074
1	-2.774357	-1.993365	-1.405618
1	2.384211	2.429652	-1.347208
1	4.123274	4.064317	-0.698577
1	6.357074	3.277171	0.023992
1	6.856681	0.852874	0.080439
1	5.116727	-0.787014	-0.559479
1	-1.787712	0.256406	-1.332274
1	-0.128593	0.464232	-1.273444
6	-4.326456	-1.510569	1.909478
1	-2.525264	-1.015198	3.027160
1	-3.222789	0.349122	2.137203
1	-5.000129	-1.335528	2.752358
1	-4.836787	-1.191450	0.998262
1	-4.135346	-2.584973	1.845043

Frequencies

13.9820995
27.5160999
34.3732986
43.0978012
45.0107002
55.7438011

62.3535995
71.6653976
86.8645020
89.7032013
99.1379013
118.805801
121.080803
148.017105
167.655304
169.772995
190.803894
216.191498
220.997803
223.867798
249.783096
260.791687
273.552002
301.965912
306.571014
324.711212
374.108398
381.064087
396.278503
420.491608
426.528198
432.812195
447.758698
469.468903
479.322693
500.387085
521.161316
539.445190
553.831421
565.013306
572.446716
575.885193
588.646790
602.840088
612.609070
617.159729
629.002197
635.094482
650.424011
665.285522
668.533997
692.530701
728.443970
738.624512

755.942505
761.929810
768.744324
790.388977
797.803772
799.164124
815.724487
816.233582
820.973816
827.990784
842.458191
853.944824
877.253723
882.366882
898.433289
905.960815
912.976990
936.135681
937.661926
940.693481
987.769897
989.067078
1002.29077
1023.57239
1028.51050
1042.61951
1043.04895
1052.07410
1052.69470
1068.72925
1072.33777
1074.39197
1077.04639
1079.34851
1081.70532
1084.50659
1089.79761
1101.88086
1115.84277
1140.71167
1144.37317
1149.93420
1153.20862
1175.23535
1210.92065
1213.58105
1230.85864
1232.77661

1243.16382
1244.96924
1245.31494
1246.72180
1251.80774
1255.45618
1259.55652
1262.18188
1269.14575
1289.04871
1302.76685
1318.30762
1340.73999
1343.88977
1350.89343
1355.10168
1367.92944
1375.05139
1380.69714
1403.91541
1407.12305
1417.69946
1446.43359
1482.69812
1523.33435
1523.47583
1526.18262
1532.13086
1548.48364
1551.45911
1562.30493
1564.73816
1572.53625
1579.44922
1581.27161
1624.31531
1655.60645
1658.66711
1659.94580
1662.88953
1673.67053
1689.44958
1690.54004
1691.20703
1693.13416
1699.54834
1715.82312
1811.10840

2389.26611
3058.72559
3071.70850
3147.17871
3162.71094
3171.37622
3207.03955
3209.35474
3216.68213
3216.94067
3224.15479
3224.85474
3230.47559
3231.58984
3233.18970
3233.48169
3239.00757
3241.85474
3243.13306
3248.37158
3251.76221
3252.25171
3252.97144
3260.51221
3262.43579
3442.91699
3489.29956
3534.53613
3585.12793

TS-B

6	3.946509	-1.253942	0.454520
6	3.084735	-1.690261	-0.564845
6	3.544316	-1.630404	-1.885216
6	4.796793	-1.107340	-2.193950
6	5.618758	-0.643392	-1.172294
6	5.198761	-0.729068	0.151613
6	1.702282	-2.143676	-0.268689
6	0.701116	-1.310243	0.053920
6	-0.725090	-1.645636	0.196294
6	-1.433518	-2.009902	-0.957698
6	-2.770420	-2.376357	-0.876453
6	-3.415244	-2.385126	0.356561
6	-2.714042	-2.040746	1.507906
6	-1.375489	-1.675146	1.431588
7	3.485822	-1.312652	1.813880
8	1.161698	-0.273198	1.436683
7	0.753320	0.200949	-1.135545

6	1.125748	1.529735	-0.761425
6	0.111071	2.463048	-0.488333
6	0.477928	3.759249	-0.109194
6	1.818013	4.106306	0.004064
6	2.810473	3.165155	-0.265270
6	2.466551	1.875234	-0.651993
6	-1.251174	2.044219	-0.546660
6	-2.374245	1.588019	-0.561877
6	-3.688517	1.027868	-0.549927
6	-4.404458	0.944027	0.651214
6	-5.666286	0.364719	0.670603
6	-6.224560	-0.136032	-0.503219
6	-5.519688	-0.050442	-1.701600
6	-4.258092	0.529196	-1.728818
1	-0.825390	-1.435663	2.328996
6	0.564517	0.838651	2.180909
1	-0.925637	-2.031677	-1.916205
1	5.854023	-0.395837	0.948487
1	6.594257	-0.234017	-1.399811
1	5.129076	-1.065736	-3.222795
1	2.904179	-2.005514	-2.676679
1	-3.210504	-2.060642	2.469395
1	-4.462170	-2.650050	0.416856
1	-3.310015	-2.651907	-1.772579
1	1.450366	-3.194604	-0.356902
1	-0.298740	4.483602	0.096117
1	2.089634	5.111482	0.297664
1	3.854289	3.437888	-0.182435
1	3.235410	1.143029	-0.866504
1	-3.708751	0.602268	-2.658958
1	-5.958040	-0.427991	-2.616641
1	-7.211134	-0.581793	-0.486340
1	-6.217689	0.307840	1.600482
1	-3.964568	1.331276	1.561338
1	1.398032	-0.200624	-1.812730
1	-0.211299	0.167611	-1.470165
1	2.113363	-0.584674	1.722119
1	3.181015	-2.242674	2.092495
1	4.146009	-0.934656	2.484414
6	0.646217	0.571200	3.672666
1	1.095093	1.753019	1.909363
1	-0.465981	0.911777	1.832095
1	0.198087	1.404567	4.220251
1	0.116013	-0.342327	3.950623
1	1.685705	0.480049	3.999864

Frequencies

16.0876999

33.1953011
43.0704002
44.7006989
47.3162994
61.0769005
67.1619034
79.3986969
93.3730011
101.165100
103.892998
110.158897
134.458694
139.373993
146.936005
164.156601
171.410202
193.714706
213.069901
230.662903
238.925507
255.087601
270.409790
297.615692
303.478394
340.039612
349.240509
372.847412
379.495514
398.367096
405.493103
425.292603
435.359894
444.688690
458.654602
475.114197
498.611206
513.476013
518.919312
540.190125
547.843201
562.208679
573.851685
585.362610
593.968689
612.190308
617.421692
624.269104
637.338501

663.203674
668.211487
672.752625
730.328796
737.563416
744.568726
763.291870
767.529785
778.233398
802.635315
807.483582
811.110291
818.110596
819.895630
826.011414
841.793213
869.597290
875.894226
895.888000
896.344482
904.178223
908.874084
924.912903
943.284912
952.140991
988.281799
992.708679
996.343506
1025.59546
1027.42749
1039.17090
1043.25745
1053.03235
1053.41113
1059.99268
1069.82996
1073.16870
1075.61096
1077.09424
1082.09363
1083.60608
1090.18933
1092.14941
1129.84277
1139.31555
1140.04382
1144.60669
1149.59937

1181.34778
1186.42908
1204.56006
1210.81226
1215.99695
1224.57202
1238.04468
1243.82629
1245.55212
1246.42676
1246.92456
1255.12793
1256.56421
1258.09155
1286.81873
1298.77307
1317.44556
1345.27734
1346.14136
1348.65112
1351.47729
1362.29395
1375.22852
1391.28455
1398.23535
1403.60205
1409.99695
1430.38293
1479.56152
1522.79395
1523.32776
1530.07166
1536.12756
1544.63293
1553.86890
1563.69824
1566.83472
1570.20557
1572.48120
1581.50757
1645.54639
1660.47290
1662.54883
1663.79614
1665.97620
1681.20154
1686.27942
1692.31360

1694.21875
1702.41504
1715.70959
1742.61938
2388.26880
2455.83472
3070.64868
3109.06372
3148.12402
3161.26660
3181.64600
3203.18164
3206.86792
3211.73560
3215.36841
3218.05811
3223.55835
3227.91602
3230.38989
3232.78345
3236.02368
3236.30200
3240.21851
3242.00952
3246.01489
3250.54028
3252.69019
3257.66821
3258.34106
3279.22095
3467.49951
3532.22437
3584.21509
3643.65210

TS-D

6	-3.848494	-0.240003	-1.027859
6	-3.088540	0.250831	0.028201
6	-3.694693	0.936406	1.075224
6	-5.070071	1.137150	1.055530
6	-5.852896	0.661623	0.000542
6	-5.223012	-0.033193	-1.035141
16	-1.318882	0.025426	0.021437
8	-0.893314	-0.034787	1.433580
6	-7.340149	0.910551	-0.030752
8	-0.785257	1.243809	-0.684544
8	-1.040871	-1.201800	-0.747161
6	1.875436	0.574344	-0.531649

6	2.091754	-0.681058	-0.427430
6	2.015508	-2.051411	-0.197563
6	1.175517	-2.513860	0.841382
6	1.071525	-3.878671	1.060245
6	1.799593	-4.767500	0.271616
6	2.633234	-4.314439	-0.757374
6	2.734545	-2.959623	-1.005269
6	2.738150	1.711835	-0.244829
6	2.316076	3.015846	-0.506482
6	3.198006	4.073694	-0.328425
6	4.496173	3.825611	0.114355
6	4.908372	2.531497	0.417912
6	4.026106	1.464571	0.259210
7	4.361507	0.122784	0.543113
1	1.300806	3.178522	-0.854770
1	5.909693	2.351321	0.800172
1	5.190703	4.649794	0.245846
1	2.878362	5.088063	-0.542473
1	0.791661	0.803433	-0.852508
1	3.366356	-2.575338	-1.798768
1	3.185594	-5.025067	-1.362905
1	1.712999	-5.835137	0.452384
1	0.413738	-4.251176	1.837844
1	0.587397	-1.793048	1.405197
1	5.358334	-0.021609	0.660132
1	3.881412	-0.227919	1.367500
1	-3.088596	1.290502	1.902520
1	-5.545630	1.667704	1.877093
1	-5.818835	-0.422794	-1.857224
1	-3.359866	-0.792722	-1.823373
1	-7.869653	0.103428	-0.546740
1	-7.752613	0.996151	0.979417
1	-7.568689	1.843956	-0.559530

Frequencies
12.2751999
19.0156002
28.3899002
36.1521988
41.9523010
46.7587013
47.6282997
57.6189995
64.4029999
93.2684021
105.342201
131.261398
142.386597

173.463593
227.647293
232.039703
233.645905
274.461487
301.053497
320.741791
342.444092
370.703491
387.925201
393.678802
411.798798
419.714508
434.715607
464.641907
488.328400
507.920502
528.672302
539.077698
544.014099
559.715881
572.061218
581.085510
588.133118
634.192810
656.073730
663.785706
689.533630
694.833618
730.451294
742.714294
775.421082
777.773987
803.696716
821.060608
829.759827
841.270203
843.028809
872.285889
878.019714
886.165527
900.763794
916.254883
982.053589
987.575928
991.532776
996.907471
1015.35107

1023.02838
1023.26819
1027.65430
1028.11218
1047.03088
1055.87292
1063.90967
1081.18579
1081.85754
1125.03516
1132.44604
1151.40405
1153.98291
1181.02222
1187.20874
1193.93945
1206.85950
1211.14600
1226.62451
1231.88806
1254.50623
1260.20215
1274.63867
1297.45215
1339.90833
1345.72534
1355.62476
1356.84924
1366.79932
1381.20764
1388.83521
1450.11060
1462.28870
1498.99097
1521.98120
1526.53870
1528.72083
1546.75854
1563.50085
1566.96960
1646.74524
1665.82837
1668.19714
1681.58667
1686.84473
1700.71997
1711.33447
1952.70544

2317.96729
3063.91162
3132.16797
3154.09570
3202.07642
3204.10474
3208.06299
3219.81665
3224.44849
3225.93604
3235.74390
3237.19116
3244.94946
3246.50220
3247.81641
3248.03101
3252.94238
3547.90552
3648.09424

TS-E

6	0.746501	-1.391649	1.314410
6	0.552114	-2.750386	1.082528
1	-0.225783	-3.059326	0.396478
6	1.375241	-3.681216	1.704904
1	1.225239	-4.737869	1.523410
6	2.401591	-3.259126	2.547493
1	3.047798	-3.987079	3.021728
6	2.598872	-1.902263	2.787604
1	3.391194	-1.571228	3.448494
6	1.765803	-0.967659	2.183447
7	1.908567	0.439269	2.349503
1	1.380002	0.871006	3.096124
1	2.841076	0.815754	2.248677
6	0.234075	0.922281	0.847313
6	0.025882	2.317228	0.788778
6	-1.251976	2.838337	1.071973
1	-2.057761	2.157059	1.316648
6	-1.473461	4.206186	0.998771
1	-2.462052	4.599492	1.199250
6	-0.430774	5.071123	0.673969
1	-0.607637	6.138427	0.626651
6	0.839848	4.562464	0.400605
1	1.646478	5.235287	0.137258
6	1.069183	3.197110	0.443633
1	2.038281	2.784096	0.194277
6	0.025073	-0.326640	0.626724
6	-0.857294	-0.687921	-0.647730

6	-0.154838	-0.838036	-1.749460
6	1.196722	-0.490935	-2.063086
6	1.566681	0.860390	-2.231912
1	0.802265	1.623670	-2.150249
6	2.883854	1.209423	-2.500048
1	3.143364	2.254213	-2.633875
6	3.869723	0.229420	-2.598583
1	4.896719	0.503781	-2.807741
6	3.517215	-1.111151	-2.439504
1	4.276480	-1.881205	-2.517105
6	2.201723	-1.471362	-2.187498
1	1.931301	-2.513380	-2.067129
6	-2.295487	-0.848131	-0.384471
6	-3.193161	-1.152094	-1.447424
7	-2.732575	-1.322844	-2.719887
6	-4.564638	-1.274337	-1.157998
1	-5.247247	-1.500787	-1.970172
6	-5.048767	-1.112467	0.128508
1	-6.111862	-1.211426	0.315197
6	-4.175356	-0.829194	1.176193
1	-4.543867	-0.711608	2.187408
6	-2.819485	-0.705178	0.905343
1	-2.136534	-0.491706	1.719223
1	-1.725565	-1.278526	-2.869661
1	-3.363018	-1.538934	-3.469083

Frequencies
15.0548000
24.4934006
33.0746002
40.0217018
44.8124008
48.3056984
54.1073990
61.5348015
76.7870026
121.795502
136.603394
146.495605
156.063705
185.587997
201.324295
248.124695
260.170502
264.884705
274.308594
297.633698
368.975586

412.111694
424.218994
426.126404
431.895691
434.434906
455.495789
469.192596
486.084503
502.150299
514.547974
542.088013
544.733521
559.489014
569.195679
580.292419
584.441101
607.043823
613.948792
643.332520
656.257874
664.577393
670.121521
674.065430
700.020325
722.460510
727.657776
734.668396
741.077881
777.565613
789.242004
792.132874
800.138123
802.699219
807.130676
818.640076
839.693298
854.130920
861.938171
890.351318
900.047485
902.632996
916.249878
927.989014
938.193298
957.010376
987.464600
988.231079
1011.57568

1026.99170
1029.17871
1041.87183
1043.50317
1047.95947
1049.82007
1061.51123
1070.51025
1077.03259
1078.70752
1083.43665
1088.17566
1120.19678
1131.84607
1140.39014
1157.94958
1188.95764
1198.25696
1213.64075
1224.26575
1233.34851
1239.94299
1240.18982
1240.92651
1248.79724
1255.01025
1287.63965
1312.02393
1328.34338
1338.73376
1347.64722
1355.28564
1361.03601
1372.34717
1385.28088
1398.40149
1404.86584
1409.94788
1513.92224
1518.49976
1535.32581
1536.39160
1556.94971
1557.60437
1564.30334
1575.37476
1640.82410
1646.20862

1647.15601
1656.03674
1669.17676
1679.43860
1682.71008
1691.91870
1699.69336
1707.66455
1749.98474
1937.05090
3191.76392
3192.41919
3202.30371
3206.34009
3211.28882
3213.60327
3215.73193
3220.12012
3221.96704
3223.54468
3227.26392
3230.87549
3235.06909
3235.45874
3237.10791
3240.00195
3247.96460
3249.03687
3396.62378
3581.61011
3693.99951
3750.38721

IV. Details of NCI analysis and RESP analysis

In the Figure S1 we report the comparison of the NCI 3D representations for the TSs involved in the initial 1a-Ia addition (see Figure 2A of the main text).

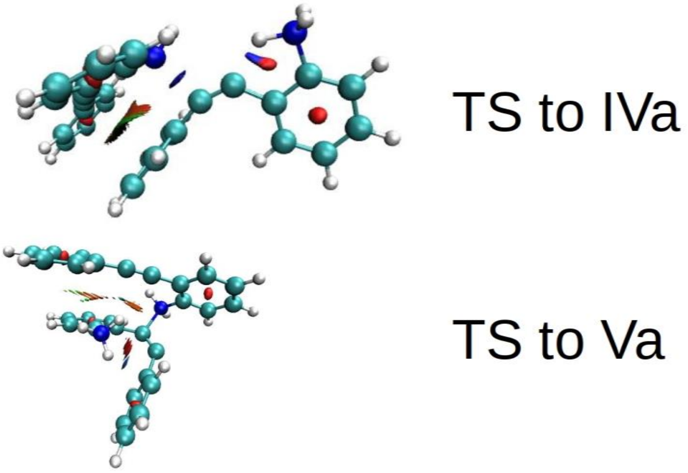

Figure S1. NCI 3D representations of the TSs involved in the 1a to Ia addition.

We observe that the two TSs are rather different. In fact, the two C-N covalent bonds, involved in the reaction, are both recognized as non-covalent strong interactions in the TS leading to Iva. On the other hand, the same C-N bonds are recognized as covalent strong, hence stronger, interactions in the TS leading to Va.

NCI analysis carried in the two high-energy TSs of the Figure 3 (i.e. TS-A and TS-B) reported in the Figure S2 shows large regions of sharp repulsive clash between the two interacting moieties. In the less stable TS-A the repulsive region appears as much larger than in TS-B. This finding could somewhat explain the difference between the two observed barriers.

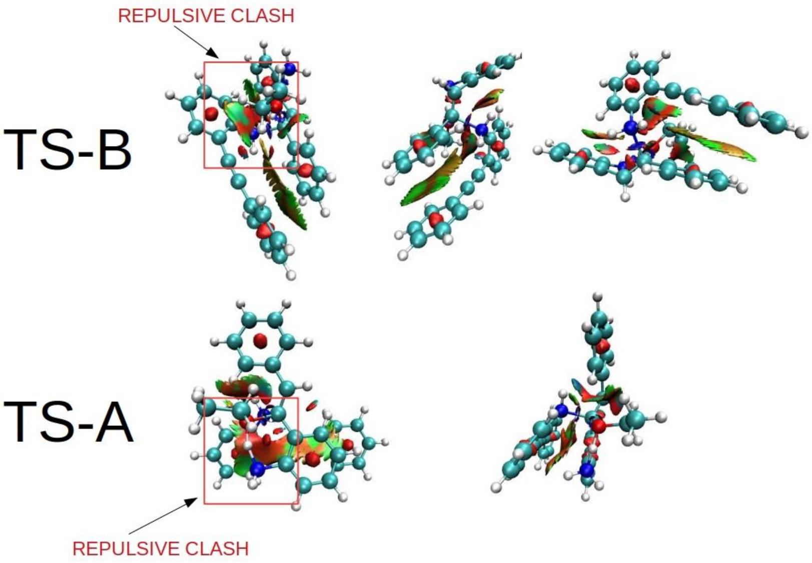

Figure S2. NCI 3D representations for TS-A (2 different views) and TS-B (three different views).

NCI analysis carried also on the two TS-D involved in the formation of 5a from 1a (see Figure 4 of the main text) produced the result reported in the Figure S3.

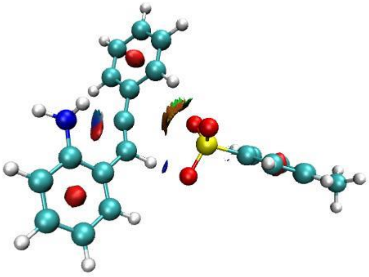

Figure S3. NCI 3D representation of TS-D (see Figure 4 of the main text).

The result nicely indicates the simultaneous presence of two strongly attractive (blue region) NCI in the regions corresponding to the proton-transfer and the ring-closure.

In the Figure S4 we report the results of the RESP analysis carried out on some of the key intermediates of the reactions reported in the Figures 2 and 3 of the main text.

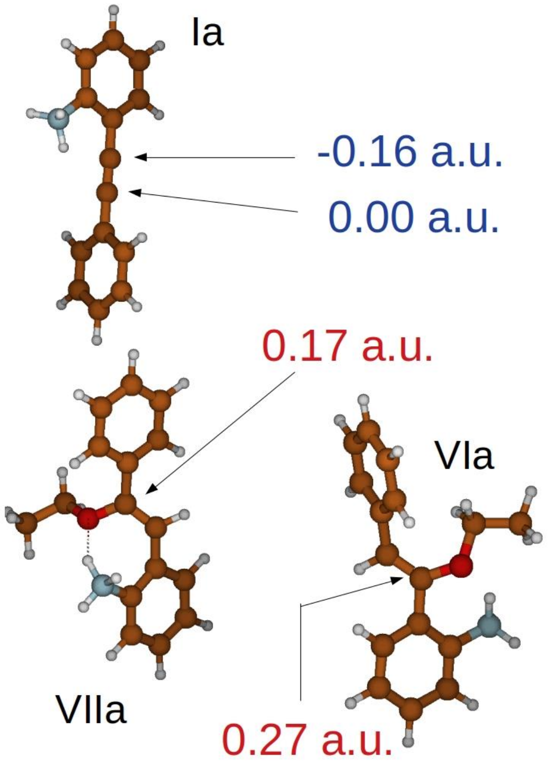

Figure S4. RESP charges for the species Ia, VIa and VIIa
